# 6-month neurological and psychiatric outcomes in 236 379 survivors of COVID-19: a retrospective cohort study using electronic health records

**DOI:** 10.1016/S2215-0366(21)00084-5

**Published:** 2021-05

**Authors:** Maxime Taquet, John R Geddes, Masud Husain, Sierra Luciano, Paul J Harrison

**Affiliations:** a Department of Psychiatry, University of Oxford, Oxford, UK; b Nuffield Department of Clinical Neurosciences, University of Oxford, Oxford, UK; c Oxford Health NHS Foundation Trust, Oxford, UK; d Oxford University Hospitals NHS Foundation Trust, Oxford, UK; e TriNetX, Cambridge MA, USA

## Abstract

**Background:**

Neurological and psychiatric sequelae of COVID-19 have been reported, but more data are needed to adequately assess the effects of COVID-19 on brain health. We aimed to provide robust estimates of incidence rates and relative risks of neurological and psychiatric diagnoses in patients in the 6 months following a COVID-19 diagnosis.

**Methods:**

For this retrospective cohort study and time-to-event analysis, we used data obtained from the TriNetX electronic health records network (with over 81 million patients). Our primary cohort comprised patients who had a COVID-19 diagnosis; one matched control cohort included patients diagnosed with influenza, and the other matched control cohort included patients diagnosed with any respiratory tract infection including influenza in the same period. Patients with a diagnosis of COVID-19 or a positive test for SARS-CoV-2 were excluded from the control cohorts. All cohorts included patients older than 10 years who had an index event on or after Jan 20, 2020, and who were still alive on Dec 13, 2020. We estimated the incidence of 14 neurological and psychiatric outcomes in the 6 months after a confirmed diagnosis of COVID-19: intracranial haemorrhage; ischaemic stroke; parkinsonism; Guillain-Barré syndrome; nerve, nerve root, and plexus disorders; myoneural junction and muscle disease; encephalitis; dementia; psychotic, mood, and anxiety disorders (grouped and separately); substance use disorder; and insomnia. Using a Cox model, we compared incidences with those in propensity score-matched cohorts of patients with influenza or other respiratory tract infections. We investigated how these estimates were affected by COVID-19 severity, as proxied by hospitalisation, intensive therapy unit (ITU) admission, and encephalopathy (delirium and related disorders). We assessed the robustness of the differences in outcomes between cohorts by repeating the analysis in different scenarios. To provide benchmarking for the incidence and risk of neurological and psychiatric sequelae, we compared our primary cohort with four cohorts of patients diagnosed in the same period with additional index events: skin infection, urolithiasis, fracture of a large bone, and pulmonary embolism.

**Findings:**

Among 236 379 patients diagnosed with COVID-19, the estimated incidence of a neurological or psychiatric diagnosis in the following 6 months was 33·62% (95% CI 33·17–34·07), with 12·84% (12·36–13·33) receiving their first such diagnosis. For patients who had been admitted to an ITU, the estimated incidence of a diagnosis was 46·42% (44·78–48·09) and for a first diagnosis was 25·79% (23·50–28·25). Regarding individual diagnoses of the study outcomes, the whole COVID-19 cohort had estimated incidences of 0·56% (0·50–0·63) for intracranial haemorrhage, 2·10% (1·97–2·23) for ischaemic stroke, 0·11% (0·08–0·14) for parkinsonism, 0·67% (0·59–0·75) for dementia, 17·39% (17·04–17·74) for anxiety disorder, and 1·40% (1·30–1·51) for psychotic disorder, among others. In the group with ITU admission, estimated incidences were 2·66% (2·24–3·16) for intracranial haemorrhage, 6·92% (6·17–7·76) for ischaemic stroke, 0·26% (0·15–0·45) for parkinsonism, 1·74% (1·31–2·30) for dementia, 19·15% (17·90–20·48) for anxiety disorder, and 2·77% (2·31–3·33) for psychotic disorder. Most diagnostic categories were more common in patients who had COVID-19 than in those who had influenza (hazard ratio [HR] 1·44, 95% CI 1·40–1·47, for any diagnosis; 1·78, 1·68–1·89, for any first diagnosis) and those who had other respiratory tract infections (1·16, 1·14–1·17, for any diagnosis; 1·32, 1·27–1·36, for any first diagnosis). As with incidences, HRs were higher in patients who had more severe COVID-19 (eg, those admitted to ITU compared with those who were not: 1·58, 1·50–1·67, for any diagnosis; 2·87, 2·45–3·35, for any first diagnosis). Results were robust to various sensitivity analyses and benchmarking against the four additional index health events.

**Interpretation:**

Our study provides evidence for substantial neurological and psychiatric morbidity in the 6 months after COVID-19 infection. Risks were greatest in, but not limited to, patients who had severe COVID-19. This information could help in service planning and identification of research priorities. Complementary study designs, including prospective cohorts, are needed to corroborate and explain these findings.

**Funding:**

National Institute for Health Research (NIHR) Oxford Health Biomedical Research Centre.

**Research in context**

**Evidence before this study**

We searched Web of Science and Medline on Aug 1 and Dec 31, 2020, for studies in English, with the terms “(COVID-19 OR SARS-CoV2 OR SARS-CoV-2) AND (psychiatri* or neurologi*) AND (incidence OR epidemiologi* OR ‘systematic review’ or ‘meta-analysis’)”. We found case series and reviews of series reporting neurological and neuropsychiatric disorders during acute COVID-19 illness. We found one large electronic health records study of the psychiatric sequelae in the 3 months after a COVID-19 diagnosis. It reported an increased risk for anxiety and mood disorders and dementia after COVID-19 compared with a range of other health events; the study also reported the incidence of each disorder. We are not aware of any large-scale data regarding the incidence or relative risks of neurological diagnoses in patients who had recovered from COVID-19.

**Added value of this study**

To our knowledge, we provide the first meaningful estimates of the risks of major neurological and psychiatric conditions in the 6 months after a COVID-19 diagnosis, using the electronic health records of over 236 000 patients with COVID-19. We report their incidence and hazard ratios compared with patients who had had influenza or other respiratory tract infections. We show that both incidence and hazard ratios were greater in patients who required hospitalisation or admission to the intensive therapy unit (ITU), and in those who had encephalopathy (delirium and other altered mental states) during the illness compared with those who did not.

**Implications of all the available evidence**

COVID-19 was robustly associated with an increased risk of neurological and psychiatric disorders in the 6 months after a diagnosis. Given the size of the pandemic and the chronicity of many of the diagnoses and their consequences (eg, dementia, stroke, and intracranial haemorrhage), substantial effects on health and social care systems are likely to occur. Our data provide important evidence indicating the scale and nature of services that might be required. The findings also highlight the need for enhanced neurological follow-up of patients who were admitted to ITU or had encephalopathy during their COVID-19 illness.

## Introduction

Since the COVID-19 pandemic began on March 11, 2020, there has been concern that survivors might be at an increased risk of neurological disorders. This concern, initially based on findings from other coronaviruses,^1^ was followed rapidly by case series,^2^, ^3^, ^4^ emerging evidence of COVID-19 CNS involvement,^5^, ^6^, ^7^ and the identification of mechanisms by which this could occur.^8^, ^9^, ^10^, ^11^ Similar concerns have been raised regarding psychiatric sequelae of COVID-19,^12^, ^13^ with evidence showing that survivors are indeed at increased risk of mood and anxiety disorders in the 3 months after infection.^14^ However, we need large scale, robust, and longer term data to properly identify and quantify the consequences of the COVID-19 pandemic on brain health. Such information is required both to plan services and identify research priorities.

In this study, we used an electronic health records network to investigate the incidence of neurological and psychiatric diagnoses in survivors in the 6 months after documented clinical COVID-19 infection, and we compared the associated risks with those following other health conditions. We explored whether the severity of COVID-19 infection, as proxied by hospitalisation, intensive therapy unit (ITU) admission, and encephalopathy, affects these risks. We also assessed the trajectory of hazard ratios (HRs) across the 6-month period.

## Methods

### Study design and data collection

For this retrospective cohort study, we used The TriNetX Analytics Network, a federated network recording anonymised data from electronic health records in 62 health-care organisations, primarily in the USA, comprising 81 million patients. Available data include demographics, diagnoses (using codes from ICD-10), medications, procedures, and measurements (eg, blood pressure and body-mass index). The health-care organisations are a mixture of hospitals, primary care, and specialist providers, contributing data from uninsured and insured patients. These organisations warrant that they have all necessary rights, consents, approvals, and authority to provide the data to TriNetX, so long as their name remains anonymous as a data source and their data are used for research purposes. By use of the TriNetX user interface, cohorts can be created on the basis of inclusion and exclusion criteria, matched for confounding variables with a built-in propensity score-matching algorithm, and compared for outcomes of interest over specified time periods. Additional details about TriNetX, its data, provenance, and functionalities, are presented in the appendix (pp 1–2).

### Cohorts

The primary cohort was defined as all patients who had a confirmed diagnosis of COVID-19 (ICD-10 code U07.1). We also constructed two matched control cohorts: patients diagnosed with influenza (ICD-10 codes J09–11) and patients diagnosed with any respiratory tract infection including influenza (ICD-10 codes J00–06, J09–18, or J20–22). We excluded patients with a diagnosis of COVID-19 or a positive test for SARS-CoV-2 from the control cohorts. We refer to the diagnosis of COVID-19 (in the primary cohort) and influenza or other respiratory tract infections (in the control cohorts) as index events. The cohorts included all patients older than 10 years who had an index event on or after Jan 20, 2020 (the date of the first recorded COVID-19 case in the USA), and who were still alive at the time of the main analysis (Dec 13, 2020). Additional details on cohorts are provided in the appendix (pp 2–3).

### Covariates

We used a set of established and suspected risk factors for COVID-19 and for more severe COVID-19 illness:^15^, ^16^ age, sex, race, ethnicity, obesity, hypertension, diabetes, chronic kidney disease, asthma, chronic lower respiratory diseases, nicotine dependence, substance use disorder, ischaemic heart disease and other forms of heart disease, socioeconomic deprivation, cancer (and haematological cancer in particular), chronic liver disease, stroke, dementia, organ transplant, rheumatoid arthritis, lupus, psoriasis, and disorders involving an immune mechanism. To capture these risk factors in patients' health records, we used 55 variables. More details, including ICD-10 codes, are provided in the appendix (pp 3–4). Cohorts were matched for all these variables, as described in the following subsections.

### Outcomes

We investigated neurological and psychiatric sequelae of COVID-19 in terms of 14 outcomes occurring 1–180 days after the index event: intracranial haemorrhage (ICD-10 codes I60–62); ischaemic stroke (I63); Parkinson's disease and parkinsonism (G20–21); Guillain-Barré syndrome (G61.0); nerve, nerve root, and plexus disorders (G50–59); myoneural junction and muscle disease (neuromuscular disorders; G70–73); encephalitis (G04, G05, A86, or A85.8); dementia (F01–03, G30, G31.0, or G31.83); psychotic, mood, and anxiety disorders (F20–48), as well as each category separately; substance use disorder (F10–19), and insomnia (F51.0 or G47.0).

For outcomes that are chronic illnesses (eg, dementia or Parkinson's disease), we excluded patients who had the diagnosis before the index event. For outcomes that tend to recur or relapse (eg, ischaemic strokes or psychiatric diagnoses), we estimated separately the incidence of first diagnoses (ie, excluding those who had a diagnosis before the index event) and the incidence of any diagnosis (ie, including patients who had a diagnosis at some point before the index event). For other outcomes (eg, Guillain-Barré syndrome), we estimated the incidence of any diagnosis. More details, and a full list of ICD-10 codes, are provided in the appendix (pp 4–5).

Finally, to assess the overall risk of neurological and psychiatric outcomes after COVID-19, we estimated the incidence of any of the 14 outcomes, and the incidence of a first diagnosis of any of the outcomes. This is lower than the sum of incidences of each outcome because some patients had more than one diagnosis.

### Secondary analyses

We investigated whether the neurological and psychiatric sequelae of COVID-19 were affected by the severity of the illness. The incidence of outcomes was estimated separately in four subgroups: first, in those who had required hospitalisation within a time window from 4 days before their COVID-19 diagnosis (taken to be the time it might take between clinical presentation and confirmation) to 2 weeks afterwards; second, in those who had not required hospitalisation during that window; third, in those who had been admitted to an intensive therapy unit (ITU) during that window; and fourth, in those who were diagnosed with delirium or other forms of altered mental status during that window; we use the term encephalopathy to describe this group of patients (appendix p 5).^17^, ^18^

Differences in outcome incidence between these subgroups might reflect differences in their baseline characteristics. Therefore, for each outcome, we estimated the HR between patients requiring hospitalisation (or ITU) and a matched cohort of patients not requiring hospitalisation (or ITU), and between patients with encephalopathy and a matched cohort of patients without encephalopathy. Finally, HRs were calculated for patients who had not required hospitalisation for COVID-19, influenza, or other respiratory tract infections.

To provide benchmarks for the incidence and risk of neurological and psychiatric sequelae, patients after COVID-19 were compared with those in four additional matched cohorts of patients diagnosed with health events selected to represent a range of acute presentations during the same time period. These additional four index events were skin infection, urolithiasis, fracture of a large bone, and pulmonary embolism. More details are presented in the appendix (pp 5–6).

We assessed the robustness of the differences in outcomes between cohorts by repeating the analysis in three scenarios: one including patients who had died by the time of the analysis, another restricting the COVID-19 diagnoses to patients who had a positive RNA or antigen test (and using antigen test as an index event), and another comparing the rates of sequelae of patients with COVID-19 with those observed in patients with influenza before the pandemic (ie, in 2019 or 2018). Details of these analyses are provided in the appendix (p 6).

Finally, to test whether differences in sequelae between cohorts could be accounted for by differences in extent of follow-up, we counted the average number of health visits that each cohort had during the follow-up period.

### Statistical analysis

We used propensity score matching^19^ to create cohorts with matched baseline characteristics, done within the TriNetX network. Propensity score with 1:1 matching used a greedy nearest neighbour matching approach with a calliper distance of 0·1 pooled SDs of the logit of the propensity score. Any characteristic with a standardised mean difference between cohorts lower than 0·1 was considered well matched.^20^ The incidence of each outcome was estimated by use of the Kaplan-Meier estimator. Comparisons between cohorts were made with a log-rank test. We calculated HRs with 95% CIs using a proportional hazard model wherein the cohort to which the patient belonged was used as the independent variable. The proportional hazard assumption was tested with the generalised Schoenfeld approach. When the assumption was violated, the time-varying HR was assessed with natural cubic splines fitted to the log cumulative hazard.^21^ Additional details are presented in the appendix (p 6). Statistical analyses were done in R, version 3.4.3, except for the log-rank tests, which were done within TriNetX. Statistical significance was set at two-sided p-value <0·05. Our study was reported according to the Reporting of studies Conducted using Observational Routinely-collected health Data (RECORD, appendix pp 55–60).

### Role of the funding source

The funder of the study had no role in study design, data collection, data analysis, data interpretation, or writing of the manuscript.

## Results

Our primary cohort comprised 236 379 patients diagnosed with COVID-19, and our two propensity-score-matched control cohorts comprised 105 579 patients diagnosed with influenza and 236 038 patients diagnosed with any respiratory tract infection including influenza. The COVID-19 cohort was divided into subgroups of patients who were not hospitalised (190 077 patients), those who were hospitalised (46 302 patients), those who required ITU admission (8945 patients), and those who received a diagnosis of encephalopathy (6229 patients). The main demographic features and comorbidities of the COVID-19 cohort are summarised in table 1, with additional demographic details presented in the appendix (pp 25–27). Matched baseline characteristics of the two control cohorts are also presented in the appendix (pp 29–30 for patients with influenza, and pp 31–32 for patients with other respiratory tract infections). Adequate propensity-score matching (standardised mean difference <0·1) was achieved for all comparisons and baseline characteristics.

**Table 1:** Baseline characteristics for the whole COVID-19 cohort and for the non-hospitalisation, hospitalisation, ITU admission, and encephalopathy cohorts during the illness

		**All patients**	**Patients without hospitalisation**	**Patients with hospitalisation**	**Patients with ITU admission**	**Patients with encephalopathy**
Cohort size		236 379 (100·0%)	190 077 (100·0%)	46 302 (100·0%)	8945 (100·0%)	6229 (100·0%)
Demographics						
	Age, years	46 (19·7)	43·3 (19·0)	57 (18·7)	59·1 (17·3)	66·7 (17·0)
Sex						
	Male	104 015 (44·0%)	81 512 (42·9%)	22 503 (48·6%)	5196 (58·1%)	3307 (53·1%)
	Female	131 460 (55·6%)	107 730 (56·7%)	23 730 (51·3%)	3743 (41·8%)	2909 (46·7%)
	Other	904 (0·4%)	835 (0·4%)	69 (0·1%)	10 (0·1%)	13 (0·2%)
Race						
	White	135 143 (57·2%)	109 635 (57·7%)	25 508 (55·1%)	4918 (55·0%)	3331 (53·5%)
	Black or African American	44 459 (18·8%)	33 868 (17·8%)	10 591 (22·9%)	2184 (24·4%)	1552 (24·9%)
	Unknown	48 085 (20·3%)	39 841 (21·0%)	8244 (17·8%)	1457 (16·3%)	1071 (17·2%)
Ethnicity						
	Hispanic or Latino	37 772 (16·0%)	29 155 (15·3%)	8617 (18·6%)	2248 (25·1%)	895 (14·4%)
	Not Hispanic or Latino	134 075 (56·7%)	106 844 (56·2%)	27 231 (58·8%)	5041 (56·4%)	3873 (62·2%)
	Unknown	64 532 (27·3%)	54 078 (28·5%)	10 454 (22·6%)	1656 (18·5%)	1461 (23·5%)
Comorbidities						
	Overweight and obesity	42 871 (18·1%)	30 198 (15·9%)	12 673 (27·4%)	3062 (34·2%)	1838 (29·5%)
	Hypertensive disease	71 014 (30·0%)	47 516 (25·0%)	23 498 (50·7%)	5569 (62·3%)	4591 (73·7%)
	Type 2 diabetes	36 696 (15·5%)	22 518 (11·8%)	14 178 (30·6%)	3787 (42·3%)	2890 (46·4%)
	Asthma	25 104 (10·6%)	19 834 (10·4%)	5270 (11·4%)	1132 (12·7%)	755 (12·1%)
	Nicotine dependence	17 105 (7·2%)	12 639 (6·6%)	4466 (9·6%)	1042 (11·6%)	803 (12·9%)
	Substance use disorder	24 870 (10·5%)	18 173 (9·6%)	6697 (14·5%)	1620 (18·1%)	1316 (21·1%)
	Ischaemic heart diseases	21 082 (8·9%)	11 815 (6·2%)	9267 (20·0%)	2460 (27·5%)	2200 (35·3%)
	Other forms of heart disease	42 431 (18·0%)	26 066 (13·7%)	16 365 (35·3%)	4678 (52·3%)	3694 (59·3%)
	Chronic kidney disease	15 908 (6·7%)	8345 (4·4%)	7563 (16·3%)	1941 (21·7%)	1892 (30·4%)
	Neoplasms	45 255 (19·1%)	34 362 (18·1%)	10 893 (23·5%)	2339 (26·1%)	1793 (28·8%)

Data are n (%) or mean (SD). Only characteristics with a prevalence higher than 5% in the whole population are displayed. Additional baseline characteristics are presented in the appendix (pp 25–27). ITU=intensive therapy unit.

We estimated the diagnostic incidence of the neurological and psychiatric outcomes of the primary cohort in the 6 months after a COVID-19 diagnosis. In the whole cohort, 33·62% (95% CI 33·17–34·07) of patients received a diagnosis (table 2). For the cohort subgroups, these estimates were 38·73% (37·87–39·60) for patients who were hospitalised, 46·42% (44·78–48·09) for those admitted to ITU, and 62·34% (60·14–64·55) for those diagnosed with encephalopathy. A similar, but more marked, increasing trend was observed for patients receiving their first recorded neurological or psychiatric diagnosis (table 2). Results according to sex, race, and age are shown in the appendix (p 28). The baseline characteristics of the COVID-19 cohort divided into those who did versus those who did not have a neurological or psychiatric outcome are also shown in the appendix (p 7).

**Table 2:** Major outcomes for the whole COVID-19 cohort, and for the non-hospitalisation, hospitalisation, ITU admission, and encephalopathy cohorts during the illness

	**All patients**	**Patients without hospitalisation**	**Patients with hospitalisation**	**Patients with ITU admission**	**Patients with encephalopathy**
Intracranial haemorrhage (any)	0·56% (0·50–0·63)	0·31% (0·25–0·39)	1·31% (1·14–1·52)	2·66% (2·24–3·16)	3·61% (2·97–4·39)
Intracranial haemorrhage (first)	0·28% (0·23–0·33)	0·14% (0·10–0·20)	0·63% (0·50–0·80)	1·05% (0·79–1·40)	1·19% (0·82–1·70)
Ischaemic stroke (any)	2·10% (1·97–2·23)	1·33% (1·22–1·46)	4·38% (4·05–4·74)	6·92% (6·17–7·76)	9·35% (8·23–10·62)
Ischaemic stroke (first)	0·76% (0·68–0·85)	0·43% (0·36–0·52)	1·60% (1·37–1·86)	2·82% (2·29–3·47)	3·28% (2·51–4·27)
Parkinsonism	0·11% (0·08–0·14)	0·07% (0·05–0·12)	0·20% (0·15–0·28)	0·26% (0·15–0·45)	0·46% (0·28–0·78)
Guillain-Barré syndrome	0·08% (0·06–0·11)	0·05% (0·03–0·07)	0·22% (0·15–0·32)	0·33% (0·21–0·54)	0·48% (0·20–1·14)
Nerve, nerve root, or plexus disorders	2·85% (2·69–3·03)	2·69% (2·51–2·89)	3·35% (3·02–3·72)	4·24% (3·58–5·03)	4·69% (3·81–5·77)
Myoneural junction or muscle disease	0·45% (0·40–0·52)	0·16% (0·12–0·20)	1·24% (1·05–1·46)	3·35% (2·76–4·05)	3·27% (2·54–4·21)
Encephalitis	0·10% (0·08–0·13)	0·05% (0·03–0·08)	0·24% (0·17–0·33)	0·35% (0·19–0·64)	0·64% (0·39–1·07)
Dementia	0·67% (0·59–0·75)	0·35% (0·29–0·43)	1·46% (1·26–1·71)	1·74% (1·31–2·30)	4·72% (3·80–5·85)
Mood, anxiety, or psychotic disorder (any)	23·98% (23·58–24·38)	23·59% (23·12–24·07)	24·50% (23·76–25·26)	27·78% (26·33–29·29)	36·25% (34·16–38·43)
Mood, anxiety, or psychotic disorder (first)	8·63% (8·28–8·98)	8·15% (7·75–8·57)	8·85% (8·22–9·52)	12·68% (11·28–14·24)	12·96% (11·13–15·07)
Mood disorder (any)	13·66% (13·35–13·99)	13·10% (12·73–13·47)	14·69% (14·09–15·32)	15·43% (14·27–16·68)	22·52% (20·71–24·47)
Mood disorder (first)	4·22% (3·99–4·47)	3·86% (3·60–4·14)	4·49% (4·05–4·99)	5·82% (4·86–6·97)	8·07% (6·56–9·90)
Anxiety disorder (any)	17·39% (17·04–17·74)	17·51% (17·09–17·93)	16·40% (15·76–17·06)	19·15% (17·90–20·48)	22·43% (20·65–24·34)
Anxiety disorder (first)	7·11% (6·82–7·41)	6·81% (6·47–7·16)	6·91% (6·38–7·47)	9·79% (8·65–11·06)	9·24% (7·70–11·07)
Psychotic disorder (any)	1·40% (1·30–1·51)	0·93% (0·83–1·04)	2·89% (2·62–3·18)	2·77% (2·31–3·33)	7·00% (6·01–8·14)
Psychotic disorder (first)	0·42% (0·36–0·49)	0·25% (0·19–0·33)	0·89% (0·72–1·09)	0·70% (0·46–1·06)	2·12% (1·53–2·94)
Substance use disorder (any)	6·58% (6·36–6·80)	5·87% (5·63–6·13)	8·56% (8·10–9·04)	10·14% (9·25–11·10)	11·85% (10·55–13·31)
Substance use disorder (first)	1·92% (1·77–2·07)	1·74% (1·58–1·91)	2·09% (1·82–2·40)	3·15% (2·60–3·82)	2·58% (1·91–3·47)
Insomnia (any)	5·42% (5·20–5·64)	5·16% (4·91–5·42)	5·95% (5·53–6·39)	7·50% (6·66–8·44)	9·82% (8·57–11·24)
Insomnia (first)	2·53% (2·37–2·71)	2·23% (2·05–2·43)	3·14% (2·81–3·51)	4·24% (3·55–5·07)	5·05% (4·10–6·20)
Any outcome	33·62% (33·17–34·07)	31·74% (31·22–32·27)	38·73% (37·87–39·60)	46·42% (44·78–48·09)	62·34% (60·14–64·55)
Any first outcome	12·84% (12·36–13·33)	11·51% (10·98–12·07)	15·29% (14·32–16·33)	25·79% (23·50–28·25)	31·13% (27·29–35·36)

Data are percentage at 6 months (95% CI). Additional outcomes are presented in the appendix (pp 27–28). ITU=intensive therapy unit.

**Figure 1:**
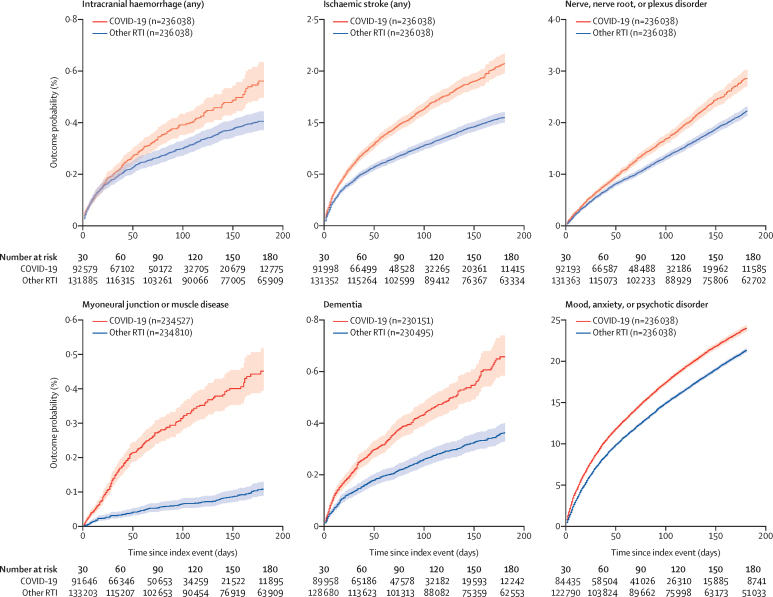
Kaplan-Meier estimates for the incidence of major outcomes after COVID-19 compared with other RTIs Shaded areas are 95% CIs. For incidences of first diagnoses, the number in brackets corresponds to all patients who did not have the outcome before the follow-up period. For diagnostic subcategories, see appendix (pp 8–10). RTI=respiratory tract infection.

We assessed the probability of the major neurological and psychiatric outcomes in patients diagnosed with COVID-19 compared with the matched cohorts diagnosed with other respiratory tract infections and with influenza (table 3; figure 1, appendix pp 8–10). Most diagnostic categories were more common in patients who had COVID-19 than in those who had influenza (HR 1·44, 95% CI 1·40–1·47 for any diagnosis; 1·78, 1·68–1·89 for any first diagnosis) and those who had other respiratory tract infections (1·16, 1·14–1·17 for any diagnosis; 1·32, 1·27–1·36 for any first diagnosis). Hazard rates were also higher in patients who were admitted to ITU than in those who were not (1·58, 1·50–1·67 for any diagnosis; 2·87, 2·45–3·35 for any first diagnosis). HRs were significantly greater than 1 for all diagnoses for patients who had COVID-19 compared with those who had influenza, except for parkinsonism and Guillain-Barré syndrome, and significantly greater than 1 for all diagnoses compared with patients who had respiratory tract infections (table 3). Similar results were observed when patients who had COVID-19 were compared with those who had one of the four other index events (appendix pp 11–14, 34), except when an outcome had a predicted relationship with the comparator condition (eg, intracranial haemorrhage was more common in association with fracture of a large bone). HRs for diagnostic subcategories are presented in the appendix (p 33).

**Table 3:** HRs for the major outcomes in patients after COVID-19 compared with those after influenza and other RTIs

	COVID-19 *vs* influenza (N=105 579)*		COVID-19 *vs* other RTI (N=236 038)*	
	HR (95% CI)	p value	HR (95% CI)	p value
Intracranial haemorrhage (any)	2·44 (1·89–3·16)	<0·0001	1·26 (1·11–1·43)	0·0003
Intracranial haemorrhage (first)	2·53 (1·68–3·79)	<0·0001	1·56 (1·27–1·92)	<0·0001
Ischaemic stroke (any)	1·62 (1·43–1·83)	<0·0001	1·45 (1·36–1·55)	<0·0001
Ischaemic stroke (first)	1·97 (1·57–2·47)	<0·0001	1·63 (1·44–1·85)	<0·0001
Parkinsonism	1·42 (0·75–2·67)	0·19	1·45 (1·05–2·00)	0·020
Guillain-Barré syndrome	1·21 (0·72–2·04)	0·41	2·06 (1·43–2·96)	<0·0001
Nerve, nerve root, or plexus disorders	1·64 (1·50–1·81)	<0·0001	1·27 (1·19–1·35)	<0·0001
Myoneural junction or muscle disease	5·28 (3·71–7·53)	<0·0001	4·52 (3·65–5·59)	<0·0001
Encephalitis	1·70 (1·04–2·78)	0·028	1·41 (1·03–1·92)	0·028
Dementia	2·33 (1·77–3·07)	<0·0001	1·71 (1·50–1·95)	<0·0001
Mood, anxiety, or psychotic disorder (any)	1·46 (1·43–1·50)	<0·0001	1·20 (1·18–1·23)	<0·0001
Mood, anxiety, or psychotic disorder (first)	1·81 (1·69–1·94)	<0·0001	1·48 (1·42–1·55)	<0·0001
Mood disorder (any)	1·47 (1·42–1·53)	<0·0001	1·23 (1·20–1·26)	<0·0001
Mood disorder (first)	1·79 (1·64–1·95)	<0·0001	1·41 (1·33–1·50)	<0·0001
Anxiety disorder (any)	1·45 (1·40–1·49)	<0·0001	1·17 (1·15–1·20)	<0·0001
Anxiety disorder (first)	1·78 (1·66–1·91)	<0·0001	1·48 (1·42–1·55)	<0·0001
Psychotic disorder (any)	2·03 (1·78–2·31)	<0·0001	1·66 (1·53–1·81)	<0·0001
Psychotic disorder (first)	2·16 (1·62–2·88)	<0·0001	1·82 (1·53–2·16)	<0·0001
Substance use disorder (any)	1·27 (1·22–1·33)	<0·0001	1·09 (1·05–1·12)	<0·0001
Substance use disorder (first)	1·22 (1·09–1·37)	0·0006	0·92 (0·86–0·99)	0·033
Insomnia (any)	1·48 (1·38–1·57)	<0·0001	1·15 (1·10–1·20)	<0·0001
Insomnia (first)	1·92 (1·72–2·15)	<0·0001	1·43 (1·34–1·54)	<0·0001
Any outcome	1·44 (1·40–1·47)	<0·0001	1·16 (1·14–1·17)	<0·0001
Any first outcome	1·78 (1·68–1·89)	<0·0001	1·32 (1·27–1·36)	<0·0001

Additional details on cohort characteristics and diagnostic subcategories are presented in the appendix (pp 29–33). HR=hazard ratio. RTI=respiratory tract infection.

* Matched cohorts.

There were no violations of the proportional hazards assumption for most of the neurological outcomes over the 6 months of follow-up (appendix pp 15, 35). The only exception was for intracranial haemorrhage and ischaemic stroke in patients who had COVID-19 when compared with patients who had other respiratory tract infections (p=0·012 for intracranial haemorrhage and p=0·032 for ischaemic stroke). For the overall psychiatric disorder category (ICD-10 F20–48), the HR did vary with time, declining but remaining significantly higher than 1, indicating that the risk was attenuated but maintained 6 months after COVID-19 diagnosis (appendix p 9). HRs for COVID-19 diagnosis compared with the additional four index events showed more variation with time, partly reflecting the natural history of the comparator condition (appendix, pp 16–19, 36).

**Figure 2:**
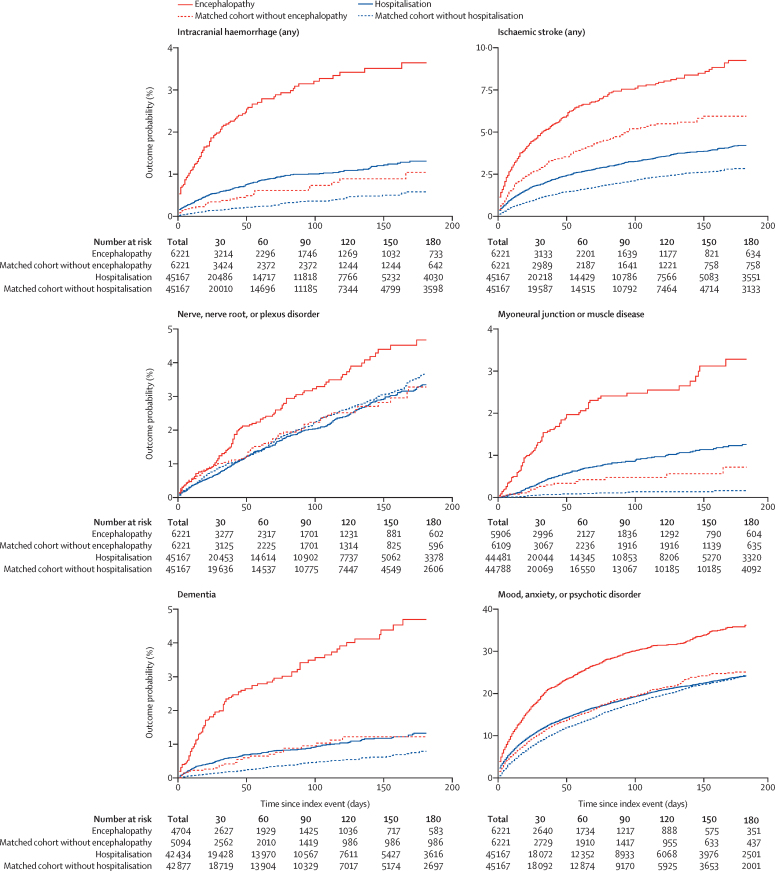
Kaplan-Meier estimates for the incidence of major outcomes after COVID-19 comparing patients requiring hospitalisation with matched patients not requiring hospitalisation, and comparing those who had encephalopathy with matched patients who did not have encephalopathy 95% CIs are omitted for clarity but are shown in the appendix (p 23). For incidences of first diagnoses, the total number corresponds to all patients who did not have the outcome before the follow-up period. The equivalent figure showing the comparison between patients with intensive therapy unit admission versus those without is presented in the appendix (p 22).

We explored the effect of COVID-19 severity in four ways. First, we restricted analyses to matched cohorts of patients who had not required hospitalisation (matched baseline characteristics in the appendix, pp 37–40). HRs remained significantly greater than 1 in this subgroup, with an overall HR for any diagnosis of 1·47 (95% CI 1·44–1·51) for patients who had COVID-19 compared with patients who had influenza, and 1·16 (1·14–1·17) compared with those who had other respiratory tract infections (table 4, appendix pp 20–21). For a first diagnosis, the HRs were 1·83 (1·71–1·96) versus patients who had influenza and 1·28 (1·23–1·33) versus those who had other respiratory tract infections. Second, we calculated HRs for the matched cohorts of patients with COVID-19 requiring hospitalisation versus those who did not require hospitalisation (44 927 matched patients; matched baseline characteristics are presented in the appendix, pp 41–42). This comparison showed greater hazard rates for all outcomes in the hospitalised group than in the non-hospitalised group, except for nerve, nerve root, or plexus disorders (table 5, figure 2), with an overall HR of 1·33 (1·29–1·37) for any diagnosis and 1·70 (1·56–1·86) for any first diagnosis. Third, we calculated HRs for the matched cohorts of patients with COVID-19 requiring ITU admission versus those not requiring ITU admission (8942 patients; matched baseline characteristics presented in the appendix, pp 43–44), with a HR of 1·58 (1·50–1·67) for any diagnosis and 2·87 (2·45–3·35) for any first diagnosis (table 5, appendix p 22). Fourth, we calculated HRs for the matched cohorts of patients with COVID-19 who had encephalopathy diagnosed during acute illness versus those who did not (6221 patients; matched baseline characteristics presented in the appendix, pp 45–46). HRs for all diagnoses were greater for the group who had encephalopathy than for the matched cohort who did not, with an overall HR of 1·85 (1·73–1·98) for any diagnosis and 3·19 (2·54–4·00) for any first diagnosis (table 5, figure 2).

**Table 4:** HRs for the major outcomes in patients without hospitalisation after COVID-19 compared with those after influenza or other RTIs

	COVID-19 *vs* influenza in patients without hospitalisation (N=96 803)*		COVID-19 *vs* other RTI in patients without hospitalisation (N=183 731)*	
	HR (95% CI)	p value	HR (95% CI)	p value
Intracranial haemorrhage (any)	1·87 (1·25–2·78)	0·0013	1·38 (1·11–1·73)	0·0034
Intracranial haemorrhage (first)	1·66 (0·88–3·14)	0·082	1·63 (1·11–2·40)	0·010
Ischaemic stroke (any)	1·80 (1·54–2·10)	<0·0001	1·61 (1·45–1·78)	<0·0001
Ischaemic stroke (first)	1·71 (1·26–2·33)	0·0003	1·69 (1·38–2·08)	<0·0001
Parkinsonism	2·22 (0·98–5·06)	0·028	1·20 (0·73–1·96)	0·42
Guillain-Barré syndrome	0·90 (0·44–1·84)	0·99	1·44 (0·85–2·45)	0·10
Nerve, nerve root, or plexus disorders	1·69 (1·53–1·88)	<0·0001	1·23 (1·15–1·33)	<0·0001
Myoneural junction or muscle disease	3·46 (2·11–5·67)	<0·0001	2·69 (1·91–3·79)	<0·0001
Encephalitis	1·77 (0·86–3·66)	0·095	2·29 (1·28–4·10)	0·0046
Dementia	1·88 (1·27–2·77)	0·0008	1·95 (1·55–2·45)	<0·0001
Mood, anxiety, or psychotic disorder (any)	1·49 (1·45–1·54)	<0·0001	1·18 (1·15–1·21)	<0·0001
Mood, anxiety, or psychotic disorder (first)	1·85 (1·72–1·99)	<0·0001	1·40 (1·32–1·48)	<0·0001
Mood disorder (any)	1·49 (1·43–1·55)	<0·0001	1·22 (1·19–1·26)	<0·0001
Mood disorder (first)	1·78 (1·61–1·96)	<0·0001	1·37 (1·27–1·47)	<0·0001
Anxiety disorder (any)	1·48 (1·43–1·54)	<0·0001	1·16 (1·13–1·19)	<0·0001
Anxiety disorder (first)	1·80 (1·67–1·94)	<0·0001	1·37 (1·30–1·45)	<0·0001
Psychotic disorder (any)	1·93 (1·63–2·28)	<0·0001	1·44 (1·27–1·62)	<0·0001
Psychotic disorder (first)	2·27 (1·56–3·30)	<0·0001	1·49 (1·15–1·93)	0·0016
Substance use disorder (any)	1·26 (1·19–1·33)	<0·0001	1·11 (1·07–1·17)	<0·0001
Substance use disorder (first)	1·21 (1·05–1·38)	0·0054	0·89 (0·81–0·97)	0·013
Insomnia (any)	1·52 (1·42–1·63)	<0·0001	1·18 (1·12–1·24)	<0·0001
Insomnia (first)	2·06 (1·82–2·33)	<0·0001	1·51 (1·38–1·66)	<0·0001
Any outcome	1·47 (1·44–1·51)	<0·0001	1·16 (1·14–1·17)	<0·0001
Any first outcome	1·83 (1·71–1·96)	<0·0001	1·28 (1·23–1·33)	<0·0001

Details on cohort characteristics are presented in the appendix (pp 37–40). HR=hazard ratio. RTI=respiratory tract infection.

* Matched cohorts.

**Table 5:** HRs for the major outcomes after COVID-19 for patients with *vs* those without hospitalisation, patients with *vs* without ITU admission, and patients with *vs* without encephalopathy

	**COVID-19 with *vs* without hospitalisation (N=45 167)**		**COVID-19 with *vs* without ITU admission (N=8942)**		**COVID-19 with *vs* without encephalopathy (N=6221)**	
	HR (95% CI)	p value	HR (95% CI)	p value	HR (95% CI)	p value
Intracranial haemorrhage (any)	3·09 (2·43–3·94)	<0·0001	5·06 (3·43–7·47)	<0·0001	4·73 (3·15–7·11)	<0·0001
Intracranial haemorrhage (first)	3·75 (2·49–5·64)	<0·0001	5·12 (2·68–9·77)	<0·0001	5·00 (2·33–10·70)	<0·0001
Ischaemic stroke (any)	1·65 (1·48–1·85)	<0·0001	1·93 (1·62–2·31)	<0·0001	1·65 (1·38–1·97)	<0·0001
Ischaemic stroke (first)	2·82 (2·22–3·57)	<0·0001	3·51 (2·39–5·15)	<0·0001	3·39 (2·17–5·29)	<0·0001
Parkinsonism	2·63 (1·45–4·77)	0·0016	3·90 (1·29–11·79)	0·024	1·64 (0·75–3·58)	0·24
Guillain-Barré syndrome	2·94 (1·60–5·42)	0·00094	11·01 (2·55–47·61)	0·0007	2·27 (0·76–6·73)	0·24
Nerve, nerve root, or plexus disorders	0·94 (0·83–1·06)	0·29	1·16 (0·92–1·45)	0·21	1·41 (1·07–1·87)	0·018
Myoneural junction or muscle disease	7·76 (5·15–11·69)	<0·0001	11·53 (6·38–20·83)	<0·0001	5·40 (3·21–9·07)	<0·0001
Encephalitis	3·26 (1·75–6·06)	0·0002	1·78 (0·75–4·20)	0·22	9·98 (2·98–33·43)	<0·0001
Dementia	2·28 (1·80–2·88)	<0·0001	1·66 (1·12–2·46)	0·018	4·25 (2·79–6·47)	<0·0001
Mood, anxiety, or psychotic disorder (any)	1·23 (1·18–1·28)	<0·0001	1·34 (1·24–1·46)	<0·0001	1·73 (1·58–1·90)	<0·0001
Mood, anxiety, or psychotic disorder (first)	1·55 (1·40–1·71)	<0·0001	2·27 (1·87–2·74)	<0·0001	2·28 (1·80–2·89)	<0·0001
Mood disorder (any)	1·21 (1·15–1·28)	<0·0001	1·15 (1·03–1·27)	0·010	1·51 (1·35–1·70)	<0·0001
Mood disorder (first)	1·53 (1·33–1·75)	<0·0001	2·06 (1·57–2·71)	<0·0001	2·09 (1·55–2·80)	<0·0001
Anxiety disorder (any)	1·16 (1·10–1·22)	<0·0001	1·39 (1·26–1·53)	<0·0001	1·64 (1·45–1·84)	<0·0001
Anxiety disorder (first)	1·49 (1·34–1·65)	<0·0001	2·22 (1·82–2·71)	<0·0001	1·91 (1·48–2·45)	<0·0001
Psychotic disorder (any)	2·22 (1·92–2·57)	<0·0001	1·48 (1·14–1·92)	0·0028	3·84 (2·90–5·10)	<0·0001
Psychotic disorder (first)	2·77 (1·99–3·85)	<0·0001	1·77 (0·98–3·20)	0·072	5·62 (2·93–10·77)	<0·0001
Substance use disorder (any)	1·53 (1·42–1·64)	<0·0001	1·62 (1·41–1·85)	<0·0001	1·45 (1·24–1·70)	<0·0001
Substance use disorder (first)	1·68 (1·40–2·01)	<0·0001	2·53 (1·83–3·50)	<0·0001	2·03 (1·32–3·11)	0·0015
Insomnia (any)	1·08 (0·99–1·18)	0·088	1·40 (1·19–1·66)	<0·0001	1·73 (1·42–2·11)	<0·0001
Insomnia (first)	1·49 (1·28–1·74)	<0·0001	1·93 (1·46–2·55)	<0·0001	3·44 (2·35–5·04)	<0·0001
Any outcome	1·33 (1·29–1·37)	<0·0001	1·58 (1·50–1·67)	<0·0001	1·85 (1·73–1·98)	<0·0001
Any first outcome	1·70 (1·56–1·86)	<0·0001	2·87 (2·45–3·35)	<0·0001	3·19 (2·54–4·00)	<0·0001

Details on cohort characteristics are presented in the appendix (pp 41–46). HR=hazard ratio. ITU=intensive therapy unit.

* Matched cohorts.

We inspected other factors that might influence the findings. The results regarding hospitalisation, ITU admission, or encephalopathy (which we had defined as occurring up to 14 days after diagnosis) could be confounded by admissions due to an early complication of COVID-19 rather than to COVID-19 itself. This was explored by excluding outcomes during this period, with the findings remaining similar, albeit with many HRs being reduced (appendix pp 47–49). Additionally, COVID-19 survivors had fewer health-care visits during the 6-month period compared with the other cohorts (appendix p 50). Hence the higher incidence of many diagnoses was not simply due to having had more diagnostic opportunities.

The increased rates of neurological and psychiatric sequelae were robust in all three sensitivity analyses: when patients who had died by the time of the analysis were included (appendix p 51), when the COVID-19 diagnosis was confirmed by use of an RNA or antigen test (appendix p 52), and when the sequelae were compared with those observed in patients who had influenza in 2019 or 2018 (appendix pp 53).

## Discussion

Various adverse neurological and psychiatric outcomes occurring after COVID-19 have been predicted and reported.^1^, ^2^, ^3^, ^4^, ^5^, ^14^ The data presented in this study, from a large electronic health records network, support these predictions and provide estimates of the incidence and risk of these outcomes in patients who had COVID-19 compared with matched cohorts of patients with other health conditions occurring contemporaneously with the COVID-19 pandemic (Table 2, Table 3, figure 1).

The severity of COVID-19 had a clear effect on subsequent neurological diagnoses (Table 4, Table 5, figure 2). Overall, COVID-19 was associated with increased risk of neurological and psychiatric outcomes, but the incidences and HRs of these were greater in patients who had required hospitalisation, and markedly so in those who had required ITU admission or had developed encephalopathy, even after extensive propensity score matching for other factors (eg, age or previous cerebrovascular disease). Potential mechanisms for this association include viral invasion of the CNS,^10^, ^11^ hypercoagulable states,^22^ and neural effects of the immune response.^9^ However, the incidence and relative risk of neurological and psychiatric diagnoses were also increased even in patients with COVID-19 who did not require hospitalisation.

Some specific neurological diagnoses merit individual mention. Consistent with several other reports,^23^, ^24^ the risk of cerebrovascular events (ischaemic stroke and intracranial haemorrhage) was elevated after COVID-19, with the incidence of ischaemic stroke rising to almost one in ten (or three in 100 for a first stroke) in patients with encephalopathy. A similarly increased risk of stroke in patients who had COVID-19 compared with those who had influenza has been reported.^25^ Our previous study reported preliminary evidence for an association between COVID-19 and dementia.^14^ The data in this study support this association. Although the estimated incidence was modest in the whole COVID-19 cohort (table 2), 2·66% of patients older than 65 years (appendix p 28) and 4·72% who had encephalopathy (table 2), received a first diagnosis of dementia within 6 months of having COVID-19. The associations between COVID-19 and cerebrovascular and neurodegenerative diagnoses are concerning, and information about the severity and subsequent course of these diseases is required.

Whether COVID-19 is associated with Guillain-Barré syndrome remains unclear;^26^ our data were also equivocal, with HRs increased with COVID-19 compared with other respiratory tract infections but not with influenza (table 3), and increased compared with three of the four other index health events (appendix p 34). Concerns have also been raised about post-COVID-19 parkinsonian syndromes, driven by the encephalitis lethargica epidemic that followed the 1918 influenza pandemic.^27^ Our data provide some support for this possibility, although the incidence was low and not all HRs were significant. Parkinsonism might be a delayed outcome, in which case a clearer signal might emerge with a longer follow-up.

The findings regarding anxiety and mood disorders were broadly consistent with 3-month outcome data from a study done in a smaller number of cases than our cohort, using the same network,^14^ and showed that the HR remained elevated, although decreasing, at the 6-month period. Unlike the earlier study, and in line with previous suggestions,^28^ we also observed a significantly increased risk of psychotic disorders, probably reflecting the larger sample size and longer duration of follow-up reported here. Substance use disorders and insomnia were also more common in COVID-19 survivors than in those who had influenza or other respiratory tract infections (except for the incidence of a first diagnosis of substance use disorder after COVID-19 compared with other respiratory tract infections). Therefore, as with the neurological outcomes, the psychiatric sequelae of COVID-19 appear widespread and to persist up to, and probably beyond, 6 months. Compared with neurological disorders, common psychiatric disorders (mood and anxiety disorders) showed a weaker relationship with the markers of COVID-19 severity in terms of incidence (table 2) or HRs (table 5). This might indicate that their occurrence reflects, at least partly, the psychological and other implications of a COVID-19 diagnosis rather than being a direct manifestation of the illness.

HRs for most neurological outcomes were constant, and hence the risks associated with COVID-19 persisted up to the 6-month timepoint. Longer-term studies are needed to ascertain the duration of risk and the trajectory for individual diagnoses.

Our findings are robust given the sample size, the propensity score matching, and the results of the sensitivity and secondary analyses. Nevertheless, they have weaknesses inherent to an electronic health records study,^29^ such as the unknown completeness of records, no validation of diagnoses, and sparse information on socioeconomic and lifestyle factors. These issues primarily affect the incidence estimates, but the choice of cohorts against which to compare COVID-19 outcomes influenced the magnitude of the HRs (table 3, appendix p 34). The analyses regarding encephalopathy (delirium and related conditions) deserve a note of caution. Even among patients who were hospitalised, only about 11% received this diagnosis, whereas much higher rates would be expected.^18^, ^30^ Under-recording of delirium during acute illness is well known and probably means that the diagnosed cases had prominent or sustained features; as such, results for this group should not be generalised to all patients with COVID-19 who experience delirium. We also note that encephalopathy is not just a severity marker but a diagnosis in itself, which might predispose to, or be an early sign of, other neuropsychiatric or neurodegenerative outcomes observed during follow-up. The timing of index events was such that most infections with influenza and many of the other respiratory tract infections occurred earlier on during the pandemic, whereas the incidence of COVID-19 diagnoses increased over time (appendix p 24). The effect of these timing differences on observed rates of sequelae is unclear but, if anything, they are likely to make the HRs an underestimate because COVID-19 cases were diagnosed at a time when all other diagnoses were made at a lower rate in the population (appendix p 24). Some patients in the comparison cohorts are likely to have had undiagnosed COVID-19; this would also tend to make our HRs an underestimate. Finally, a study of this kind can only show associations; efforts to identify mechanisms and assess causality will require prospective cohort studies and additional study designs.

In summary, the present data show that COVID-19 is followed by significant rates of neurological and psychiatric diagnoses over the subsequent 6 months. Services need to be configured, and resourced, to deal with this anticipated need.

For the **TriNetX Analytics Network** see www.trinetx.com

For the **study data** see https://osf.io/7tzvy

## Data sharing

The TriNetX system returned the results of these analyses as .csv files, which were downloaded and archived. Data presented in this paper can be freely accessed online. Additionally, TriNetX will grant access to researchers if they have a specific concern (through a third-party agreement option).

## Declaration of interests

SL is an employee of TriNetX. All other authors declare no competing interests.
