# Supplementary material for: 6-month neurological and psychiatric outcomes in 236 379 survivors of COVID-19: a retrospective cohort study using electronic health records
Source: Lancet Psychiatry. 2021 May;8(5):416–27. doi: 10.1016/S2215-0366(21)00084-5 (PMC8023694; doi:10.1016/S2215-0366(21)00084-5)
Supplement: Supplementary appendix [file mmc1.pdf]

THE LANCET
Psychiatry

Supplementary appendix

This appendix formed part of the original submission and has been peer reviewed. We post it as supplied by the authors.

Supplement to: Taquet M, Geddes JR, Husain M, Luciano S, Harrison PJ. 6-month neurological and psychiatric outcomes in 236 379 survivors of COVID-19: a retrospective cohort study using electronic health records. *Lancet Psychiatry* 2021; published online Apr 6. http://dx.doi.org/10.1016/S2215-0366(21)00084-5.

Supplementary methods

TriNetX network

This section provides an expanded version of our previous description of the network[1].

Legal and ethical status
TriNetX's Analytics network is compliant with the Health Insurance Portability and Accountability Act (HIPAA), the US federal law which protects the privacy and security of healthcare data. TriNetX is certified to the ISO 27001:2013 standard and maintains an Information Security Management System (ISMS) to ensure the protection of the healthcare data it has access to and to meet the requirements of the HIPAA Security Rule. Any data displayed on the TriNetX Platform in aggregate form, or any patient level data provided in a data set generated by the TriNetX Platform, only contains de-identified data as per the de-identification standard defined in Section §164.514(a) of the HIPAA Privacy Rule. The process by which the data is de-identified is attested to through a formal determination by a qualified expert as defined in Section §164.514(b)(1) of the HIPAA Privacy Rule. This formal determination by a qualified expert, refreshed in December 2020, supersedes the need for TriNetX's previous waiver from the Western Institutional Review Board (IRB). The network contains data that are provided by participating Health Care Organizations (HCOs), each of which represents and warrants that it has all necessary rights, consents, approvals and authority to provide the data to TriNetX under a Business Associate Agreement (BAA), so long as their name remains anonymous as a data source and their data are utilized for research purposes. The data shared through the TriNetX Platform are attenuated to ensure that they do not include sufficient information to facilitate the determination of which HCO contributed which specific information about a patient.

Acquisition of data, quality control, and other procedures
The data are stored onboard a TriNetX appliance – a physical server residing at the institution's data centre or a virtual hosted appliance. The TriNetX platform is a fleet of these appliances connected into a federated network able to broadcast queries to each appliance. Results are subsequently collected and aggregated.

Once the data are sent to the network, they are mapped to a standard and controlled set of clinical terminologies and undergo a data quality assessment including 'data cleaning' that rejects records which do not meet the TriNetX quality standards. HIPAA compliance of the clinical patient data is achieved using de-identification. Different data modalities are available in the network. They include demographics (coded to HL7 version 3 administrative standards), diagnoses (represented by ICD-10-CM codes), procedures (coded in ICD-10-PCS or CPT), and measurements (coded to LOINC). While extensive information is provided about patients' diagnoses and procedures, other variables (such as socioeconomic and lifetime factors are not comprehensively represented).

The data from a typical HCO generally go back around 7 years, with some going back 13 years. The data are continuously updated. HCOs update their data at various times, with most refreshing every 1, 2, or 4 weeks.

The data come primarily (>93%) from HCOs in the USA, with the remainder coming from India, Australia, Malaysia, Taiwan, Spain, UK, and Bulgaria. As noted above, to comply with legal frameworks and ethical guidelines guarding against data re-identification, the identity of participating HCOs and their individual contribution to each dataset are not disclosed to researchers.

Data quality assessment followed a standardised strategy wherein the data are reviewed for conformance (adherence to specified standards and formats), completeness (quantifying data presence or absence) and plausibility (believability of the data from a clinical perspective). There are pre-defined metrics for each of the above assessment categories. Results for these metrics are visualised and reviewed for each new site that joins the network as well as on an ongoing basis. Any identified issue is communicated to the data provider and resolved before continuing data collection.

The basic formatting of contributed data is also checked (e.g. to ensure that dates are properly represented). Records are checked against a list of required fields (e.g., patient identifier) and rejects those records for which the required information is missing. Referential integrity checking is done to ensure that data spanning multiple database tables can be successfully joined together. As the data are refreshed, changes in volume of data over

time is monitored to ensure data validity. At least one non-demographic fact for each patient is required for them to be counted in the dataset. Patient records with only demographics information are discarded.

The software also undergoes quality control. The engineers testing the software are independent from the engineers developing it. Each test code is checked by two independent testing engineers. Each piece of software is tested extensively against a range of synthetic data (i.e. generated for the purpose of testing) for which the expected output is established independently. If the software fails to return this output, then the software is deemed to have failed the test and is examined and modified accordingly. For statistical software (including that used for propensity score matching, for Kaplan-Meier analysis, etc), an additional quality control step is implemented. Two independent codes are written in two different programming languages (typically R and python) and the statistical results are compared. If discrepancies are identified, then the codes are deemed to have failed the test and are examined and modified accordingly. All the code is reviewed independently by another engineer.

The test strategy follows three levels of granularity:

1. Unit tests: These test specific blocks, or units, of code that perform specific actions (e.g. querying the database).
2. Integration tests: These ensure that different components are working together correctly.
3. End-to-end tests: These tests run the entire system and check the final output.

Some comments on advantages and disadvantages of EHR data

The advantage of EHR data, like those in TriNetX, over insurance claim data is that both insured and uninsured patients are included. An advantage of EHR data over survey data is that they represent the diagnostic rates in the population presenting to healthcare facilities. This provides an accurate account of the burden of specific diagnoses on healthcare systems. The downside of relying on diagnoses is that they obviously do not account for undiagnosed patients who might be suffering from the illness but did not seek medical attention (or in whom the diagnosis was missed). A general limitation of EHR data is that a patient may be seen in different HCOs for different parts of their care and if one HCO is not part of the federated network then part of their medical records may not be available. Using a network of HCOs (rather than a single HCO) limits this possibility but does not fully remove it. Finally, historical data before the start of EHRs (or the addition of an HCO to the network) may be incomplete.

Definition of cohorts

The two control cohorts used consisted of patients with a diagnosis of influenza and patients with another respiratory tract infection. Specifically, patients with influenza were those who had any of the following diagnoses:

- J09: Influenza due to certain identified influenza viruses
- J10: Influenza due to other identified influenza virus
- J11: Influenza due to unidentified influenza virus.

Patients with another respiratory tract infection were those with any of the following diagnosis:

- J00: Acute nasopharyngitis
- J01: Acute sinusitis
- J02: Acute pharyngitis
- J03: Acute tonsillitis
- J04: Acute laryngitis and tracheitis
- J05: Acute obstructive laryngitis [croup] and epiglottitis
- J06: Acute upper respiratory infections of multiple and unspecified sites
- J09: Influenza due to certain identified influenza viruses
- J10: Influenza due to other identified influenza virus
- J11: Influenza due to unidentified influenza virus
- J12: Viral pneumonia, not elsewhere classified
- J13: Pneumonia due to Streptococcus pneumoniae
- J14: Pneumonia due to Hemophilus influenzae
- J15: Bacterial pneumonia, not elsewhere classified
- J16: Pneumonia due to other infectious organisms, not elsewhere classified
- J17: Pneumonia in diseases classified elsewhere

- J18: Pneumonia, unspecified organism
- J20: Acute bronchitis
- J21: Acute bronchiolitis
- J22: Unspecified acute lower respiratory infection

Because some patients with the control index event might have had COVID-19 at a different point in time, we excluded from the control cohorts all those who had COVID-19 at any point in time. To avoid any contamination between cohorts, COVID-19 as an exclusion criterion was defined in the broader sense to be all patients with a confirmed diagnosis of COVID-19 (ICD-10 code U07.1) but also patients with an unconfirmed COVID-19 diagnosis (U07.2), a recorded positive PCR test for COVID-19, or any of the following recorded on or after January 20, 2020: Pneumonia due to SARS-associated coronavirus (J12.81), Other coronavirus as the cause of disease classified elsewhere (B97.29), or Coronavirus infection unspecified (B34.2). Inclusion of the latter three diagnostic codes captures patients who receive a COVID-19 diagnosis in the early stage of the pandemic when the ICD code for COVID-19 (U07) was not yet defined. Specifically, the following codes were excluded from the control cohorts if they occurred on or after January 20, 2020:

- U07.1: COVID-19, virus identified
- U07.2: COVID-19, virus not identified
- J12.81: Pneumonia due to SARS-associated coronavirus
- B97.29: Other coronavirus as the cause of disease classified elsewhere
- B34.2: Coronavirus infection, unspecified
- Positive SARS-CoV-2 RNA in Respiratory specimen
- Positive SARS-CoV-2 RNA in Unspecified specimen
- Positive SARS-CoV-2 N gene in Respiratory specimen
- Positive SARS-CoV-2 N gene in Unspecified specimen
- Positive SARS-CoV-2 RdRp gene in Respiratory specimen
- Positive SARS-CoV-2 E gene in Respiratory specimen
- Positive SARS-CoV-2 E gene in Unspecified specimen
- Positive SARS-CoV-2 RNA panel in Respiratory specimen
- Positive SARS-CoV-2 RNA panel in Unspecified specimen
- Positive SARS-CoV-2 RNA in Nasopharynx
- Positive SARS coronavirus 2 and related RNA
- Positive SARS-related coronavirus RNA in Respiratory specimen
- Positive SARS coronavirus 2 ORF1ab in Respiratory specimen

The duration of follow-up of the patients depended on when they had the index event. Patients who had the index events more than 6 months before the date of the analysis (December 13, 2020) had 6 months of follow-up. The other patients were followed up until December 13, 2020. The Kaplan-Meier estimator accommodates differences in duration of follow-up by means of censoring.

Definition of covariates

To reduce the effect of confounding on associations between a diagnosis of COVID-19 and a subsequent neurologic or psychiatric diagnosis, cohorts were matched for established or suspected risk factors for COVID-19[2–5] and for established risk factors for COVID-19 death[6] (taken to be risk factors of a more severe COVID-19 illness). The following confounding factors were therefore included (with ICD-10/CDC codes in brackets):

1) **Age** at the time of diagnosis
2) **Sex** coded as female, male, or other.
3) **Race** encoded as 6 separate dichotomous variables: White (2106-3), Black or African American (2054-5), American Indian or Alaska Native (1002-5), Asian (2028-9), Native Hawaiian or Other Pacific Islander (2076-8), or Unknown Race (2131-1)
4) **Ethnicity** encoded as Hispanic or Latino (2135-2), Not Hispanic or Latino (2186-5), or Unknown Ethnicity
5) **Socioeconomic deprivation** encoded as the ICD-10 code for Problems related to housing and economic circumstances (Z59)
6) **Obesity** encoded as one dichotomous variable and one categorical variable: Overweight and obesity (E66) and body mass index (categorised into < 25 kg/m^2, 25-30 kg/m^2, ≥ 30 kg/m^2 which are the WHO thresholds for not obese, pre-obese, and obesity).

7) **Hypertension** encoded as 2 dichotomous and 2 categorical variables: Hypertensive diseases (I10-I16), the now deprecated version that was used until 2018 Hypertension diseases (I10-I15), measurements of systolic blood pressure (categorised into < 140mmHg, 140-160mmHg, and ≥ 160mmHg), and diastolic blood pressure (categorised into < 90mmHg, 90-100mmHg, and ≥ 100mmHg). The blood pressure categories correspond to the absence of hypertension, stage 1 hypertension, and stage 2 (and over) hypertension as per the NICE guidelines.
8) **Diabetes mellitus** encoded as 2 dichotomous variables: Type 1 diabetes mellitus (E10) and Type 2 diabetes mellitus (E11)
9) **Chronic lower respiratory diseases** encoded by each sub-category of the corresponding ICD-10 group: Bronchitis, not specified as acute or chronic (J40), Simple and mucopurulent chronic bronchitis (J41), Unspecified chronic bronchitis (J42), Emphysema (J43), Other chronic obstructive pulmonary disease (J44), Asthma (J45), Bronchiectasis (J47)
10) **Nicotine dependence** encoded as the corresponding ICD-10 diagnosis (F17.2)
11) **Substance use disorder** encoded as the ICD-10 code for mental and behavioral disorders due to psychoactive substance use (F10-F19)
12) **Heart diseases** encoded as 2 categorical variables: Ischaemic heart disease (I20-I25) and Other forms of heart disease (I30-I52)
13) **Chronic kidney disease** encoded as 2 dichotomous variables: Chronic kidney disease (N18) and Hypertensive chronic kidney disease (I12)
14) **Chronic liver disease** encoded as 8 categorical variables: Alcoholic liver disease (K70), Hepatic failure, not elsewhere classified (K72), Chronic hepatitis, not elsewhere classified (K73), Fibrosis and cirrhosis of liver (K74), Fatty (change of) liver, not elsewhere classified (K76.0), Chronic passive congestion of liver (K76.1), Portal hypertension (K76.6), Other specified diseases of liver (K76.8)
15) **Stroke** encoded as the dichotomous variable Cerebral infarction (I63)
16) **Dementia** encoded as 6 dichotomous variables: Vascular dementia (F01), Dementia in other diseases classified elsewhere (F02), Unspecified dementia (F03), Alzheimer's disease (G30), Frontotemporal dementia (G31.0), and Dementia with Lewy bodies (G31.83)
17) **Cancer and haematological cancer in particular** encoded as 2 dichotomous variables: Neoplasms (C00-D49) and Malignant neoplasms of lymphoid, hematopoietic and related tissue (C81-C96)
18) **Organ transplant** encoded as 2 dichotomous variables: Renal Transplantation Procedures and Liver Transplantation Procedures
19) **Rheumatoid arthritis** encoded as 2 dichotomous variables: Rheumatoid arthritis with rheumatoid factor (M05) and Other rheumatoid arthritis (M06)
20) **Lupus** encoded as a dichotomous variable corresponding ICD-10 code (M32)
21) **Psoriasis** encoded as a dichotomous variable corresponding ICD-10 code (L40)
22) **Disorders involving an immune mechanism** encoded as a dichotomous variable "Certain disorders involving the immune mechanism" (D80-D89)

Each individual code was considered a confounding factor in and of itself so that matching was achieved for each of them individually. For instance, matching was achieved for each subcategory (and not just for the whole category) of chronic lower respiratory diseases. For variables representing diagnoses and socioeconomic deprivation, an individual was considered positive if the diagnostic was recorded at least once in their health record before the index event. For categorical variables representing measurements (i.e. BMI and blood pressures), all available measurements for all individuals were used and propensity score matching sought to define cohorts with similar numbers of measurements falling into each category.

Definition of outcomes

All outcomes were defined as a diagnosis recorded in the patient's electronic health record between 1 day and 180 days after the index event. As mentioned in the manuscript, for chronic illnesses, only *first* diagnoses were counted (i.e. patients with the diagnosis before the index event were excluded from the survival analysis). For diagnoses that can recur or relapse, we separately estimated the incidence of *first* diagnosis and the incidence of *any* diagnoses. For all other diagnoses, we estimated the incidence of *any* diagnoses.

Specifically the following ICD-10 codes (with the ICD-10 labels in brackets) were used to define outcomes:

1) Intracranial haemorrhage (first and any diagnosis): I60 (non-traumatic subarachnoid haemorrhage), I61 (non-traumatic intracerebral haemorrhage), and I62 (other and unspecified non-traumatic intracranial haemorrhage)

2) Ischaemic stroke (first and any diagnosis): I63 (cerebral infarction)
3) Parkinsonism (first diagnosis): G20 (Parkinson's disease) or G21 (Secondary parkinsonism)
4) Guillain-Barre syndrome (any diagnosis): G61.0 (Guillain-Barre syndrome)
5) Nerve/nerve root/plexus disorders (any diagnosis): G50-G59 (Nerve, nerve root and plexus disorders)
6) Myoneural junction/muscle disease (first diagnosis): G70-G73 (Diseases of myoneural junction and muscle) – these disorders are often called neuromuscular, but we use the ICD-10 term
7) Encephalitis (any diagnosis): G04 (Encephalitis, myelitis and encephalomyelitis), G05 (Encephalitis, myelitis and encephalomyelitis in diseases classified elsewhere), A86 (Unspecified viral encephalitis), or A85.8 (Other specified viral encephalitis)
8) Dementia (first diagnosis): F01 (Vascular dementia), F02 (Dementia in other diseases classified elsewhere), F03 (Unspecified dementia), G30 (Alzheimer's disease), G31.0 (Frontotemporal dementia), G31.83 (Dementia with Lewy bodies)
9) Mood/Anxiety/Psychotic disorder (first and any diagnosis): F20-F29 (Schizophrenia, schizotypal, delusional, and other non-mood psychotic disorders), F30-F39 (Mood disorders), F40-F48 (Anxiety, dissociative, stress-related, somatoform and other nonpsychotic mental disorders)
    a) Mood disorder (first and any diagnosis): F30-F39
    b) Anxiety disorder (first and any diagnosis): F40-F48
    c) Psychotic disorder (first and any diagnosis): F20-F29
10) Substance misuse (first and any diagnosis): F10-F19 (Mental and behavioral disorders due to psychoactive substance use)
11) Insomnia (first and any diagnosis): F51.0 (Insomnia not due to a substance or known physiological condition) or G47.0 (Insomnia)

In the definition of mood disorder and substance misuse, ICD-10 codes representing remission (e.g. F32.4 - Major depressive disorder, single episode, in partial remission) were excluded.

Details on secondary analyses

Encephalopathy
Encephalopathy was defined as the presence of any of the following diagnostic codes between 4 days before and 2 weeks after the COVID-19 diagnosis:

- Other and unspecified encephalopathy (G93.4),
- Delirium (F05),
- Other mental disorders due to known physiological condition (F06),
- Personality and behavioral disorders due to known physiological condition (F07),
- Disorientation (R41.0),
- Somnolence (R40.0),
- Stupor (R40.1).

The combination of these diagnostic codes aimed to capture various clinical presentations of encephalopathy which can all represent a change from baseline cognitive status[7]. Furthermore, they account for semantic differences across disciplines. Our choice of the term 'encephalopathy' to characterise this cohort reflects both the fact that other codes are clinical manifestations of encephalopathy, and the fact that encephalopathy (G93.4) was the most prevalent code used in this cohort (present in 66.0% of patients).

Benchmarking against four additional index health events
To provide benchmarks for the incidence of neurologic and psychiatric sequelae of health events, we compared these incidences between the COVID-19 cohort and 4 other matched cohorts of patients defined by another index event (similarly to our previous study[1]):

- Skin infection: ICD-10 codes L00-L08 ('Infection of the skin and subcutaneous tissue'). This health event was selected as a common infective event that does not affect the respiratory tract.
- Urolithiasis: ICD-10 codes N20-N23. This health event was selected as a common surgical presentation.
- Fracture of a large bone: ICD-10 codes S32 ('Fracture of lumbar spine and pelvis'), S42 ('Fracture of shoulder and upper arm'), S52 ('Fracture of forearm'), S72 ('Fracture of femur'), S82 ('Fracture of lower leg including ankle'). This health event was selected as it is largely independent of prior medical conditions, and almost always leads to presentation for treatment.
- Pulmonary embolism: ICD-10 code I26. This health event was selected since, it is an acute and life-threatening event and so may have similarities to COVID-19 in terms of its psychological impact.

This analysis was achieved using the same survival analysis as in the primary analysis and by only altering the index event of the control cohort. These additional control index events were selected as they represent a broad range of common acute presentations with a range of anticipated neurologic and psychiatric sequelae.

Additional subgroup analyses
We assessed the robustness of the differences in outcomes between cohorts by repeating the analysis in 3 scenarios:

1) **Including patients who had died by the time of the analysis**: The rationale for this analysis is to assess whether differences in death rates between patients with COVID-19 and patients with influenza or other respiratory tract infections might explain the contrast between these cohorts. For instance, it might be that the more severely affected patients from one cohort (who are more likely to develop neurological or psychiatric sequelae) are more likely to survive than the more severely affected patients from another cohort. This analysis was simply achieved by removing the exclusion criterion 'Deceased on or before December 13, 2020' from the definition of cohorts.

2) **Restricting the COVID-19 diagnoses to those who had a positive RNA/Antigen test** (and using the latter as an index event): The rationale for this analysis is that a positive RNA/Antigen test better anchors the index event at the time when the virus was detected. The reason for not including it in the primary analysis is that diagnostic codes (rather than test results) are used for the definition of control cohorts and it therefore appears more valid to use a diagnostic code for the COVID-19 cohort as well. In addition, patients who had *both* a diagnostic code and a test result recorded might be a seen in HCOs which are more meticulous in their coding of diagnoses/tests and this might inflate the rate of neurological/psychiatric sequelae thus artefactually increasing the contrast between cohorts.

3) **Comparing the rates of sequelae with those observed in patients with influenza before the pandemic**: In the primary analysis, we compared the rate of psychiatric sequelae between COVID-19 and influenza within the period from January 20 to December 13, 2020. This aimed to compare health events which all occur within the same time window. However, it might be that in 2020 influenza was particularly severe (e.g. if only patients with the most severe clinical presentation sought medical attention and received the diagnosis) or particularly mild (e.g. if patients at risk of having severe influenza were benefiting from lockdown and social distancing implemented in response to the COVID-19 pandemic). As a result, the influenza cohort might not be representative of patients with influenza in a pre-pandemic context. To test whether this had an effect on the contrast between groups, in this sensitivity analysis we compared the rate of neurological/psychiatric sequelae of COVID-19 (diagnosed between January 20 and December 13, 2020) to those of influenza diagnosed between January 20 and December 13, 2019 (rather than 2020).

Details on statistical analyses

In propensity score matching, the propensity score was calculated using a logistic regression (implemented by the function LogisticRegression of the scikit-learn package in Python 3.7) including each of the covariates mentioned above. To eliminate the influence of ordering of records, the order of the records in the covariate matrix were randomised before matching.

Testing proportional hazards
The assumption that the hazards were proportional when accounting for the two phases was tested using the generalized Schoenfeld approach implemented in the cox.zph function of the survival package (version 3.2.3) in R. If the proportional hazard assumption was found to be violated, then the time-varying HR was assessed using natural cubic splines (in log-time) to the log-cumulative hazard[8]. This was achieved using the generalized survival models of the rstpm2 package (version 1.5.1) in R[9]. As recommended by Royston and Parmar[8], splines with 1, 2, and 3 degrees of freedom were estimated for both the baseline log-cumulative hazard and its cohort dependency and the number of degrees of freedom leading to the lowest Akaike Information Criterion (AIC) was selected. This was achieved on a per-comparison basis so that more complex time dependency (i.e. higher number of degrees of freedom) could be selected for a specific comparison if there was enough evidence in the data to support such complexity.

Protocol. Because of the time sensitivity of these data, a protocol was not produced prior to the analyses.

Supplementary figures

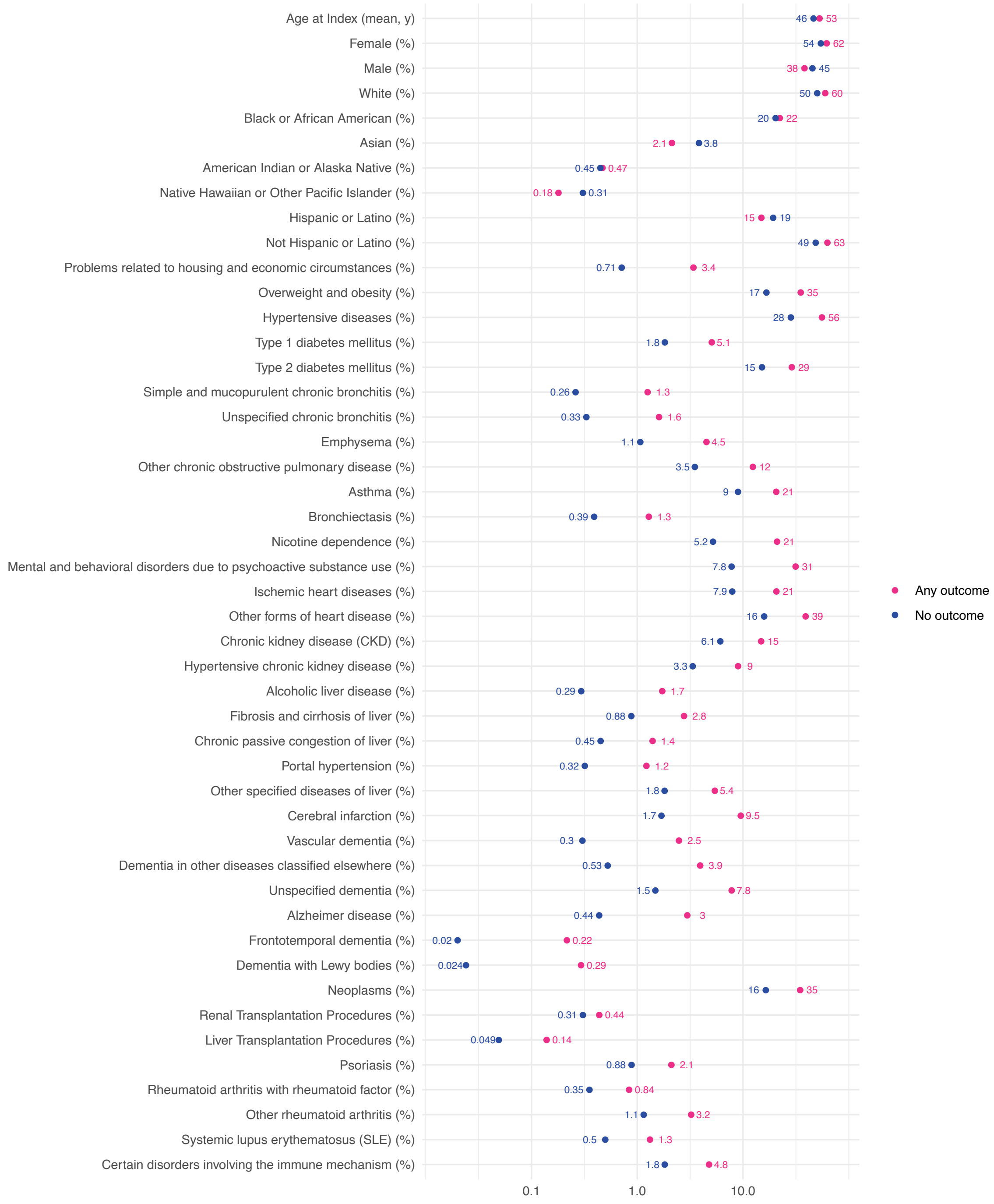

Figure S1 – Baseline characteristics of the subgroups of patients with COVID-19 who developed any vs. none of the neurological/psychiatric outcomes in the 6-months after their diagnosis. These estimates were based on patients who had their diagnosis of COVID-19 on or before August 19, 2020 thus allowing at least 6 months of follow up until February 19, 2021 when this analysis was performed.

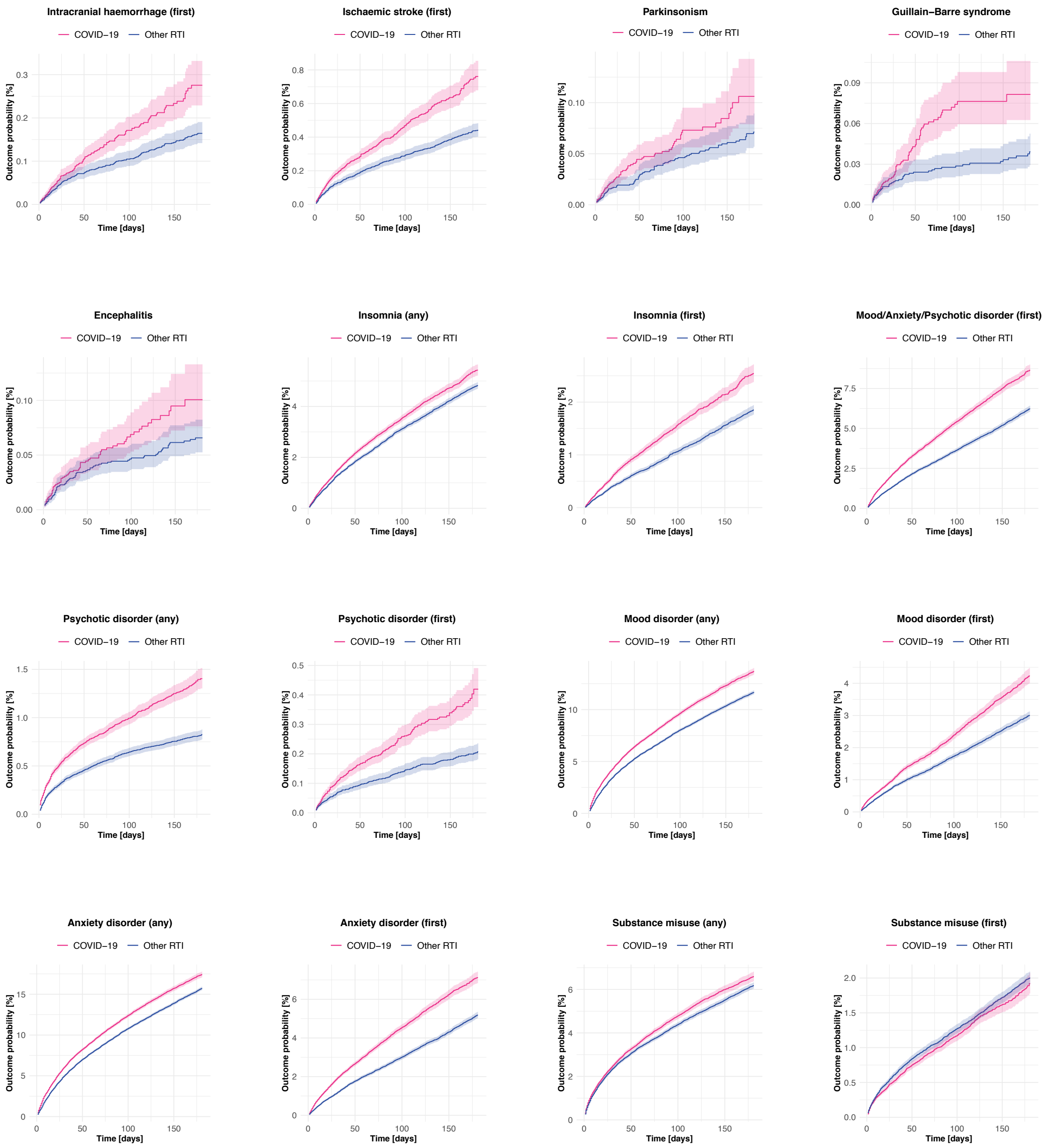

Figure S2 – Kaplan-Meier curves for the comparison between the cohort of patients with COVID-19 and a matched cohort of patients with other respiratory tract infections for the outcomes not included in Figure S1. Shaded areas represent 95% confidence intervals.

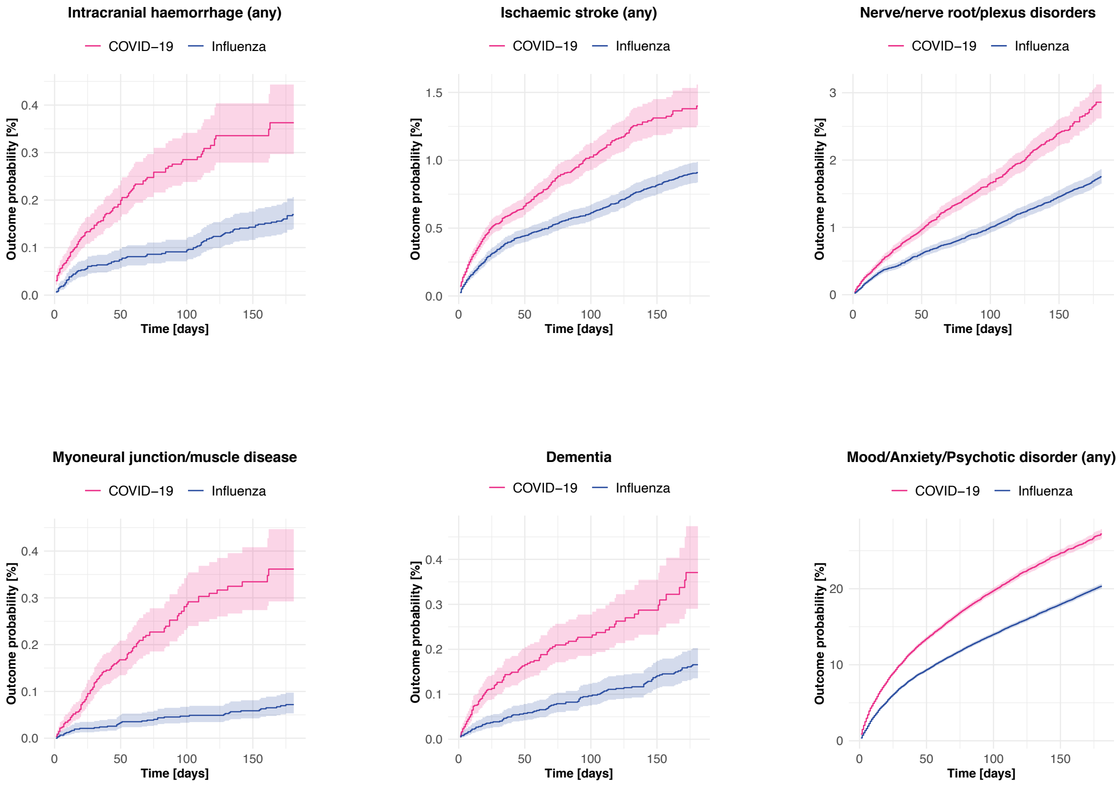

Figure S3 – Kaplan-Meier curves for the comparison between the cohort of patients with COVID-19 and a matched cohort of patients with influenza for the same outcomes as in Figure 1 of the main manuscript. Shaded areas represent 95% confidence intervals.

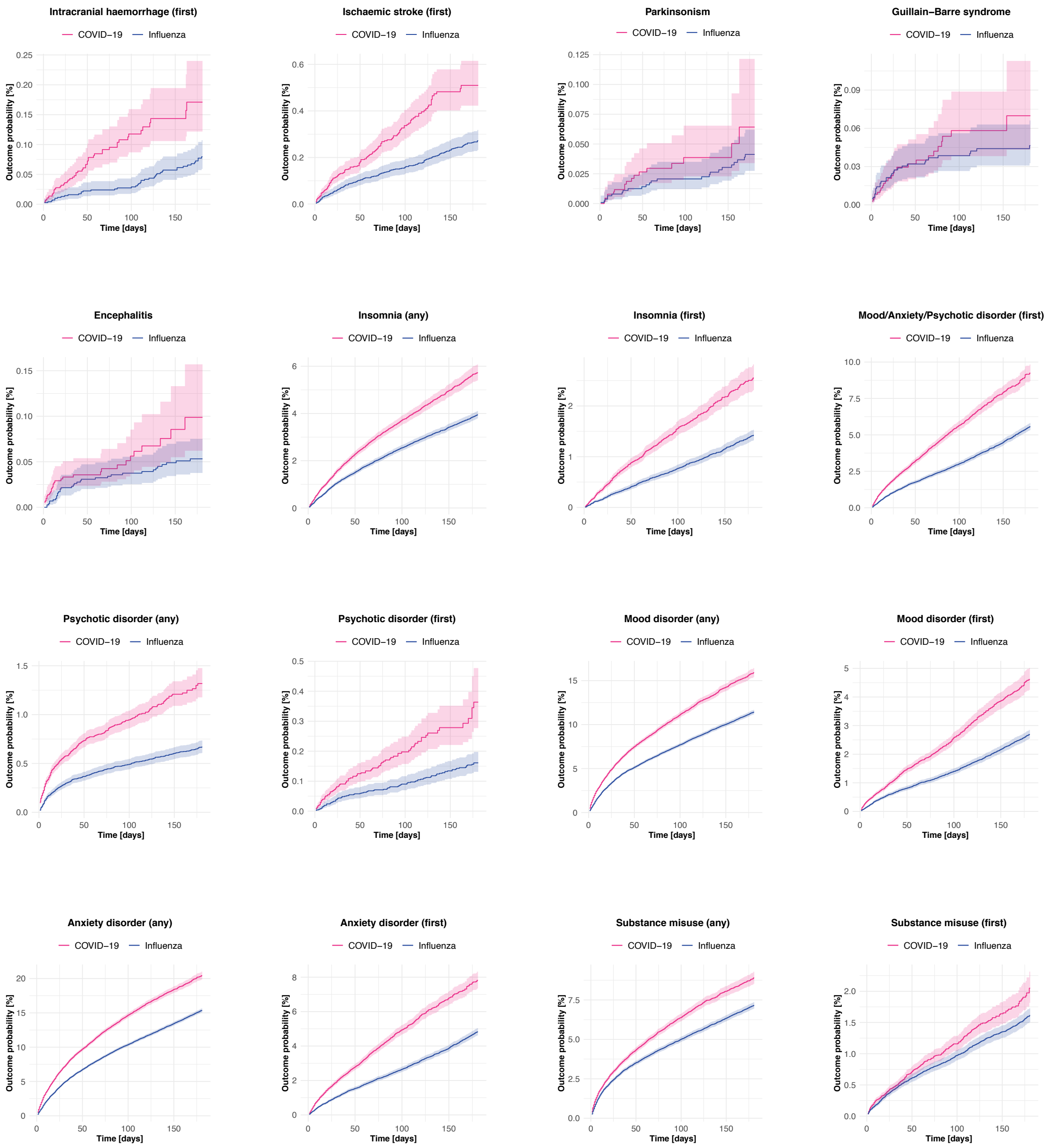

Figure S4 – Kaplan-Meier curves for the comparison between the cohort of patients with COVID-19 and a matched cohort of patients with influenza for the outcomes not included in Figure S2. Shaded areas represent 95% confidence intervals.

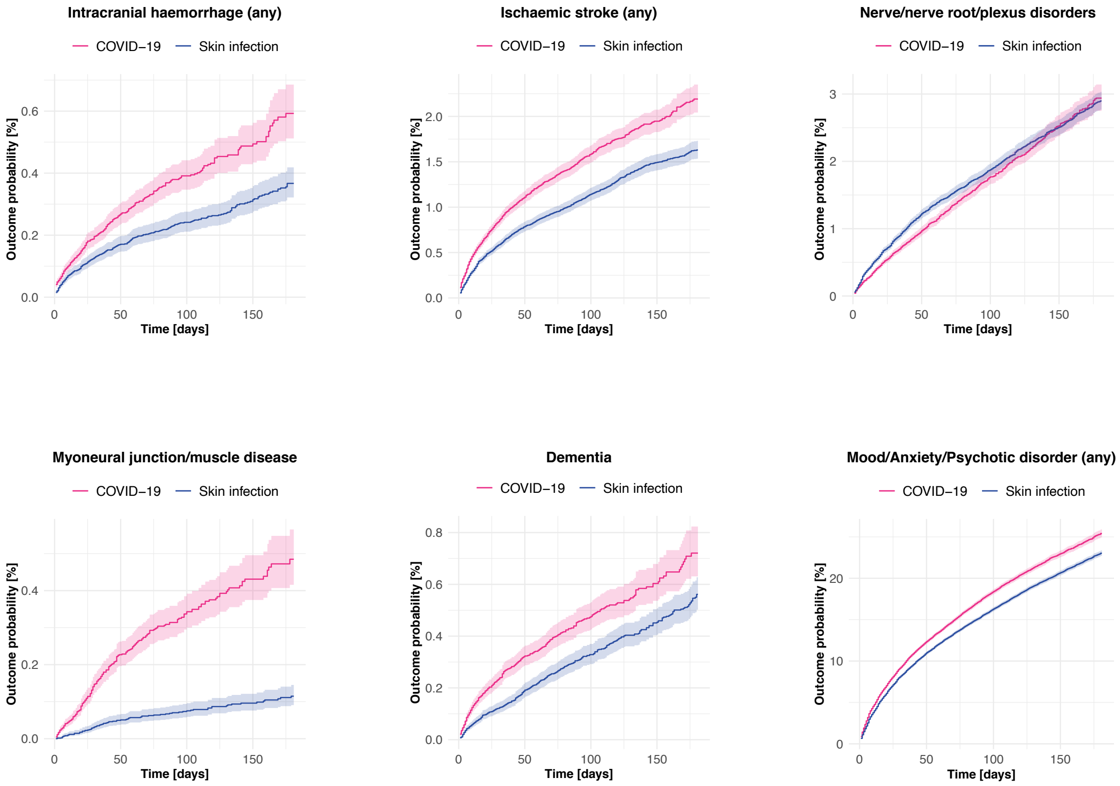

Figure S5 – Kaplan-Meier curves for the comparison between the cohort of patients with COVID-19 and a matched cohort of patients with skin infection for the same outcomes as in Figure 1 of the main manuscript. Shaded areas represent 95% confidence intervals.

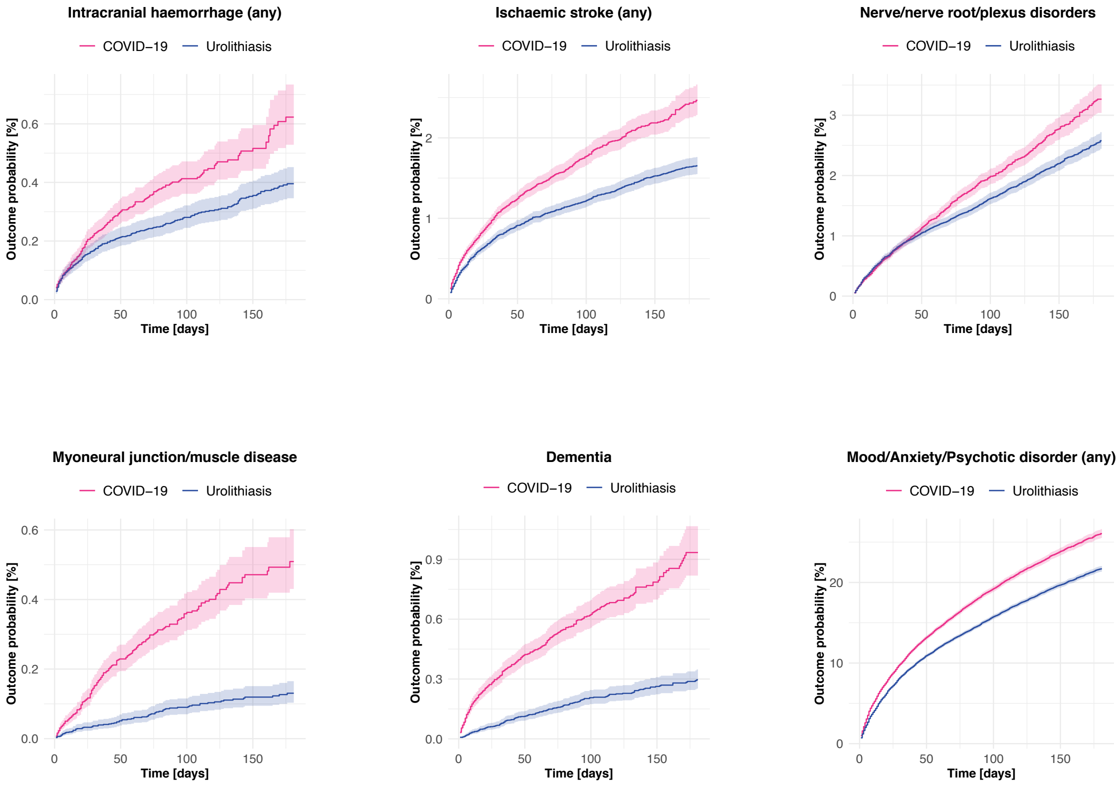

Figure S6 – Kaplan-Meier curves for the comparison between the cohort of patients with COVID-19 and a matched cohort of patients with urolithiasis for the same outcomes as in Figure 1 of the main manuscript. Shaded areas represent 95% confidence intervals.

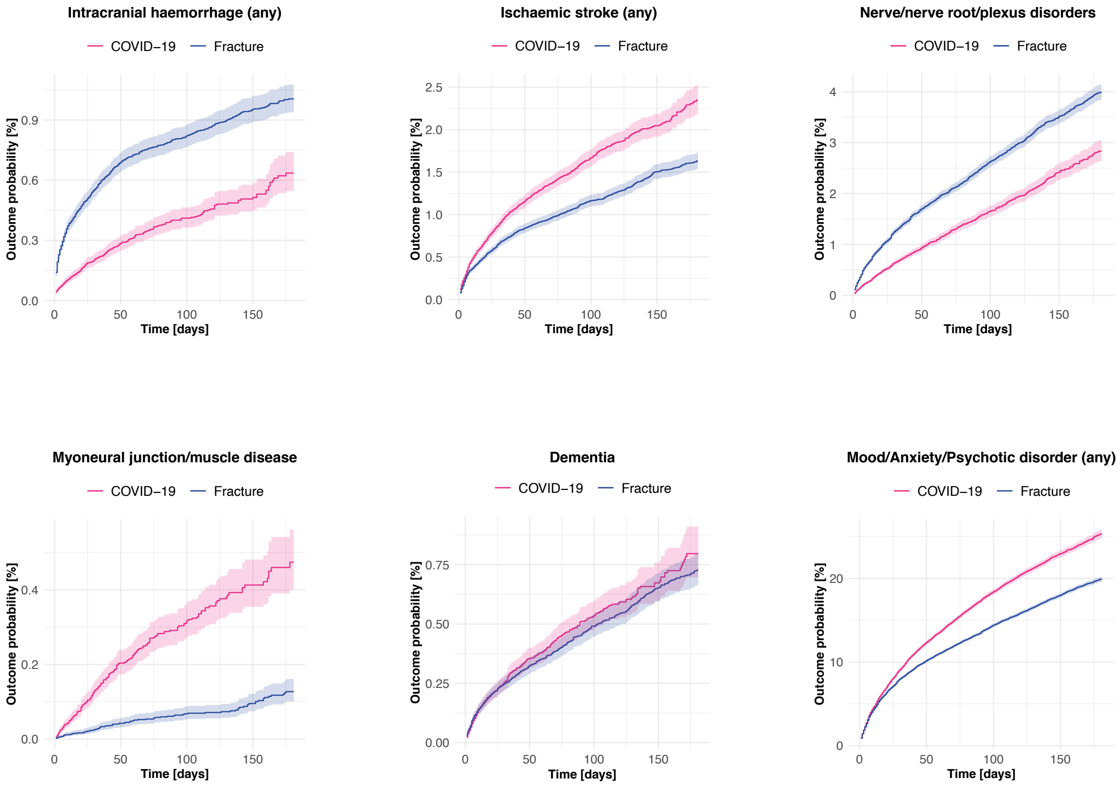

Figure S7 – Kaplan-Meier curves for the comparison between the cohort of patients with COVID-19 and a matched cohort of patients with fracture of a large bone for the same outcomes as in Figure 1 of the main manuscript. Shaded areas represent 95% confidence intervals.

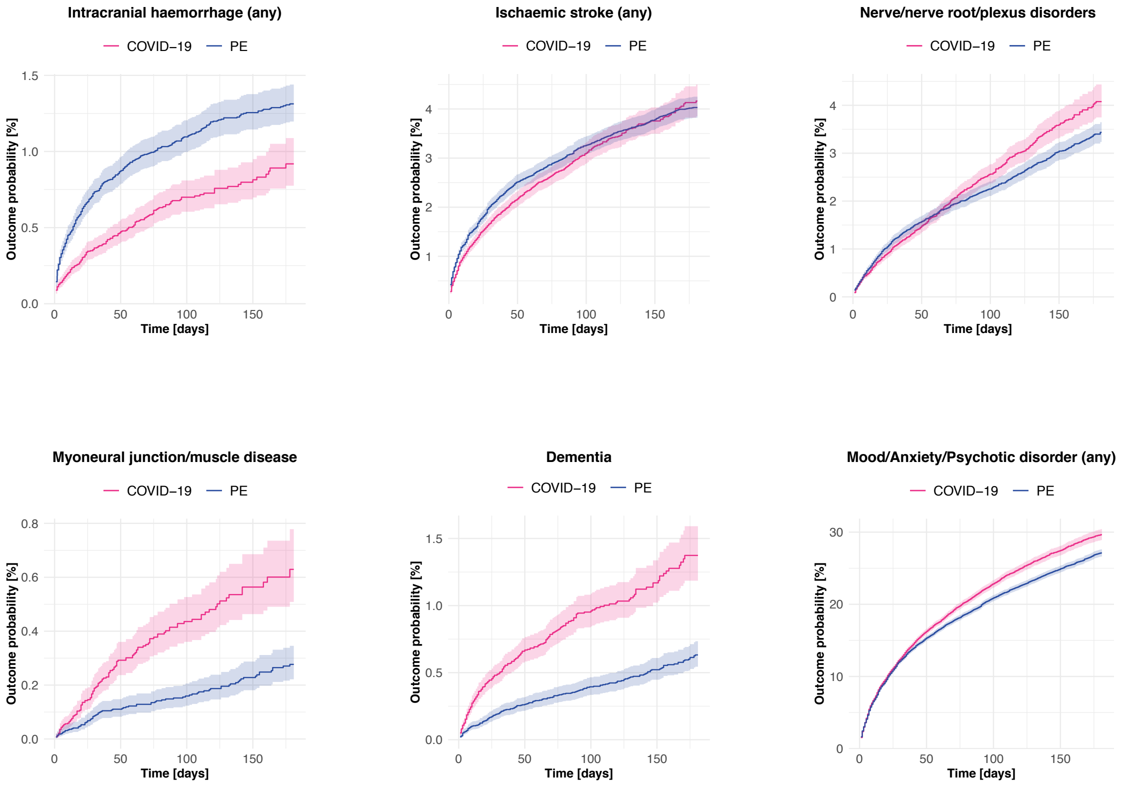

Figure S8 – Kaplan-Meier curves for the comparison between the cohort of patients with COVID-19 and a matched cohort of patients with pulmonary embolism (PE) for the same outcomes as in Figure 1 of the main manuscript. Shaded areas represent 95% confidence intervals.

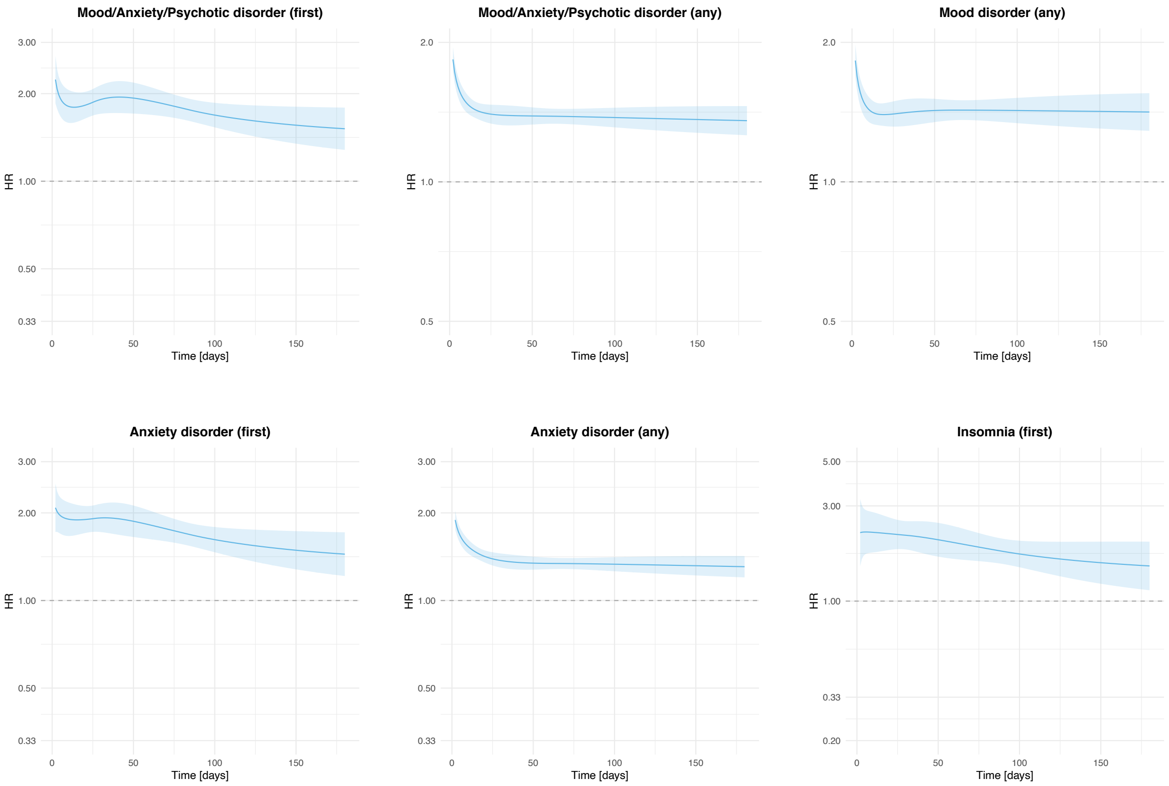

Figure S9 – Time-varying hazard ratios for the outcomes for which there was evidence of non-proportionality of hazards in the main comparison between the cohort of patients with COVID-19 and a matched cohort of patients with influenza. Shaded area represents a 95% confidence interval.

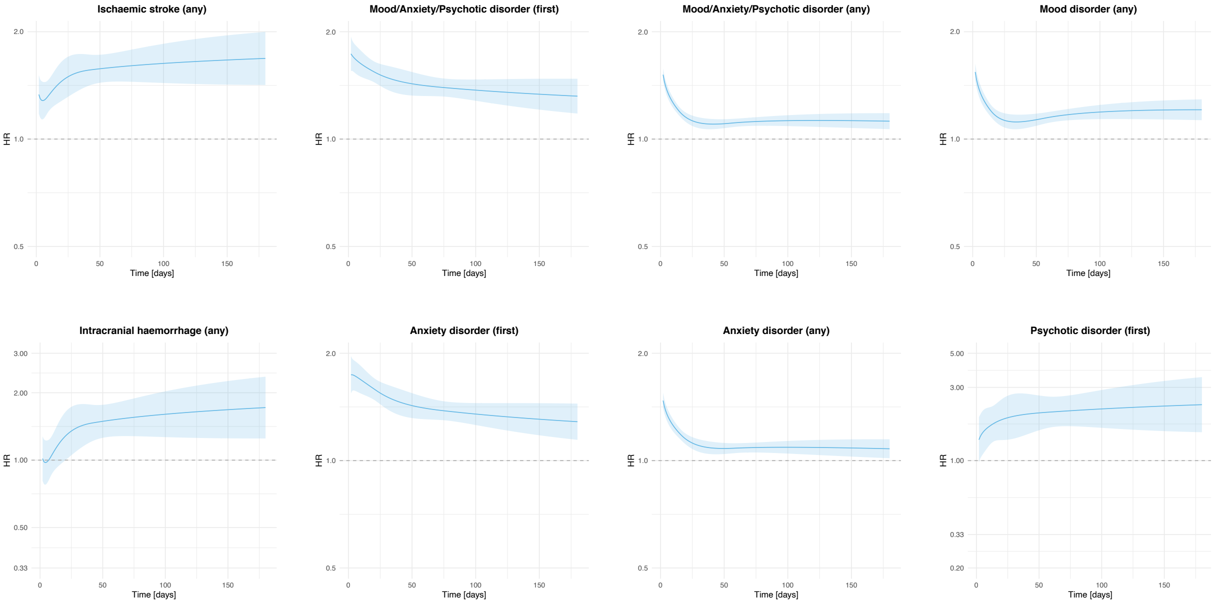

Figure S10 – Time-varying hazard ratios for the outcomes for which there was evidence of non-proportionality of hazards in the main comparison between the cohort of patients with COVID-19 and a matched cohort of patients with other respiratory tract infections. Shaded area represents a 95% confidence interval.

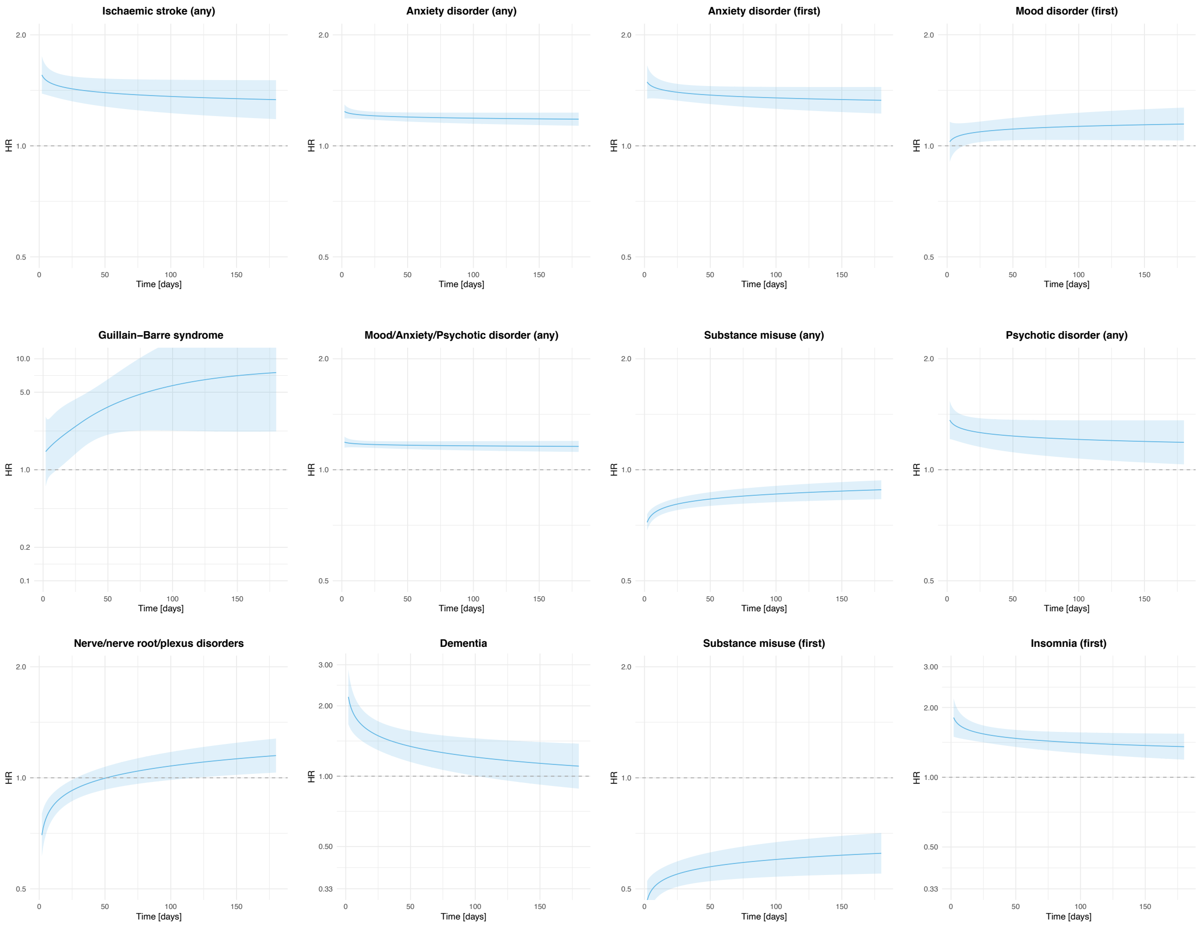

Figure S11 – Time-varying hazard ratios for the outcomes for which there was evidence of non-proportionality of hazards in the comparison between the cohort of patients with COVID-19 and a matched cohort of patients with skin infection. Shaded area represents a 95% confidence interval.

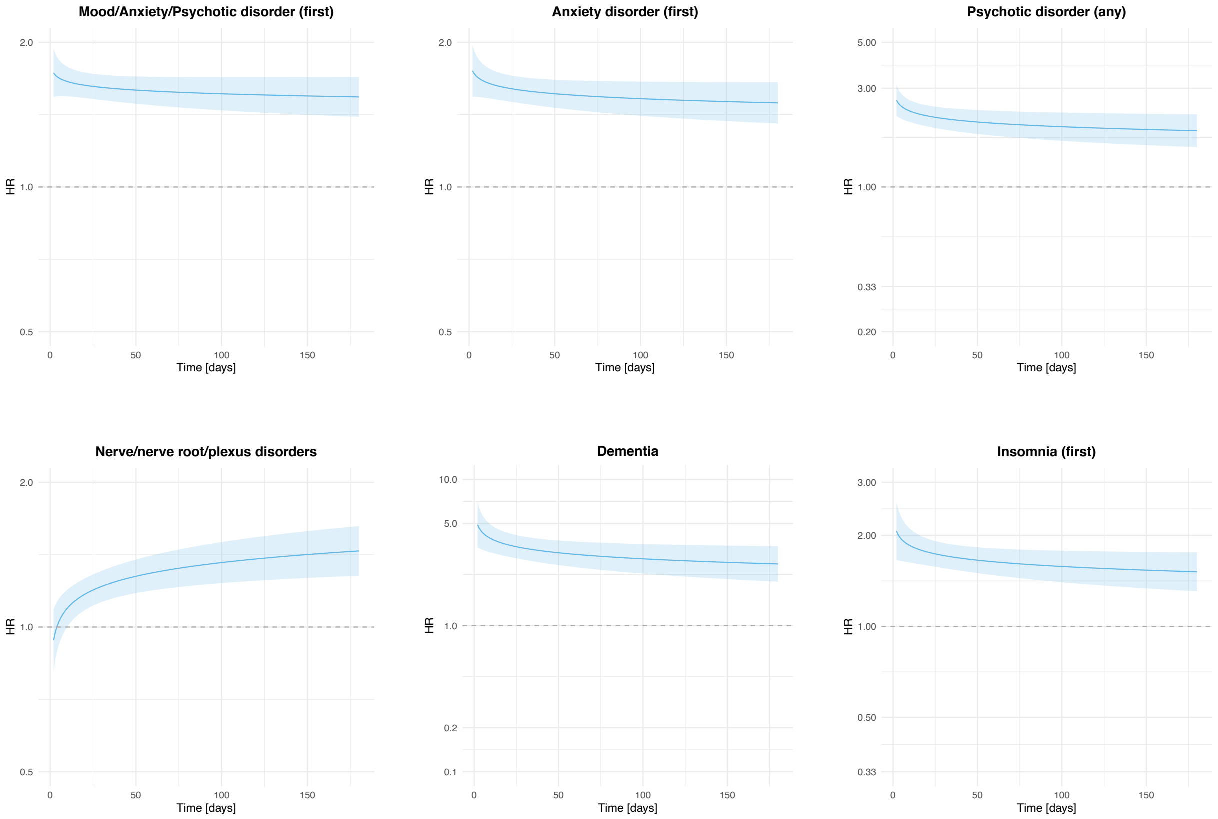

Figure S12 – Time-varying hazard ratios for the outcomes for which there was evidence of non-proportionality of hazards in the comparison between the cohort of patients with COVID-19 and a matched cohort of patients with urolithiasis. Shaded area represents a 95% confidence interval.

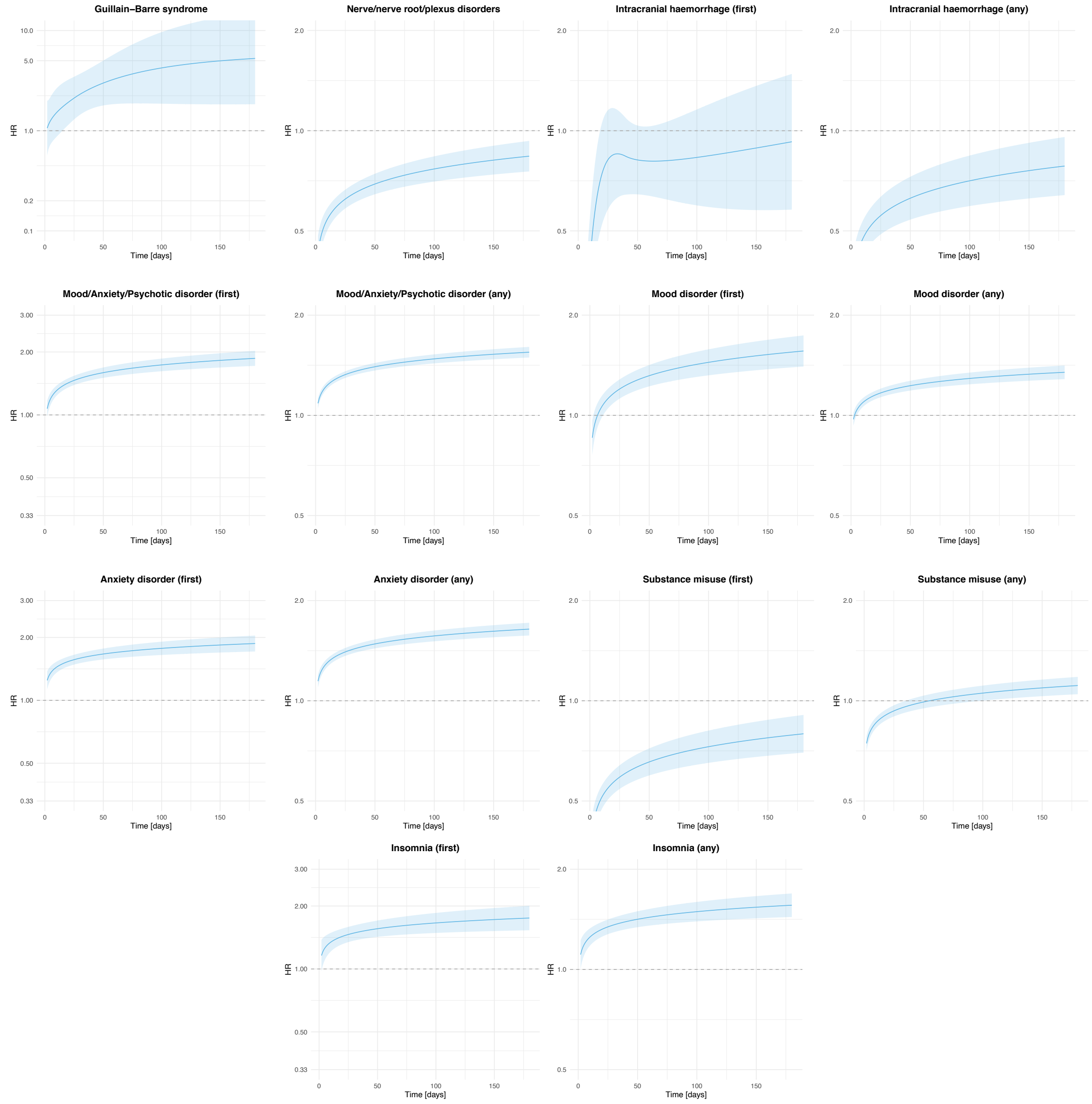

Figure S13 – Time-varying hazard ratios for the outcomes for which there was evidence of non-proportionality of hazards in the comparison between the cohort of patients with COVID-19 and a matched cohort of patients with fracture of a large bone. Shaded area represents a 95% confidence interval.

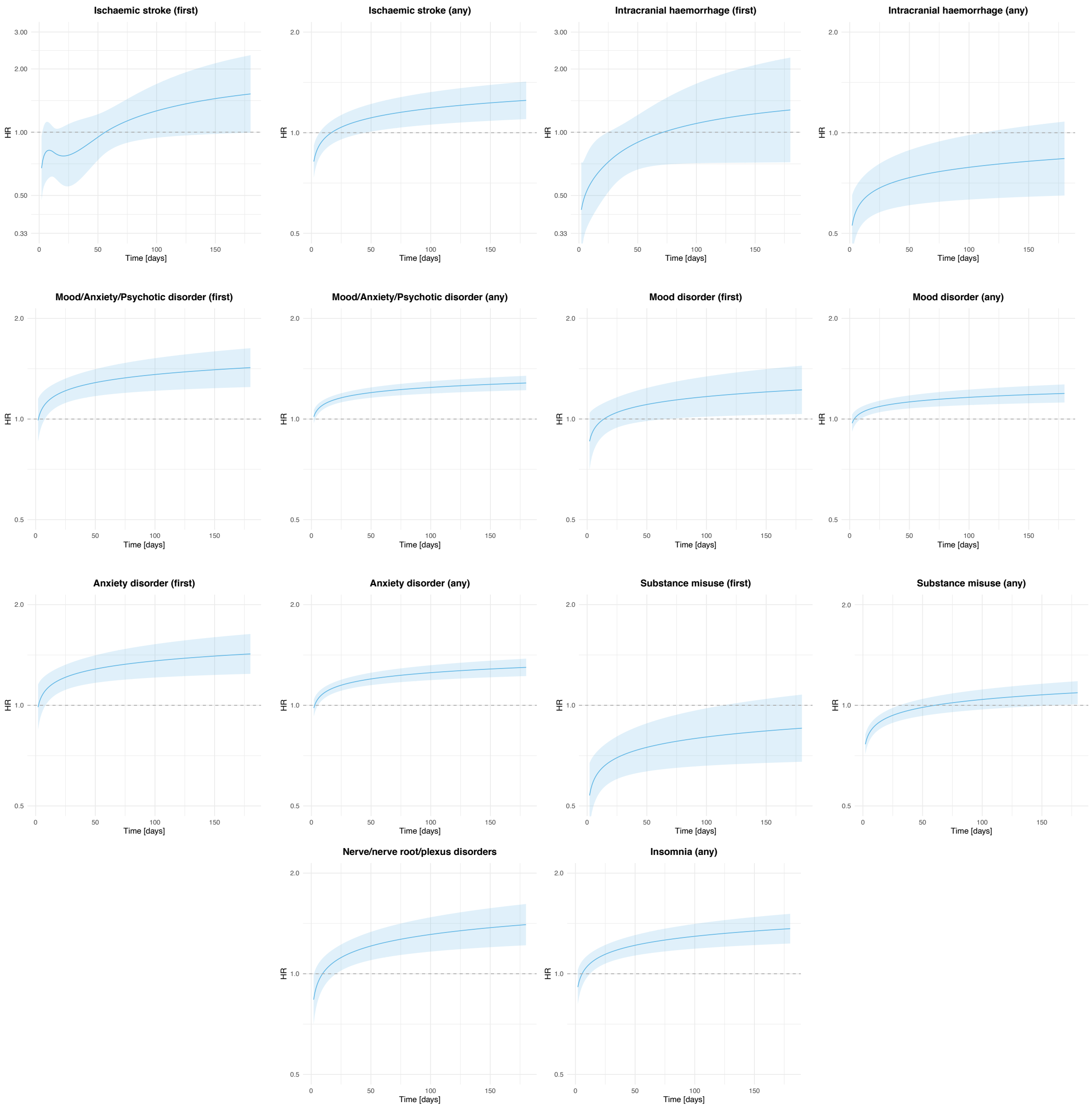

Figure S14 – Time-varying hazard ratios for the outcomes for which there was evidence of non-proportionality of hazards in the comparison between the cohort of patients with COVID-19 and a matched cohort of patients with pulmonary embolism. Shaded area represents a 95% confidence interval.

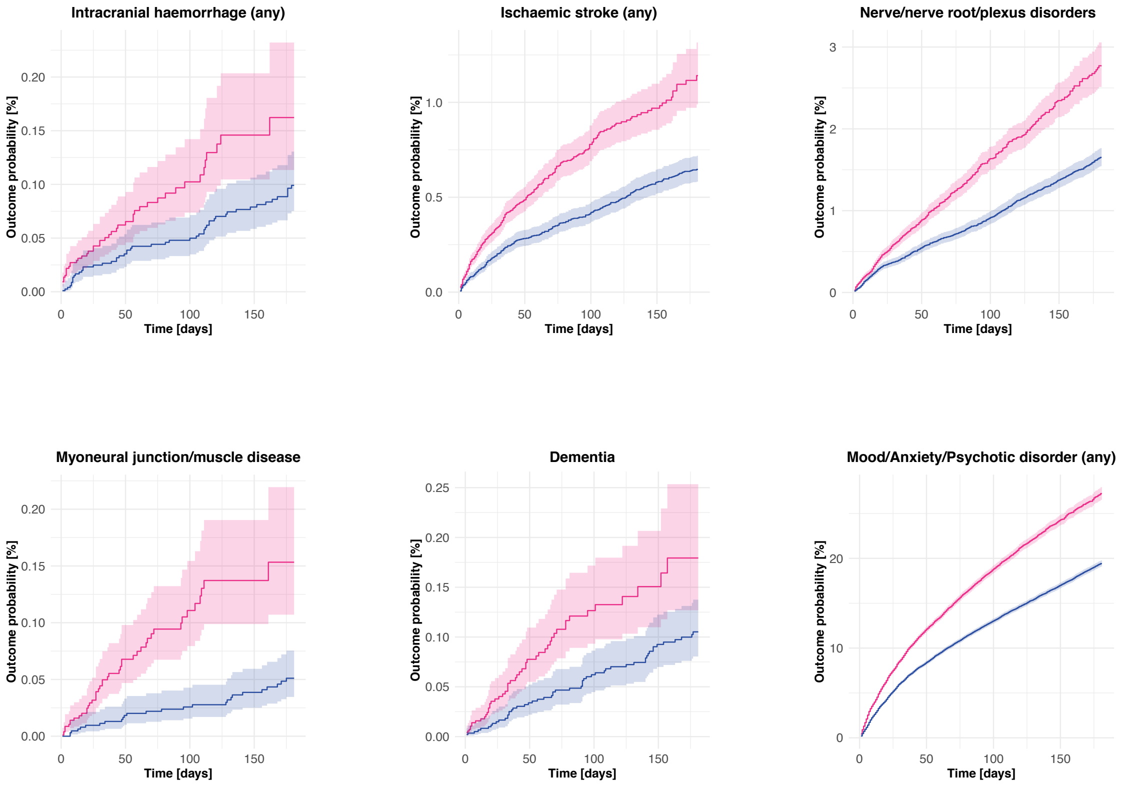

Figure S15 – Kaplan-Meier curves for the comparison between the cohort of patients with COVID-19 who did not require hospitalization (red) and a matched cohort of patients with influenza who did not require hospitalization (blue) for the same outcomes as in Figure 1 of the main manuscript. Shaded areas represent 95% confidence intervals.

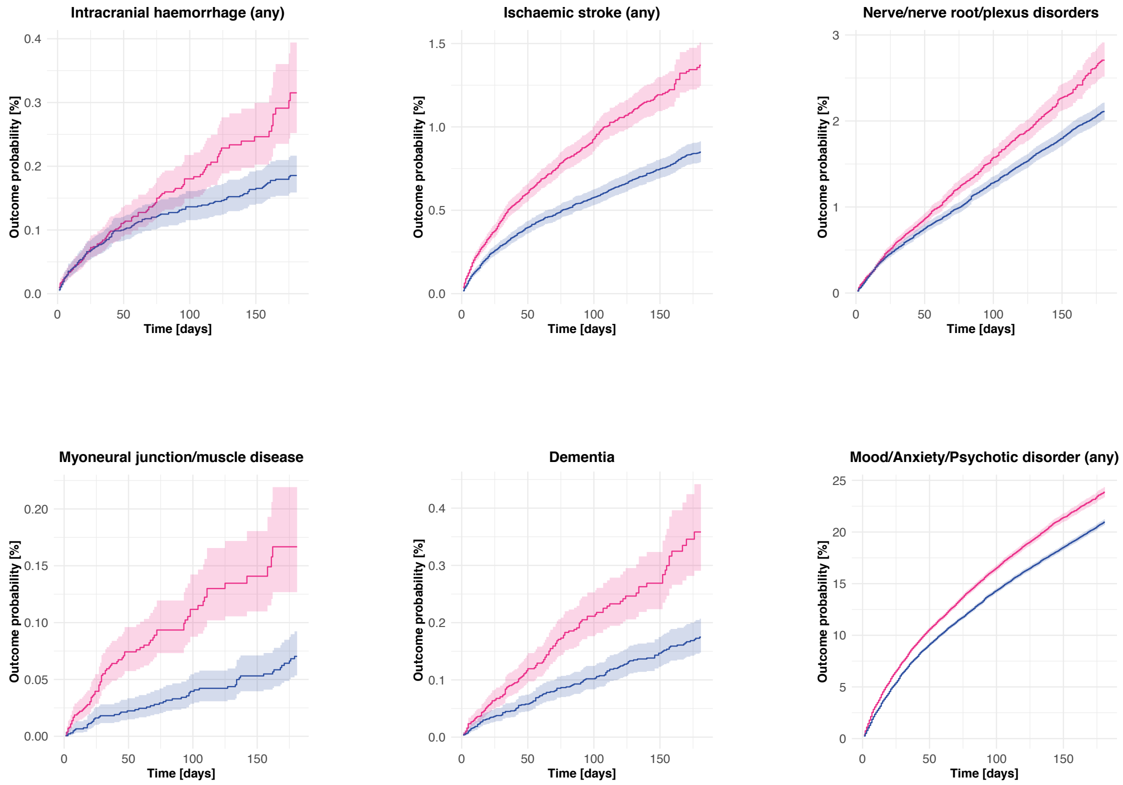

Figure S16 – Kaplan-Meier curves for the comparison between the cohort of patients with COVID-19 who did not require hospitalization (red) and a matched cohort of patients with other respiratory tract infections who did not require hospitalization (blue) for the same outcomes as in Figure 1 of the main manuscript. Shaded areas represent 95% confidence intervals.

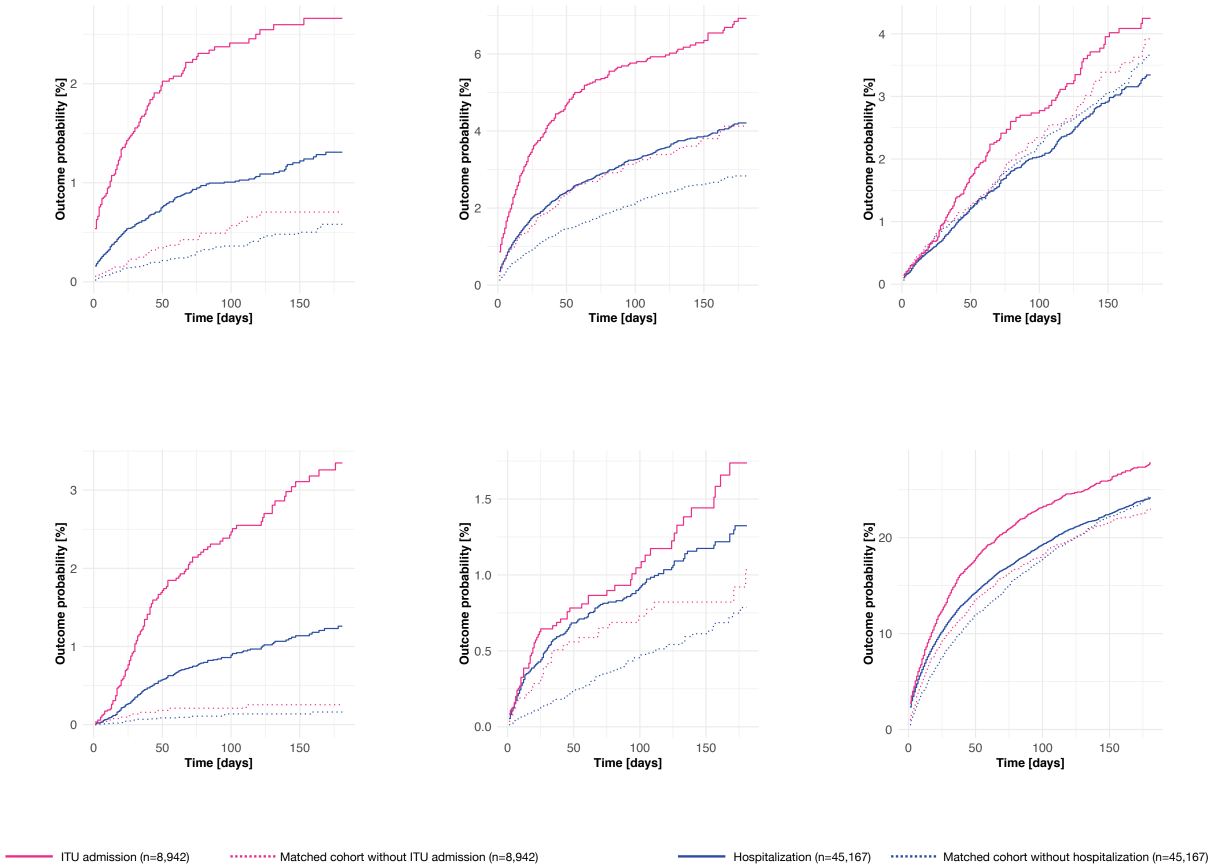

Figure S17 – Kaplan-Meier estimates for the incidence of major outcomes after COVID-19 comparing patients requiring hospitalization (solid blue line) with matched patients not requiring hospitalization (dashed blue line), and comparing those requiring an ITU admission (solid red line) with matched patients who did not require an ITU admission (dashed red line).

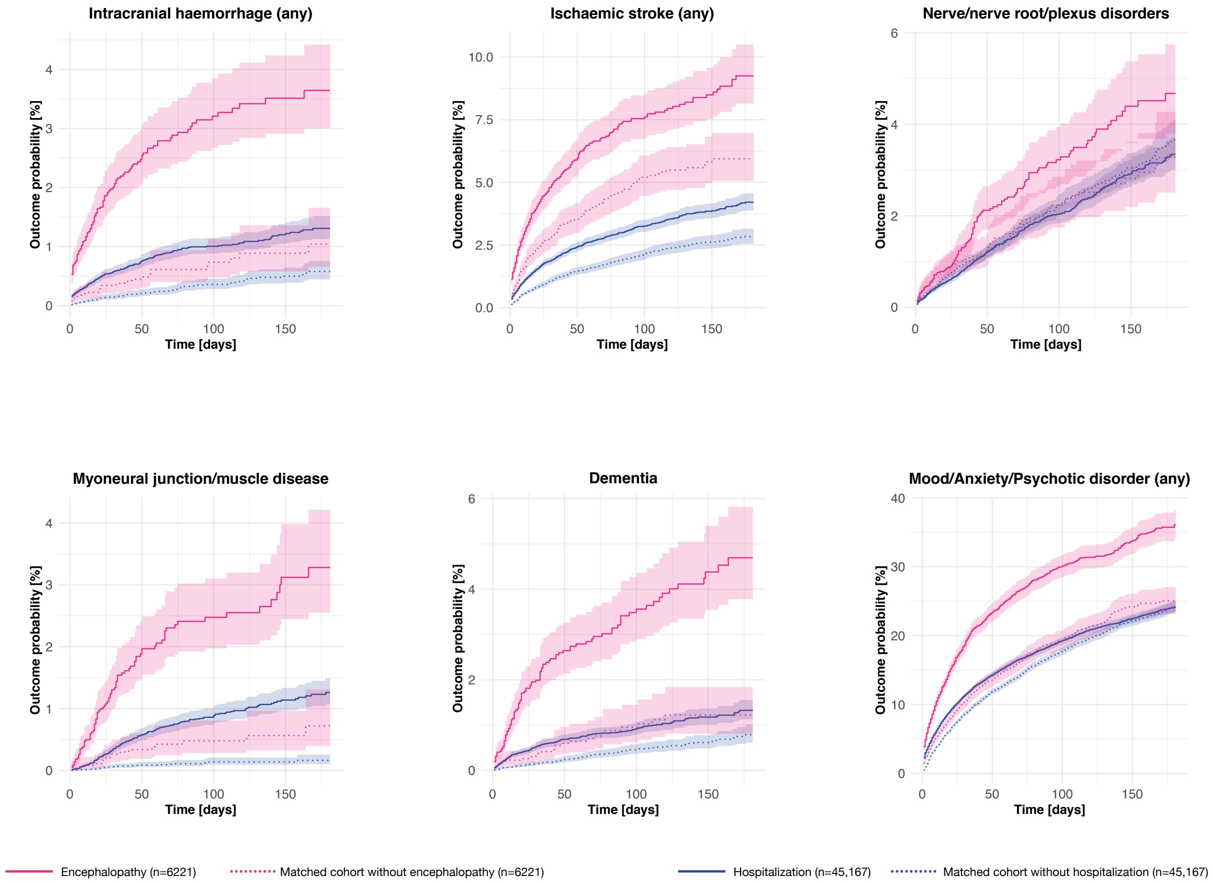

Figure S18 – Same figure as Figure 2 of the main manuscript but with 95% confidence intervals displayed

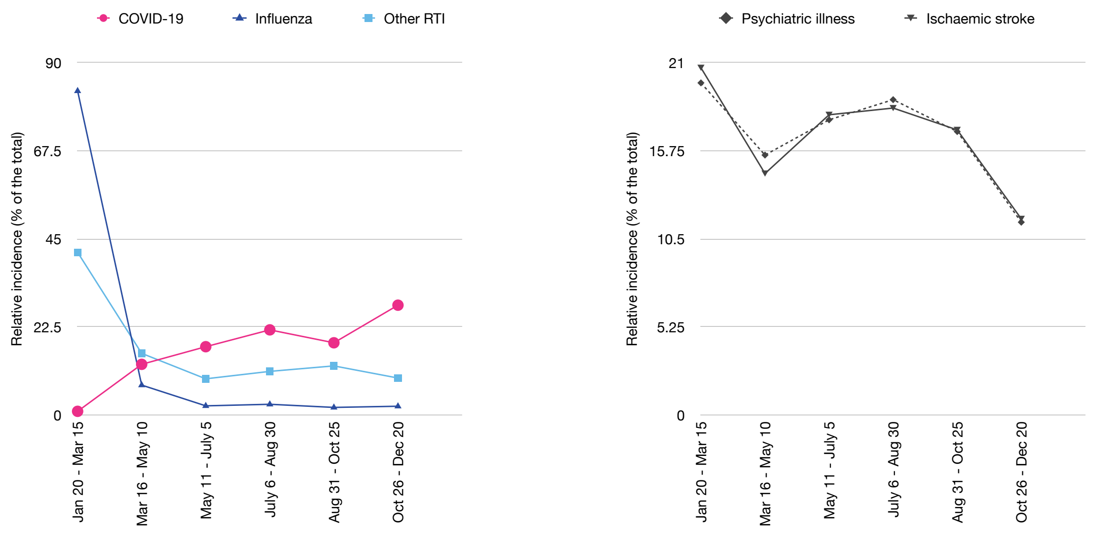

Figure S19 – 8-weekly incidence of index events (left) and two representative outcomes (right) over the study timeframe (expanded to December 20, 2020 to keep 8-weekly intervals throughout). Unlike the rates of control events, the rates of COVID-19 diagnoses mostly increased over the study timeframe. As the control events were diagnosed at a time when more outcome diagnoses were made (a well-established by-product of the pandemic), this might negatively affect the contrast between the cohorts and might imply that the reported hazard ratios are underestimated.

Supplementary tables

Table S1 – All baseline characteristics used in the propensity score matching. In addition, the incidence of neurological or psychiatric sequelae except dementia is presented. This is informative because patients who had a diagnosis of dementia (e.g. Unspecified dementia) before their diagnosis of COVID-19 and then go on to have another dementia diagnosis (e.g. Vascular dementia) would count towards the incidence of any diagnosis (alongside those for whom this would be their first diagnosis of dementia). The incidence of 'any first' diagnosis (presented in Table 1 of the main manuscript) is not affected by this limitation since it excludes all previous diagnoses of dementia.

	All patients	Non-hospitalized patients	Hospitalized patients	Patients with ITU admission	Patients with encephalopathy
**Cohort size, n (%)**	236379	190077	46302	8945	6229
**DEMOGRAPHICS**					
**Age**, mean (SD), y	46 (19.7)	43.3 (19.0)	57 (18.7)	59.1 (17.3)	66.7 (17.0)
**Sex, n (%)**					
Female	131460 (55.6)	107730 (56.7)	23730 (51.2)	3743 (41.8)	2909 (46.7)
Male	104015 (44.0)	81512 (42.9)	22503 (48.6)	5196 (58.1)	3307 (53.1)
Other	904 (0.4)	835 (0.4)	69 (0.1)	10 (0.1)	13 (0.2)
**Race, n (%)**					
White	135143 (57.2)	109635 (57.7)	25508 (55.1)	4918 (55.0)	3331 (53.5)
Black or African American	44459 (18.8)	33868 (17.8)	10591 (22.9)	2184 (24.4)	1552 (24.9)
Asian	6979 (3.0)	5432 (2.9)	1547 (3.3)	320 (3.6)	243 (3.9)
American Indian or Alaska Native	962 (0.4)	716 (0.4)	246 (0.5)	42 (0.5)	21 (0.3)
Native Hawaiian or Other Pacific Islander	751 (0.3)	585 (0.3)	166 (0.4)	24 (0.3)	11 (0.2)
Unknown	48085 (20.3)	39841 (21.0)	8244 (17.8)	1457 (16.3)	1071 (17.2)
**Ethnicity, n (%)**					
Hispanic or Latino	37772 (16.0)	29155 (15.3)	8617 (18.6)	2248 (25.1)	895 (14.4)
Not Hispanic of Latino	134075 (56.7)	106844 (56.2)	27231 (58.8)	5041 (56.4)	3873 (62.2)
Unknown	64532 (27.3)	54078 (28.5)	10454 (22.6)	1656 (18.5)	1461 (23.5)
**Problems related to housing and economic circumstances, n (%)**	2452 (1.04)	1400 (0.7)	1052 (2.27)	236 (2.6)	216 (3.5)
**COMORBIDITIES, n (%)**					
**Overweight and obesity**	42871 (18.1)	30198 (15.9)	12673 (27.4)	3062 (34.2)	1838 (29.5)
**Hypertensive disease**	71014 (30.0)	47516 (25.0)	23498 (50.7)	5569 (62.3)	4591 (73.7)
**Diabetes mellitus**					
Type 1 diabetes mellitus	5013 (2.1)	3028 (1.6)	1985 (4.3)	621 (6.9)	424 (6.8)
Type 2 diabetes mellitus	36696 (15.5)	22518 (11.8)	14178 (30.6)	3787 (42.3)	2890 (46.4)
**Chronic lower respiratory diseases**					
Bronchitis; not specified as acute or chronic	11033 (4.7)	8795 (4.6)	2238 (4.8)	511 (5.7)	362 (5.8)

Simple and mucopurulent chronic bronchitis	988 (0.4)	686 (0.4)	302 (0.7)	85 (0.9)	69 (1.1)
Unspecified chronic bronchitis	1190 (0.5)	734 (0.4)	456 (1.0)	117 (1.3)	81 (1.3)
Emphysema	3454 (1.5)	1947 (1.0)	1507 (3.3)	425 (4.8)	314 (5.0)
Other chronic obstructive pulmonary disease	10564 (4.5)	5714 (3.0)	4850 (10.5)	1254 (14.0)	1030 (16.5)
Asthma	25104 (10.6)	19834 (10.4)	5270 (11.4)	1132 (12.7)	755 (12.1)
Bronchiectasis	1183 (0.5)	719 (0.4)	464 (1.0)	128 (1.4)	89 (1.4)
**Nicotine dependence**	17105 (7.2)	12639 (6.6)	4466 (9.6)	1042 (11.6)	803 (12.9)
**Substance misuse**	24870 (10.5)	18173 (9.6)	6697 (14.5)	1620 (18.1)	1316 (21.1)
**Heart disease**					
Ischemic heart diseases	21082 (8.9)	11815 (6.2)	9267 (20.0)	2460 (27.5)	2200 (35.3)
Other forms of heart disease	42431 (18.0)	26066 (13.7)	16365 (35.3)	4678 (52.3)	3694 (59.3)
**Chronic kidney diseases**					
Chronic kidney disease (CKD)	15908 (6.7)	8345 (4.4)	7563 (16.3)	1941 (21.7)	1892 (30.4)
Hypertensive chronic kidney disease	8943 (3.8)	4460 (2.3)	4483 (9.7)	1168 (13.1)	1247 (20.0)
**Chronic liver disease**					
Alcoholic liver disease	1091 (0.5)	559 (0.3)	532 (1.1)	140 (1.6)	134 (2.2)
Hepatic failure; not elsewhere classified	1409 (0.6)	621 (0.3)	788 (1.7)	316 (3.5)	285 (4.6)
Chronic hepatitis; not elsewhere classified	309 (0.1)	200 (0.1)	109 (0.2)	34 (0.4)	29 (0.5)
Fibrosis and cirrhosis of liver	2532 (1.1)	1399 (0.7)	1133 (2.4)	299 (3.3)	248 (4.0)
Fatty (change of) liver; not elsewhere classified	8190 (3.5)	5882 (3.1)	2308 (5.0)	550 (6.1)	353 (5.7)
Chronic passive congestion of liver	1330 (0.6)	930 (0.5)	400 (0.9)	114 (1.3)	85 (1.4)
Portal hypertension	1046 (0.4)	540 (0.3)	506 (1.1)	129 (1.4)	120 (1.9)
Other specified diseases of liver	5369 (2.3)	3813 (2.0)	1556 (3.4)	400 (4.5)	293 (4.7)
**Cerebral infarction**	5858 (2.5)	3178 (1.7)	2680 (5.8)	776 (8.7)	925 (14.8)
**Dementia**					
Vascular dementia	1292 (0.5)	648 (0.3)	644 (1.4)	146 (1.6)	332 (5.3)
Dementia in other diseases classified elsewhere	2113 (0.9)	1109 (0.6)	1004 (2.2)	193 (2.2)	551 (8.8)
Unspecified dementia	4625 (2.0)	2144 (1.1)	2481 (5.4)	546 (6.1)	1202 (19.3)
Alzheimer disease	1652 (0.7)	884 (0.5)	768 (1.7)	127 (1.4)	371 (6.0)
Frontotemporal dementia	102 (0.04)	57 (0.03)	45 (0.1)	10 (0.1)	23 (0.4)
Dementia with Lewy bodies	139 (0.06)	75 (0.04)	64 (0.1)	10 (0.1)	45 (0.7)
**Neoplasms**					
Neoplasms (any)	45255 (19.1)	34362 (18.1)	10893 (23.5)	2339 (26.1)	1793 (28.8)
Malignant neoplasms of lymphoid; hematopoietic and related tissue	2655 (1.12)	1598 (0.8)	1057 (2.3)	253 (2.8)	162 (2.6)

**Organ transplant**					
Renal Transplantation Procedures	741 (0.3)	351 (0.2)	390 (0.8)	103 (1.2)	46 (0.7)
Liver Transplantation Procedures	145 (0.1)	73 (0.04)	72 (0.2)	18 (0.2)	11 (0.2)
**Psoriasis**	2528 (1.1)	1968 (1.0)	560 (1.2)	136 (1.5)	83 (1.3)
**Rheumatoid arthritis**					
Rheumatoid arthritis with rheumatoid factor	972 (0.4)	732 (0.4)	240 (0.5)	64 (0.7)	40 (0.6)
Other rheumatoid arthritis	3328 (1.4)	2299 (1.2)	1029 (2.2)	227 (2.5)	175 (2.8)
**Systemic lupus erythematosus (SLE)**	1417 (0.6)	1046 (0.6)	371 (0.8)	78 (0.9)	51 (0.8)
**Disorders involving the immune mechanism**	5215 (2.2)	3437 (1.8)	1778 (3.8)	385 (4.3)	269 (4.3)
**OUTCOMES**					
**Any**	33.62 (33.17-34.07)	31.74 (31.22-32.27)	38.73 (37.87-39.60)	46.42 (44.78-48.09)	62.34 (60.14-64.55)
**Any (excluding dementia)**	32.59 (32.16-33.03)	31.18 (30.67-31.70)	36.32 (35.50-37.15)	44.26 (42.62-45.93)	55.41 (53.28-57.57)

Table S2 – Incidence of neurological and psychiatric outcomes within 6 months after a diagnosis of COVID-19 separately estimated by gender, race and age group

	**Female**	**Male**	**Asian**	**Black**	**White**	**Over 65**	**Under 65**
	% (95% CI)	% (95% CI)	% (95% CI)	% (95% CI)	% (95% CI)	% (95% CI)	% (95% CI)
Intracranial haemorrhage (any)	0.40 (0.33-0.49)	0.81 (0.68-0.97)	1.09 (0.58-2.04)	0.55 (0.43-0.70)	0.56 (0.47-0.68)	1.21 (1.02-1.44)	0.35 (0.30-0.42)
Intracranial haemorrhage (first)	0.18 (0.13-0.25)	0.42 (0.33-0.54)	0.62 (0.23-1.67)	0.24 (0.16-0.35)	0.28 (0.21-0.37)	0.62 (0.48-0.81)	0.16 (0.12-0.21)
Ischaemic stroke (any)	1.73 (1.58-1.90)	2.51 (2.29-2.75)	2.01 (1.35-2.99)	2.70 (2.42-3.00)	1.82 (1.66-2.00)	4.75 (4.38-5.14)	1.15 (1.03-1.27)
Ischaemic stroke (first)	0.64 (0.53-0.76)	0.90 (0.76-1.06)	1.11 (0.62-1.97)	0.96 (0.78-1.18)	0.59 (0.49-0.71)	1.65 (1.40-1.94)	0.45 (0.38-0.54)
Parkinsonism	0.045 (0.029-0.069)	0.19 (0.13-0.28)	0.09 (0.027-0.30)	0.078 (0.033-0.18)	0.11 (0.077-0.16)	0.35 (0.25-0.48)	0.022 (0.011-0.046)
Guillain-Barre syndrome	0.073 (0.049-0.11)	0.11 (0.074-0.15)	0.15 (0.041-0.55)	0.033 (0.016-0.07)	0.10 (0.074-0.15)	0.13 (0.078-0.21)	0.07 (0.051-0.095)
Nerve/nerve root/plexus disorders	3.15 (2.92-3.39)	2.46 (2.21-2.73)	2.29 (1.60-3.28)	2.99 (2.66-3.35)	2.92 (2.69-3.17)	3.35 (3.00-3.73)	2.65 (2.46-2.85)
Myoneural junction/muscle disease	0.33 (0.27-0.41)	0.63 (0.52-0.77)	0.38 (0.20-0.74)	0.40 (0.29-0.54)	0.42 (0.34-0.51)	0.61 (0.49-0.76)	0.39 (0.33-0.48)
Encephalitis	0.087 (0.058-0.13)	0.13 (0.085-0.19)	0.28 (0.099-0.77)	0.13 (0.073-0.24)	0.094 (0.066-0.13)	0.15 (0.095-0.24)	0.093 (0.064-0.13)
Dementia	0.69 (0.59-0.82)	0.65 (0.54-0.78)	0.62 (0.32-1.19)	0.67 (0.53-0.85)	0.75 (0.63-0.88)	2.66 (2.35-3.02)	0.10 (0.069-0.14)
Mood/Anxiety/Psychotic disorder (any)	28.00 (27.43-28.57)	18.38 (17.78-18.99)	15.71 (13.81-17.85)	21.05 (20.29-21.84)	27.47 (26.87-28.09)	23.42 (22.65-24.22)	23.85 (23.36-24.35)
Mood/Anxiety/Psychotic disorder (first)	10.08 (9.55-10.62)	6.91 (6.44-7.42)	7.31 (5.74-9.29)	7.95 (7.30-8.65)	9.43 (8.89-10.01)	7.68 (7.07-8.34)	8.75 (8.33-9.20)
Mood disorder (any)	16.20 (15.74-16.67)	9.92 (9.46-10.40)	8.45 (6.92-10.31)	11.66 (11.07-12.29)	15.86 (15.37-16.36)	14.52 (13.89-15.18)	13.08 (12.70-13.48)
Mood disorder (first)	5.07 (4.72-5.43)	3.26 (2.93-3.64)	3.38 (2.23-5.10)	3.71 (3.27-4.20)	4.75 (4.40-5.14)	4.07 (3.63-4.57)	4.24 (3.96-4.54)
Anxiety disorder (any)	20.94 (20.44-21.46)	12.50 (11.99-13.03)	10.87 (9.25-12.75)	14.90 (14.24-15.60)	20.08 (19.54-20.62)	14.21 (13.58-14.87)	18.26 (17.82-18.70)
Anxiety disorder (first)	8.52 (8.08-8.99)	5.33 (4.94-5.74)	6.30 (4.82-8.21)	6.59 (6.05-7.18)	7.66 (7.21-8.15)	5.48 (4.99-6.02)	7.50 (7.14-7.88)
Psychotic disorder (any)	1.22 (1.10-1.37)	1.81 (1.62-2.03)	1.05 (0.63-1.76)	1.89 (1.67-2.15)	1.42 (1.27-1.60)	2.27 (2.01-2.58)	1.16 (1.05-1.28)
Psychotic disorder (first)	0.38 (0.30-0.48)	0.52 (0.41-0.65)	0.30 (0.13-0.67)	0.52 (0.40-0.69)	0.45 (0.36-0.57)	0.82 (0.65-1.03)	0.30 (0.24-0.38)
Substance misuse (any)	5.45 (5.18-5.74)	8.48 (8.07-8.91)	1.86 (1.36-2.54)	8.29 (7.79-8.82)	6.68 (6.37-7.00)	5.32 (4.91-5.77)	6.88 (6.61-7.16)
Substance misuse (first)	1.42 (1.26-1.61)	2.59 (2.32-2.89)	0.65 (0.37-1.12)	2.22 (1.89-2.60)	1.84 (1.65-2.04)	1.46 (1.22-1.73)	1.95 (1.78-2.14)
Insomnia (any)	5.80 (5.50-6.12)	5.07 (4.73-5.44)	4.51 (3.31-6.12)	5.03 (4.62-5.47)	6.03 (5.71-6.37)	6.87 (6.40-7.38)	4.95 (4.69-5.21)
Insomnia (first)	2.73 (2.49-2.99)	2.28 (2.05-2.53)	2.67 (1.68-4.25)	2.46 (2.16-2.81)	2.60 (2.37-2.86)	2.87 (2.53-3.26)	2.39 (2.20-2.60)

Table S3 – Characteristics of the COVID-19 and influenza cohorts after propensity-score matching. SMD=Standardized Mean Difference.

	COVID-19	Influenza	SMD
Number	105579	105579	-
DEMOGRAPHICS			
Age; mean (SD); y	39.7 (18.4)	38.6 (19.7)	0.06
Sex; n (%)			
Female	61831 (58.6)	60828 (57.6)	0.02
Male	43709 (41.4)	44707 (42.3)	0.02
Other	39 (0.04)	44 (0.04)	0.002
Race; n (%)			
White	69730 (66.0)	69294 (65.6)	0.009
Black or African American	19175 (18.2)	18466 (17.5)	0.02
Asian	3724 (3.5)	3488 (3.3)	0.01
American Indian or Alaska Native	442 (0.4)	431 (0.4)	0.002
Native Hawaiian or Other Pacific Islander	214 (0.2)	219 (0.2)	0.001
Unknown	12294 (11.6)	13681 (13.0)	0.04
Ethnicity; n (%)			
Hispanic or Latino	8841 (8.4)	8932 (8.5)	0.003
Not Hispanic of Latino	72389 (68.6)	71409 (67.6)	0.02
Unknown	24349 (23.1)	25238 (23.9)	0.02
Problems related to housing and economic circumstances; n (%)	937 (0.9)	875 (0.8)	0.006
COMORBIDITIES; n (%)			
Overweight and obesity	19293 (18.3)	18078 (17.1)	0.03
Hypertensive disease	28338 (26.8)	26182 (24.8)	0.05
Diabetes mellitus			
Type 1 diabetes mellitus	1995 (1.9)	1894 (1.8)	0.007
Type 2 diabetes mellitus	12133 (11.5)	11300 (10.7)	0.03
Chronic lower respiratory diseases			
Bronchitis; not specified as acute or chronic	7789 (7.4)	7566 (7.2)	0.008
Simple and mucopurulent chronic bronchitis	624 (0.6)	585 (0.6)	0.005
Unspecified chronic bronchitis	659 (0.6)	643 (0.6)	0.002
Emphysema	1779 (1.7)	1706 (1.6)	0.005
Other chronic obstructive pulmonary disease	5699 (5.4)	5498 (5.2)	0.008
Asthma	16643 (15.8)	15963 (15.1)	0.02
Bronchiectasis	672 (0.6)	656 (0.6)	0.002
Nicotine dependence	12134 (11.5)	11839 (11.2)	0.009
Substance misuse	15716 (14.9)	15182 (14.4)	0.01
Heart disease			
Ischemic heart diseases	7719 (7.3)	7340 (7.0)	0.01
Other forms of heart disease	16717 (15.8)	15685 (14.9)	0.03

Chronic kidney diseases			
Chronic kidney disease (CKD)	5471 (5.2)	5090 (4.8)	0.02
Hypertensive chronic kidney disease	3158 (3.0)	2904 (2.8)	0.01
Chronic liver disease			
Alcoholic liver disease	366 (0.3)	335 (0.3)	0.005
Hepatic failure; not elsewhere classified	459 (0.4)	414 (0.4)	0.007
Chronic hepatitis; not elsewhere classified	138 (0.1)	116 (0.1)	0.006
Fibrosis and cirrhosis of liver	907 (0.9)	820 (0.8)	0.009
Fatty (change of) liver; not elsewhere classified	3301 (3.1)	3103 (2.9)	0.01
Chronic passive congestion of liver	668 (0.6)	635 (0.6)	0.004
Portal hypertension	333 (0.3)	309 (0.3)	0.004
Other specified diseases of liver	2276 (2.2)	2168 (2.1)	0.007
Cerebral infarction	1780 (1.7)	1676 (1.6)	0.008
Dementia			
Vascular dementia	234 (0.2)	196 (0.2)	0.008
Dementia in other diseases classified elsewhere	382 (0.4)	339 (0.3)	0.007
Unspecified dementia	771 (0.7)	714 (0.7)	0.006
Alzheimer disease	287 (0.3)	257 (0.2)	0.006
Frontotemporal dementia	16 (0.01)	14 (0.01)	0.002
Dementia with Lewy bodies	22 (0.02)	26 (0.03)	0.003
Neoplasms			
Neoplasms (any)	20992 (19.9)	19481 (18.5)	0.04
Malignant neoplasms of lymphoid; hematopoietic and related tissue	1472 (1.4)	1360 (1.3)	0.009
Organ transplant			
Renal Transplantation Procedures	208 (0.2)	179 (0.2)	0.006
Liver Transplantation Procedures	51 (0.05)	42 (0.04)	0.004
Psoriasis	1359 (1.3)	1305 (1.2)	0.005
Rheumatoid arthritis			
Rheumatoid arthritis with rheumatoid factor	481 (0.5)	474 (0.4)	0.001
Other rheumatoid arthritis	1691 (1.6)	1572 (1.5)	0.009
Systemic lupus erythematosus (SLE)	707 (0.7)	673 (0.6)	0.004
Disorders involving the immune mechanism	2609 (2.5)	2502 (2.4)	0.007

Table S4 – Characteristics of the COVID-19 and other respiratory tract infection (RTI) cohorts after propensity-score matching. SMD=Standardized Mean Difference.

	COVID-19	Other RTI	SMD
Number	236038	236038	-
DEMOGRAPHICS			
Age; mean (SD); y	45.9 (19.7)	46.0 (20.4)	0.005
Sex; n (%)			
Female	131376 (55.7)	132927 (56.3)	0.01
Male	103760 (44.0)	102170 (43.3)	0.01
Other	902 (0.4)	941 (0.4)	0.003
Race; n (%)			
White	135106 (57.2)	137562 (58.3)	0.02
Black or African American	44394 (18.8)	42208 (17.9)	0.02
Asian	6972 (3.0)	6696 (2.8)	0.007
American Indian or Alaska Native	960 (0.4)	880 (0.4)	0.005
Native Hawaiian or Other Pacific Islander	741 (0.3)	706 (0.3)	0.003
Unknown	47865 (20.3)	47986 (20.3)	0.001
Ethnicity; n (%)			
Hispanic or Latino	37496 (15.9)	34960 (14.8)	0.03
Not Hispanic of Latino	134034 (56.8)	135033 (57.2)	0.009
Unknown	64508 (27.3)	66045 (28.0)	0.01
Problems related to housing and economic circumstances; n (%)	2438 (1.0)	2201 (0.9)	0.01
COMORBIDITIES; n (%)			
Overweight and obesity	42807 (18.1)	40343 (17.1)	0.03
Hypertensive disease	70868 (30.0)	66780 (28.3)	0.04
Diabetes mellitus			
Type 1 diabetes mellitus	4993 (2.1)	4468 (1.9)	0.02
Type 2 diabetes mellitus	36535 (15.5)	33881 (14.4)	0.03
Chronic lower respiratory diseases			
Bronchitis; not specified as acute or chronic	11033 (4.7)	11219 (4.8)	0.004
Simple and mucopurulent chronic bronchitis	988 (0.4)	1038 (0.4)	0.003
Unspecified chronic bronchitis	1190 (0.5)	1198 (0.5)	5.00E-04
Emphysema	3452 (1.5)	3434 (1.5)	6.00E-04
Other chronic obstructive pulmonary disease	10562 (4.5)	10301 (4.4)	0.005
Asthma	25100 (10.6)	24044 (10.2)	0.01
Bronchiectasis	1183 (0.5)	1172 (0.5)	7.00E-04
Nicotine dependence	17105 (7.2)	17173 (7.3)	0.001
Substance misuse	24862 (10.5)	24456 (10.4)	0.006
Heart disease			
Ischemic heart diseases	21021 (8.9)	19453 (8.2)	0.02

Other forms of heart disease	42331 (17.9)	39725 (16.8)	0.03
Chronic kidney diseases			
Chronic kidney disease (CKD)	15837 (6.7)	14406 (6.1)	0.02
Hypertensive chronic kidney disease	8885 (3.8)	8116 (3.4)	0.02
Chronic liver disease			
Alcoholic liver disease	1089 (0.5)	1018 (0.4)	0.005
Hepatic failure; not elsewhere classified	1400 (0.6)	1296 (0.5)	0.006
Chronic hepatitis; not elsewhere classified	309 (0.1)	283 (0.1)	0.003
Fibrosis and cirrhosis of liver	2524 (1.1)	2373 (1.0)	0.006
Fatty (change of) liver; not elsewhere classified	8179 (3.5)	7559 (3.2)	0.01
Chronic passive congestion of liver	1329 (0.6)	1217 (0.5)	0.006
Portal hypertension	1042 (0.4)	995 (0.4)	0.003
Other specified diseases of liver	5363 (2.3)	4994 (2.1)	0.01
Cerebral infarction	5826 (2.5)	5337 (2.3)	0.01
Dementia			
Vascular dementia	1264 (0.5)	1206 (0.5)	0.003
Dementia in other diseases classified elsewhere	2077 (0.9)	1948 (0.8)	0.006
Unspecified dementia	4557 (1.9)	4323 (1.8)	0.007
Alzheimer disease	1629 (0.7)	1544 (0.7)	0.004
Frontotemporal dementia	100 (0.04)	96 (0.04)	8.00E-04
Dementia with Lewy bodies	137 (0.06)	131 (0.06)	0.001
Neoplasms			
Neoplasms (any)	45215 (19.2)	43826 (18.6)	0.02
Malignant neoplasms of lymphoid; hematopoietic and related tissue	2654 (1.1)	2447 (1.0)	0.008
Organ transplant			
Renal Transplantation Procedures	727 (0.3)	640 (0.3)	0.007
Liver Transplantation Procedures	143 (0.06)	154 (0.07)	0.002
Psoriasis	2528 (1.1)	2366 (1.0)	0.007
Rheumatoid arthritis			
Rheumatoid arthritis with rheumatoid factor	972 (0.4)	847 (0.4)	0.009
Other rheumatoid arthritis	3327 (1.4)	3114 (1.3)	0.008
Systemic lupus erythematosus (SLE)	1416 (0.6)	1343 (0.6)	0.004
Disorders involving the immune mechanism	5198 (2.2)	4770 (2.0)	0.01

Table S5 – Hazard ratios and log-rank p-values estimated for the comparison between the COVID-19 cohort and the other two matched cohorts for subcategories of the main outcomes presented in Table 2 of the main manuscript. The corresponding ICD-10 codes are displayed in brackets.

	**COVID-19 vs. Influenza**		**COVID-19 vs. Other RTI**	
	HR (95% CI)	P-value	HR (95% CI)	P-value
Alcohol-related disorder (F10)	1.71 (1.54-1.90)	<0.0001	1.30 (1.20-1.40)	<0.0001
Opioid-related disorder (F11)	1.53 (1.32-1.76)	<0.0001	1.16 (1.05-1.29)	0.005
Cannabis-related disorder (F12)	1.33 (1.15-1.54)	0.00012	1.09 (0.97-1.23)	0.13
Nicotine dependence (F17)	1.14 (1.08-1.20)	<0.0001	0.98 (0.94-1.03)	0.49
Other substance misuse (F13–F16, F18, F19)	1.55 (1.38-1.75)	<0.0001	1.15 (1.05-1.25)	0.0021
Cranial nerve disorder (G50-G53)	1.36 (1.09-1.71)	0.005	1.08 (0.93-1.24)	0.3
Nerve root and plexus disorder (G54-G55)	1.76 (1.22-2.53)	0.0017	1.53 (1.20-1.94)	0.00039
Mononeuropathies (G56-G59)	1.68 (1.50-1.88)	<0.0001	1.33 (1.23-1.44)	<0.0001
Myastenia gravis and other myoneural disorders (G70)	3.88 (2.11-7.13)	<0.0001	2.19 (1.50-3.22)	<0.0001
Myastenia gravis	1.56 (0.55-4.46)	0.67	0.96 (0.43-2.15)	0.77
Myoneural disorder; unspecified	5.92 (2.99-11.75)	<0.0001	3.48 (2.16-5.61)	<0.0001
Primary disorders of muscles (G71)	0.43 (0.038-4.88)	0.88	1.60 (0.53-4.82)	0.34
Other and unspecified myopathies (G72)	6.02 (3.77-9.62)	<0.0001	4.31 (3.36-5.52)	<0.0001
Specified myopathies (G72.0-G72.4)	1.13 (0.35-3.60)	0.61	1.29 (0.69-2.41)	0.28
Critical illness myopathy and other specified myopathies (G72.8)	8.67 (4.75-15.81)	<0.0001	6.15 (4.50-8.40)	<0.0001
Myopathy; unspecified (G72.9)	3.09 (1.34-7.12)	0.0051	3.00 (1.79-5.01)	<0.0001
Myoneural junction/muscle disorder in diseases classified elsewhere (G73)	4.4e-09 (0.00-Inf)	0.39	1.24 (0.31-4.96)	0.37
Nontraumatic subarachnoid hemorrhage (I60)	2.19 (1.37-3.50)	0.00054	1.00 (0.79-1.27)	0.92
Nontraumatic intracerebral hemorrhage (I61)	2.17 (1.51-3.11)	<0.0001	1.11 (0.91-1.34)	0.28
Other and unspecified nontraumatic intracranial hemorrhage (I62)	3.01 (1.99-4.56)	<0.0001	1.32 (1.06-1.64)	0.009

Table S6 – Hazard ratios and log-rank p-values estimated for the comparison between the COVID-19 cohort and the additional four matched cohorts for the main neurologic and psychiatric outcomes presented in Table 2 of the main manuscript. PE=Pulmonary embolism

	**Skin infection** (n=176079)		**Urolithiasis** (n=125297)		**Fracture** (n=162759)		**PE** (n=51842)	
	HR (95% CI)	P-value	HR (95% CI)	P-value	HR (95% CI)	P-value	HR (95% CI)	P-value
Intracranial haemorrhage (any)	1.63 (1.39-1.91)	<0.0001	1.43 (1.21-1.70)	<0.0001	0.45 (0.40-0.51)	<0.0001	0.60 (0.51-0.70)	<0.0001
Intracranial haemorrhage (first)	1.62 (1.28-2.05)	<0.0001	1.92 (1.47-2.53)	<0.0001	0.44 (0.36-0.53)	<0.0001	0.72 (0.55-0.94)	0.017
Ischaemic stroke (any)	1.41 (1.30-1.52)	<0.0001	1.44 (1.32-1.56)	<0.0001	1.41 (1.31-1.52)	<0.0001	0.93 (0.86-1.01)	0.1
Ischaemic stroke (first)	1.55 (1.35-1.78)	<0.0001	2.18 (1.84-2.58)	<0.0001	1.40 (1.22-1.60)	<0.0001	0.88 (0.74-1.05)	0.16
Parkinsonism	1.28 (0.89-1.84)	0.16	1.47 (1.01-2.13)	0.036	0.78 (0.57-1.07)	0.2	1.57 (0.99-2.49)	0.039
Guillain-Barre syndrome	2.57 (1.64-4.03)	<0.0001	2.65 (1.62-4.33)	<0.0001	1.96 (1.32-2.91)	0.00077	1.47 (0.91-2.37)	0.11
Nerve/nerve root/plexus disorders	0.92 (0.86-0.98)	0.012	1.19 (1.10-1.28)	<0.0001	0.61 (0.57-0.65)	<0.0001	1.10 (1.01-1.21)	0.034
Myoneural junction/muscle disease	4.49 (3.51-5.74)	<0.0001	3.93 (3.04-5.09)	<0.0001	4.32 (3.37-5.54)	<0.0001	2.39 (1.82-3.15)	<0.0001
Encephalitis	1.50 (1.06-2.11)	0.02	1.36 (0.92-2.01)	0.094	1.69 (1.17-2.44)	0.0041	0.87 (0.59-1.28)	0.53
Dementia	1.48 (1.29-1.70)	<0.0001	3.38 (2.82-4.06)	<0.0001	1.06 (0.93-1.20)	0.36	2.34 (1.95-2.82)	<0.0001
Mood/Anxiety/Psychotic disorder (any)	1.14 (1.12-1.17)	<0.0001	1.24 (1.21-1.27)	<0.0001	1.25 (1.22-1.28)	<0.0001	1.09 (1.06-1.12)	<0.0001
Mood/Anxiety/Psychotic disorder (first)	1.31 (1.25-1.38)	<0.0001	1.59 (1.50-1.69)	<0.0001	1.41 (1.34-1.48)	<0.0001	1.18 (1.08-1.28)	0.00016
Mood disorder (any)	1.07 (1.04-1.10)	<0.0001	1.14 (1.10-1.17)	<0.0001	1.11 (1.07-1.14)	<0.0001	1.03 (0.99-1.08)	0.089
Mood disorder (first)	1.09 (1.02-1.17)	0.0068	1.28 (1.18-1.38)	<0.0001	1.17 (1.09-1.25)	<0.0001	1.04 (0.93-1.15)	0.47
Anxiety disorder (any)	1.18 (1.15-1.21)	<0.0001	1.22 (1.19-1.26)	<0.0001	1.33 (1.30-1.37)	<0.0001	1.08 (1.04-1.12)	<0.0001
Anxiety disorder (first)	1.38 (1.31-1.45)	<0.0001	1.57 (1.47-1.67)	<0.0001	1.53 (1.45-1.62)	<0.0001	1.18 (1.08-1.29)	0.00021
Psychotic disorder (any)	1.25 (1.15-1.36)	<0.0001	2.17 (1.93-2.44)	<0.0001	1.49 (1.36-1.63)	<0.0001	1.45 (1.28-1.64)	<0.0001
Psychotic disorder (first)	1.13 (0.95-1.35)	0.14	2.43 (1.91-3.10)	<0.0001	1.30 (1.08-1.56)	0.004	1.79 (1.34-2.38)	<0.0001
Substance misuse (any)	0.76 (0.74-0.79)	<0.0001	0.99 (0.95-1.04)	0.79	0.85 (0.82-0.88)	<0.0001	0.85 (0.81-0.89)	<0.0001
Substance misuse (first)	0.53 (0.49-0.57)	<0.0001	0.76 (0.69-0.84)	<0.0001	0.52 (0.48-0.56)	<0.0001	0.65 (0.56-0.76)	<0.0001
Insomnia (any)	1.23 (1.18-1.30)	<0.0001	1.35 (1.28-1.43)	<0.0001	1.33 (1.26-1.40)	<0.0001	1.10 (1.04-1.18)	0.0025
Insomnia (first)	1.49 (1.38-1.62)	<0.0001	1.68 (1.53-1.85)	<0.0001	1.46 (1.34-1.59)	<0.0001	1.06 (0.94-1.19)	0.32

Table S7 – P-values for the test of proportional hazards (obtained using the generalized Schoenfeld test) for the two primary control cohorts. A value lower than 0.05 indicates evidence for non-proportional hazards. RTI=Respiratory tract infections.

	**COVID-19 vs Influenza**	**COVID-19 vs Other RTI**
Intracranial haemorrhage (any)	0.11	0.012
Intracranial haemorrhage (first)	0.096	0.14
Ischaemic stroke (any)	0.24	0.032
Ischaemic stroke (first)	0.65	0.12
Parkinsonism	0.97	0.97
Guillain-Barre syndrome	0.076	0.11
Nerve/nerve root/plexus disorders	0.79	0.13
Myoneural junction/muscle disease	0.86	0.55
Encephalitis	0.62	0.28
Dementia	0.2	0.21
Mood/Anxiety/Psychotic disorder (any)	<0.0001	<0.0001
Mood/Anxiety/Psychotic disorder (first)	0.012	0.00052
Mood disorder (any)	0.0024	<0.0001
Mood disorder (first)	0.48	0.89
Anxiety disorder (any)	<0.0001	<0.0001
Anxiety disorder (first)	0.0033	<0.0001
Psychotic disorder (any)	0.12	0.78
Psychotic disorder (first)	0.87	0.036
Substance misuse (any)	0.92	0.56
Substance misuse (first)	0.4	0.1
Insomnia (any)	0.54	0.1
Insomnia (first)	0.039	0.059

Table S8 – P-values for the test of proportional hazards (obtained using the generalized Schoenfeld test) for the other four control cohorts. A value lower than 0.05 indicates evidence for non-proportional hazards. RTI=Respiratory tract infections.

	**COVID-19 vs Skin Infection**	**COVID-19 vs Urolithiasis**	**COVID-19 vs Fracture**	**COVID-19 vs PE**
Intracranial haemorrhage (any)	0.9	0.059	<0.0001	0.0017
Intracranial haemorrhage (first)	0.47	0.95	<0.0001	0.011
Ischaemic stroke (any)	0.011	0.21	0.19	<0.0001
Ischaemic stroke (first)	0.52	0.38	0.35	0.003
Parkinsonism	0.072	0.56	0.45	0.81
Guillain-Barre syndrome	0.023	0.71	0.0045	0.23
Nerve/nerve root/plexus disorders	<0.0001	<0.0001	<0.0001	<0.0001
Myoneural junction/muscle disease	0.55	1	0.19	0.83
Encephalitis	0.38	0.96	0.93	0.67
Dementia	0.00027	0.014	0.4	0.16
Mood/Anxiety/Psychotic disorder (any)	0.0016	0.45	<0.0001	<0.0001
Mood/Anxiety/Psychotic disorder (first)	0.57	0.045	<0.0001	0.0048
Mood disorder (any)	0.44	0.44	<0.0001	0.0043
Mood disorder (first)	0.0089	0.98	<0.0001	0.00075
Anxiety disorder (any)	0.00011	0.6	<0.0001	<0.0001
Anxiety disorder (first)	0.0082	0.012	0.00055	0.037
Psychotic disorder (any)	0.025	0.00066	0.25	0.76
Psychotic disorder (first)	0.58	0.086	0.18	0.12
Substance misuse (any)	<0.0001	0.58	<0.0001	<0.0001
Substance misuse (first)	0.0039	0.32	<0.0001	0.0081
Insomnia (any)	0.49	0.33	0.0017	0.00034
Insomnia (first)	0.0059	0.025	0.0081	0.15

Table S9 – Characteristics of the cohort of patients with COVID-19 not requiring hospitalization and the cohort of patients with influenza not requiring hospitalization, after propensity-score matching. SMD=Standardized Mean Difference.

	**COVID-19 (non-hospitalized)**	**Influenza (non-hospitalized)**	**SMD**
Number	96803	96803	-
DEMOGRAPHICS			
Age; mean (SD); y	38.6 (17.8)	37.4 (19.0)	0.07
Sex; n (%)			
Female	57328 (59.2)	56090 (57.9)	0.03
Male	39438 (40.7)	40670 (42.0)	0.03
Other	37 (0.04)	43 (0.04)	0.003
Race; n (%)			
White	63831 (65.9)	63465 (65.6)	0.008
Black or African American	17334 (17.9)	16553 (17.1)	0.02
Asian	3478 (3.6)	3239 (3.3)	0.01
American Indian or Alaska Native	404 (0.4)	400 (0.4)	6.00E-04
Native Hawaiian or Other Pacific Islander	274 (0.3)	229 (0.2)	0.009
Unknown	11482 (11.9)	12917 (13.3)	0.04
Ethnicity; n (%)			
Hispanic or Latino	8540 (8.8)	8474 (8.8)	0.002
Not Hispanic of Latino	65640 (67.8)	64819 (67.0)	0.02
Unknown	22623 (23.4)	23510 (24.3)	0.02
Problems related to housing and economic circumstances; n (%)	648 (0.7)	595 (0.6)	0.007
COMORBIDITIES; n (%)			
Overweight and obesity	16599 (17.1)	15630 (16.1)	0.03
Hypertensive disease	23247 (24.0)	21322 (22.0)	0.05
Diabetes mellitus			
Type 1 diabetes mellitus	1514 (1.6)	1414 (1.5)	0.008
Type 2 diabetes mellitus	9444 (9.8)	8752 (9.0)	0.02
Chronic lower respiratory diseases			
Bronchitis; not specified as acute or chronic	6728 (7.0)	6646 (6.9)	0.003
Simple and mucopurulent chronic bronchitis	464 (0.5)	454 (0.5)	0.002
Unspecified chronic bronchitis	458 (0.5)	449 (0.5)	0.001
Emphysema	1107 (1.1)	1054 (1.1)	0.005
Other chronic obstructive pulmonary disease	3458 (3.6)	3372 (3.5)	0.005
Asthma	14453 (14.9)	14020 (14.5)	0.01
Bronchiectasis	450 (0.5)	438 (0.5)	0.002
Nicotine dependence	9847 (10.2)	9693 (10.0)	0.005
Substance misuse	12840 (13.3)	12492 (12.9)	0.01
Heart disease			

Ischemic heart diseases	5456 (5.6)	4982 (5.1)	0.02
Other forms of heart disease	12647 (13.1)	11768 (12.2)	0.03
Chronic kidney diseases			
Chronic kidney disease (CKD)	3608 (3.7)	3374 (3.5)	0.01
Hypertensive chronic kidney disease	1966 (2.0)	1820 (1.9)	0.01
Chronic liver disease			
Alcoholic liver disease	255 (0.3)	224 (0.2)	0.006
Hepatic failure; not elsewhere classified	281 (0.3)	235 (0.2)	0.009
Chronic hepatitis; not elsewhere classified	99 (0.1)	84 (0.09)	0.005
Fibrosis and cirrhosis of liver	659 (0.7)	578 (0.6)	0.01
Fatty (change of) liver; not elsewhere classified	2816 (2.9)	2609 (2.7)	0.01
Chronic passive congestion of liver	516 (0.5)	496 (0.5)	0.003
Portal hypertension	246 (0.3)	203 (0.2)	0.009
Other specified diseases of liver	1862 (1.9)	1754 (1.8)	0.008
Cerebral infarction	1283 (1.3)	1135 (1.2)	0.01
Dementia			
Vascular dementia	142 (0.1)	129 (0.1)	0.004
Dementia in other diseases classified elsewhere	242 (0.2)	213 (0.2)	0.006
Unspecified dementia	446 (0.5)	388 (0.4)	0.009
Alzheimer disease	173 (0.2)	156 (0.2)	0.004
Frontotemporal dementia	17 (0.02)	10 (0.01)	0.006
Dementia with Lewy bodies	27 (0.03)	18 (0.02)	0.006
Neoplasms			
Neoplasms (any)	18338 (18.9)	17048 (17.6)	0.03
Malignant neoplasms of lymphoid; hematopoietic and related tissue	976 (1.0)	955 (1.0)	0.002
Organ transplant			
Renal Transplantation Procedures	100 (0.1)	106 (0.1)	0.002
Liver Transplantation Procedures	29 (0.03)	22 (0.02)	0.004
Psoriasis	1193 (1.2)	1171 (1.2)	0.002
Rheumatoid arthritis			
Rheumatoid arthritis with rheumatoid factor	392 (0.4)	384 (0.4)	0.001
Other rheumatoid arthritis	1283 (1.3)	1249 (1.3)	0.003
Systemic lupus erythematosus (SLE)	606 (0.6)	564 (0.6)	0.006
Disorders involving the immune mechanism	1987 (2.1)	1887 (1.9)	0.007

Table S10 – Characteristics of the cohort of patients with COVID-19 not requiring hospitalization and the cohort of patients with other respiratory tract infections (RTI) not requiring hospitalization, after propensity-score matching. SMD=Standardized Mean Difference.

	COVID-19 (non-hospitalized)	Other RTI (non-hospitalized)	SMD
Number	183731	183731	-
DEMOGRAPHICS			
Age; mean (SD); y	43.3 (19.0)	43.3 (19.6)	7.00E-04
Sex; n (%)			
Female	104084 (56.6)	105084 (57.2)	0.01
Male	78969 (43.0)	77943 (42.4)	0.01
Other	678 (0.4)	704 (0.4)	0.002
Race; n (%)			
White	105595 (57.5)	107903 (58.7)	0.03
Black or African American	32587 (17.7)	30676 (16.7)	0.03
Asian	5291 (2.9)	5083 (2.8)	0.007
American Indian or Alaska Native	702 (0.4)	586 (0.3)	0.01
Native Hawaiian or Other Pacific Islander	814 (0.4)	792 (0.4)	0.002
Unknown	38742 (21.1)	38691 (21.1)	7.00E-04
Ethnicity; n (%)			
Hispanic or Latino	27936 (15.2)	25912 (14.1)	0.03
Not Hispanic of Latino	102332 (55.7)	103612 (56.4)	0.01
Unknown	53463 (29.1)	54207 (29.5)	0.009
Problems related to housing and economic circumstances; n (%)	1371 (0.7)	1232 (0.7)	0.009
COMORBIDITIES; n (%)			
Overweight and obesity	29583 (16.1)	27662 (15.1)	0.03
Hypertensive disease	46219 (25.2)	42968 (23.4)	0.04
Diabetes mellitus			
Type 1 diabetes mellitus	2922 (1.6)	2572 (1.4)	0.02
Type 2 diabetes mellitus	21968 (12.0)	19685 (10.7)	0.04
Chronic lower respiratory diseases			
Bronchitis; not specified as acute or chronic	8476 (4.6)	8840 (4.8)	0.009
Simple and mucopurulent chronic bronchitis	653 (0.4)	720 (0.4)	0.006
Unspecified chronic bronchitis	711 (0.4)	793 (0.4)	0.007
Emphysema	1891 (1.0)	1895 (1.0)	2.00E-04
Other chronic obstructive pulmonary disease	5669 (3.1)	5627 (3.1)	0.001
Asthma	19226 (10.5)	18273 (9.9)	0.02
Bronchiectasis	691 (0.4)	730 (0.4)	0.003
Nicotine dependence	12391 (6.7)	12345 (6.7)	0.001
Substance misuse	17747 (9.7)	17330 (9.4)	0.008

Heart disease			
Ischemic heart diseases	11671 (6.4)	10483 (5.7)	0.03
Other forms of heart disease	25371 (13.8)	23320 (12.7)	0.03
Chronic kidney diseases			
Chronic kidney disease (CKD)	8205 (4.5)	7221 (3.9)	0.03
Hypertensive chronic kidney disease	4437 (2.4)	3905 (2.1)	0.02
Chronic liver disease			
Alcoholic liver disease	557 (0.3)	499 (0.3)	0.006
Hepatic failure; not elsewhere classified	631 (0.3)	592 (0.3)	0.004
Chronic hepatitis; not elsewhere classified	191 (0.1)	163 (0.09)	0.005
Fibrosis and cirrhosis of liver	1373 (0.7)	1219 (0.7)	0.01
Fatty (change of) liver; not elsewhere classified	5620 (3.1)	4997 (2.7)	0.02
Chronic passive congestion of liver	889 (0.5)	841 (0.5)	0.004
Portal hypertension	526 (0.3)	468 (0.3)	0.006
Other specified diseases of liver	3623 (2.0)	3229 (1.8)	0.02
Cerebral infarction	3093 (1.7)	2686 (1.5)	0.02
Dementia			
Vascular dementia	640 (0.3)	609 (0.3)	0.003
Dementia in other diseases classified elsewhere	1098 (0.6)	1002 (0.5)	0.007
Unspecified dementia	2157 (1.2)	1997 (1.1)	0.008
Alzheimer disease	867 (0.5)	815 (0.4)	0.004
Frontotemporal dementia	57 (0.03)	40 (0.02)	0.006
Dementia with Lewy bodies	79 (0.04)	70 (0.04)	0.002
Neoplasms			
Neoplasms (any)	33036 (18.0)	31974 (17.4)	0.02
Malignant neoplasms of lymphoid; hematopoietic and related tissue	1560 (0.8)	1491 (0.8)	0.004
Organ transplant			
Renal Transplantation Procedures	328 (0.2)	283 (0.2)	0.006
Liver Transplantation Procedures	66 (0.04)	67 (0.04)	3.00E-04
Psoriasis	1896 (1.0)	1701 (0.9)	0.01
Rheumatoid arthritis			
Rheumatoid arthritis with rheumatoid factor	704 (0.4)	672 (0.4)	0.003
Other rheumatoid arthritis	2220 (1.2)	2075 (1.1)	0.007
Systemic lupus erythematosus (SLE)	997 (0.5)	876 (0.5)	0.009
Disorders involving the immune mechanism	3321 (1.8)	2974 (1.6)	0.01

Table S11 – Characteristics of the cohort of patients with COVID-19 requiring hospitalization and the cohort of patients with COVID-19 not requiring hospitalization, after propensity-score matching. SMD=Standardized Mean Difference.

	COVID-19 with hospitalization	COVID-19 without hospitalization	SMD
Number	44927	44927	-
DEMOGRAPHICS			
Age; mean (SD); y	56.4 (18.7)	56.8 (18.2)	0.02
Sex; n (%)			
Female	23161 (51.6)	24064 (53.6)	0.04
Male	21697 (48.3)	20834 (46.4)	0.04
Other	69 (0.2)	29 (0.07)	0.03
Race; n (%)			
White	24757 (55.1)	25081 (55.8)	0.01
Black or African American	10150 (22.6)	10395 (23.1)	0.01
Asian	1511 (3.4)	1548 (3.4)	0.005
American Indian or Alaska Native	243 (0.5)	248 (0.6)	0.002
Native Hawaiian or Other Pacific Islander	162 (0.4)	144 (0.3)	0.007
Unknown	8104 (18.0)	7511 (16.7)	0.03
Ethnicity; n (%)			
Hispanic or Latino	8338 (18.6)	8779 (19.5)	0.02
Not Hispanic of Latino	26296 (58.5)	27081 (60.3)	0.04
Unknown	10293 (22.9)	9067 (20.2)	0.07
Problems related to housing and economic circumstances; n (%)	941 (2.1)	909 (2.0)	0.005
COMORBIDITIES; n (%)			
Overweight and obesity	11981 (26.7)	12624 (28.1)	0.03
Hypertensive disease	22199 (49.4)	22687 (50.5)	0.02
Diabetes mellitus			
Type 1 diabetes mellitus	1779 (4.0)	1774 (3.9)	6.00E-04
Type 2 diabetes mellitus	13127 (29.2)	13105 (29.2)	0.001
Chronic lower respiratory diseases			
Bronchitis; not specified as acute or chronic	2181 (4.9)	2278 (5.1)	0.01
Simple and mucopurulent chronic bronchitis	292 (0.7)	300 (0.7)	0.002
Unspecified chronic bronchitis	420 (0.9)	411 (0.9)	0.002
Emphysema	1365 (3.0)	1277 (2.8)	0.01
Other chronic obstructive pulmonary disease	4300 (9.6)	3958 (8.8)	0.03
Asthma	5070 (11.3)	5342 (11.9)	0.02
Bronchiectasis	426 (0.9)	419 (0.9)	0.002
Nicotine dependence	4222 (9.4)	4263 (9.5)	0.003
Substance misuse	6301 (14.0)	6453 (14.4)	0.01
Heart disease			
Ischemic heart diseases	8382 (18.7)	7973 (17.7)	0.02

Other forms of heart disease	15111 (33.6)	14913 (33.2)	0.009
Chronic kidney diseases			
Chronic kidney disease (CKD)	6675 (14.9)	6105 (13.6)	0.04
Hypertensive chronic kidney disease	3935 (8.8)	3498 (7.8)	0.04
Chronic liver disease			
Alcoholic liver disease	453 (1.0)	409 (0.9)	0.01
Hepatic failure; not elsewhere classified	663 (1.5)	551 (1.2)	0.02
Chronic hepatitis; not elsewhere classified	103 (0.2)	98 (0.2)	0.002
Fibrosis and cirrhosis of liver	991 (2.2)	900 (2.0)	0.01
Fatty (change of) liver; not elsewhere classified	2210 (4.9)	2278 (5.1)	0.007
Chronic passive congestion of liver	373 (0.8)	369 (0.8)	0.001
Portal hypertension	439 (1.0)	387 (0.9)	0.01
Other specified diseases of liver	1464 (3.3)	1479 (3.3)	0.002
Cerebral infarction	2387 (5.3)	2187 (4.9)	0.02
Dementia			
Vascular dementia	559 (1.2)	502 (1.1)	0.01
Dementia in other diseases classified elsewhere	901 (2.0)	853 (1.9)	0.008
Unspecified dementia	2130 (4.7)	1825 (4.1)	0.03
Alzheimer disease	696 (1.5)	664 (1.5)	0.006
Frontotemporal dementia	44 (0.1)	45 (0.1)	7.00E-04
Dementia with Lewy bodies	55 (0.1)	51 (0.1)	0.003
Neoplasms			
Neoplasms (any)	10456 (23.3)	10969 (24.4)	0.03
Malignant neoplasms of lymphoid; hematopoietic and related tissue	962 (2.1)	961 (2.1)	2.00E-04
Organ transplant			
Renal Transplantation Procedures	327 (0.7)	292 (0.7)	0.009
Liver Transplantation Procedures	63 (0.1)	55 (0.1)	0.005
Psoriasis	538 (1.2)	524 (1.2)	0.003
Rheumatoid arthritis			
Rheumatoid arthritis with rheumatoid factor	230 (0.5)	244 (0.5)	0.004
Other rheumatoid arthritis	960 (2.1)	997 (2.2)	0.006
Systemic lupus erythematosus (SLE)	359 (0.8)	367 (0.8)	0.002
Disorders involving the immune mechanism	1658 (3.7)	1658 (3.7)	0

Table S12 – Characteristics of the cohort of patients with COVID-19 requiring ITU admission in the acute phase of the illness and the cohort of patients with COVID-19 not requiring ITU admission, after propensity-score matching. SMD=Standardized Mean Difference.

	**COVID-19 with ITU admission**	**COVID-19 without ITU admission**	**SMD**
Number	8942	8942	-
DEMOGRAPHICS			
Age; mean (SD); y	59.0 (17.3)	60.1 (17.3)	0.06
Sex; n (%)			
Female	3742 (41.8)	3815 (42.7)	0.02
Male	5194 (58.1)	5123 (57.3)	0.02
Other	10 (0.1)	10 (0.1)	0
Race; n (%)			
White	4916 (55.0)	4913 (54.9)	7.00E-04
Black or African American	2184 (24.4)	2229 (24.9)	0.01
Asian	320 (3.6)	341 (3.8)	0.01
American Indian or Alaska Native	42 (0.5)	53 (0.6)	0.02
Native Hawaiian or Other Pacific Islander	24 (0.3)	21 (0.2)	0.007
Unknown	1456 (16.3)	1385 (15.5)	0.02
Ethnicity; n (%)			
Hispanic or Latino	2246 (25.1)	2329 (26.0)	0.02
Not Hispanic of Latino	5040 (56.4)	5026 (56.2)	0.003
Unknown	1656 (18.5)	1587 (17.7)	0.02
Problems related to housing and economic circumstances; n (%)	236 (2.6)	220 (2.5)	0.01
COMORBIDITIES; n (%)			
Overweight and obesity	3059 (34.2)	3125 (34.9)	0.02
Hypertensive disease	5567 (62.3)	5740 (64.2)	0.04
Diabetes mellitus			
Type 1 diabetes mellitus	621 (6.9)	565 (6.3)	0.03
Type 2 diabetes mellitus	3784 (42.3)	3780 (42.3)	9.00E-04
Chronic lower respiratory diseases			
Bronchitis; not specified as acute or chronic	511 (5.7)	537 (6.0)	0.01
Simple and mucopurulent chronic bronchitis	85 (1.0)	89 (1.0)	0.005
Unspecified chronic bronchitis	117 (1.3)	118 (1.3)	0.001
Emphysema	424 (4.7)	401 (4.5)	0.01
Other chronic obstructive pulmonary disease	1252 (14.0)	1178 (13.2)	0.02
Asthma	1130 (12.6)	1178 (13.2)	0.02
Bronchiectasis	128 (1.4)	118 (1.3)	0.01
Nicotine dependence	1041 (11.6)	1025 (11.5)	0.006
Substance misuse	1618 (18.1)	1586 (17.7)	0.009
Heart disease			

Ischemic heart diseases	2459 (27.5)	2338 (26.1)	0.03
Other forms of heart disease	4675 (52.3)	4619 (51.7)	0.01
Chronic kidney diseases			
Chronic kidney disease (CKD)	1939 (21.7)	1812 (20.3)	0.03
Hypertensive chronic kidney disease	1166 (13.0)	1084 (12.1)	0.03
Chronic liver disease			
Alcoholic liver disease	140 (1.6)	110 (1.2)	0.03
Hepatic failure; not elsewhere classified	313 (3.5)	217 (2.4)	0.06
Chronic hepatitis; not elsewhere classified	34 (0.4)	23 (0.3)	0.02
Fibrosis and cirrhosis of liver	298 (3.3)	265 (3.0)	0.02
Fatty (change of) liver; not elsewhere classified	550 (6.2)	531 (5.9)	0.009
Chronic passive congestion of liver	114 (1.3)	99 (1.1)	0.02
Portal hypertension	129 (1.4)	102 (1.1)	0.03
Other specified diseases of liver	400 (4.5)	389 (4.3)	0.006
Cerebral infarction	775 (8.7)	722 (8.1)	0.02
Dementia			
Vascular dementia	146 (1.6)	151 (1.7)	0.004
Dementia in other diseases classified elsewhere	193 (2.2)	187 (2.1)	0.005
Unspecified dementia	546 (6.1)	521 (5.8)	0.01
Alzheimer disease	127 (1.4)	139 (1.6)	0.01
Frontotemporal dementia	10 (0.1)	11 (0.1)	0.003
Dementia with Lewy bodies	10 (0.1)	10 (0.1)	0
Neoplasms			
Neoplasms (any)	2339 (26.2)	2419 (27.1)	0.02
Malignant neoplasms of lymphoid; hematopoietic and related tissue	253 (2.8)	228 (2.5)	0.02
Organ transplant			
Renal Transplantation Procedures	103 (1.2)	104 (1.2)	0.001
Liver Transplantation Procedures	18 (0.2)	22 (0.2)	0.009
Psoriasis	136 (1.5)	146 (1.6)	0.009
Rheumatoid arthritis			
Rheumatoid arthritis with rheumatoid factor	64 (0.7)	78 (0.9)	0.02
Other rheumatoid arthritis	227 (2.5)	237 (2.6)	0.007
Systemic lupus erythematosus (SLE)	78 (0.9)	86 (1.0)	0.009
Disorders involving the immune mechanism	385 (4.3)	427 (4.8)	0.02

Table S13 – Characteristics of the cohort of patients with COVID-19 and encephalopathy in the acute phase of the illness, and the cohort of patients with COVID-19 but without encephalopathy, after propensity-score matching. SMD=Standardized Mean Difference.

	**COVID-19 with encephalopathy**	**COVID-19 without encephalopathy**	**SMD**
Number	6221	6221	-
DEMOGRAPHICS			
Age; mean (SD); y	66.7 (17.0)	67.6 (16.0)	0.05
Sex; n (%)			
Female	2908 (46.7)	2929 (47.1)	0.007
Male	3300 (53.0)	3288 (52.9)	0.004
Other	13 (0.2)	10 (0.2)	0.01
Race; n (%)			
White	3330 (53.5)	3437 (55.2)	0.03
Black or African American	1549 (24.9)	1528 (24.6)	0.008
Asian	242 (3.9)	235 (3.8)	0.006
American Indian or Alaska Native	21 (0.3)	22 (0.4)	0.003
Native Hawaiian or Other Pacific Islander	11 (0.2)	10 (0.2)	0.004
Unknown	1068 (17.2)	990 (15.9)	0.03
Ethnicity; n (%)			
Hispanic or Latino	894 (14.4)	928 (14.9)	0.02
Not Hispanic of Latino	3867 (62.2)	3875 (62.3)	0.003
Unknown	1460 (23.5)	1418 (22.8)	0.02
Problems related to housing and economic circumstances; n (%)	215 (3.5)	195 (3.1)	0.02
COMORBIDITIES; n (%)			
Overweight and obesity	1832 (29.4)	2002 (32.2)	0.06
Hypertensive disease	4583 (73.7)	4853 (78.0)	0.1
Diabetes mellitus			
Type 1 diabetes mellitus	422 (6.8)	439 (7.1)	0.01
Type 2 diabetes mellitus	2884 (46.4)	2986 (48.0)	0.03
Chronic lower respiratory diseases			
Bronchitis; not specified as acute or chronic	362 (5.8)	412 (6.6)	0.03
Simple and mucopurulent chronic bronchitis	69 (1.1)	84 (1.4)	0.02
Unspecified chronic bronchitis	81 (1.3)	105 (1.7)	0.03
Emphysema	314 (5.0)	305 (4.9)	0.007
Other chronic obstructive pulmonary disease	1028 (16.5)	1055 (17.0)	0.01
Asthma	753 (12.1)	815 (13.1)	0.03
Bronchiectasis	89 (1.4)	97 (1.6)	0.01
Nicotine dependence	802 (12.9)	821 (13.2)	0.009
Substance misuse	1310 (21.1)	1347 (21.7)	0.01

Heart disease			
Ischemic heart diseases	2193 (35.3)	2221 (35.7)	0.009
Other forms of heart disease	3686 (59.3)	3834 (61.6)	0.05
Chronic kidney diseases			
Chronic kidney disease (CKD)	1886 (30.3)	1948 (31.3)	0.02
Hypertensive chronic kidney disease	1241 (19.9)	1223 (19.7)	0.007
Chronic liver disease			
Alcoholic liver disease	131 (2.1)	140 (2.2)	0.01
Hepatic failure; not elsewhere classified	280 (4.5)	258 (4.1)	0.02
Chronic hepatitis; not elsewhere classified	28 (0.5)	33 (0.5)	0.01
Fibrosis and cirrhosis of liver	246 (4.0)	268 (4.3)	0.02
Fatty (change of) liver; not elsewhere classified	353 (5.7)	364 (5.9)	0.008
Chronic passive congestion of liver	85 (1.4)	97 (1.6)	0.02
Portal hypertension	119 (1.9)	133 (2.1)	0.02
Other specified diseases of liver	293 (4.7)	314 (5.0)	0.02
Cerebral infarction	920 (14.8)	857 (13.8)	0.03
Dementia			
Vascular dementia	329 (5.3)	268 (4.3)	0.05
Dementia in other diseases classified elsewhere	545 (8.8)	413 (6.6)	0.08
Unspecified dementia	1196 (19.2)	977 (15.7)	0.09
Alzheimer disease	368 (5.9)	298 (4.8)	0.05
Frontotemporal dementia	23 (0.4)	19 (0.3)	0.01
Dementia with Lewy bodies	44 (0.7)	34 (0.5)	0.02
Neoplasms			
Neoplasms (any)	1790 (28.8)	1905 (30.6)	0.04
Malignant neoplasms of lymphoid; hematopoietic and related tissue	162 (2.6)	198 (3.2)	0.03
Organ transplant			
Renal Transplantation Procedures	46 (0.7)	57 (0.9)	0.02
Liver Transplantation Procedures	11 (0.2)	21 (0.3)	0.03
Psoriasis	82 (1.3)	79 (1.3)	0.004
Rheumatoid arthritis			
Rheumatoid arthritis with rheumatoid factor	40 (0.6)	36 (0.6)	0.008
Other rheumatoid arthritis	175 (2.8)	166 (2.7)	0.009
Systemic lupus erythematosus (SLE)	51 (0.8)	47 (0.8)	0.007
Disorders involving the immune mechanism	268 (4.3)	308 (5.0)	0.03

Table S14 – Hazard ratios and log-rank p-values estimated for the comparison between the COVID-19 cohorts with vs. without hospitalization in the primary analysis and when restricting the follow-up window to the 15-180 days (thus excluding the first 14 days).

	Hospitalization vs. not			
	1-180 days		15-180 days	
	HR (95% CI)	P-value	HR (95% CI)	P-value
Intracranial haemorrhage (any)	3.09 (2.43-3.94)	<0.0001	2.56 (1.96-3.33)	<0.0001
Intracranial haemorrhage (first)	3.75 (2.49-5.64)	<0.0001	2.62 (1.70-4.05)	<0.0001
Ischaemic stroke (any)	1.65 (1.48-1.85)	<0.0001	1.50 (1.32-1.70)	<0.0001
Ischaemic stroke (first)	2.82 (2.22-3.57)	<0.0001	2.17 (1.65-2.87)	<0.0001
Parkinsonism	2.63 (1.45-4.77)	0.0016	2.13 (1.07-4.24)	0.031
Guillain-Barre syndrome	2.94 (1.60-5.42)	0.00094	2.06 (1.15-3.68)	0.018
Nerve/nerve root/plexus disorders	0.94 (0.83-1.06)	0.29	0.96 (0.84-1.10)	0.56
Myoneural junction/muscle disease	7.76 (5.15-11.69)	<0.0001	6.22 (4.11-9.42)	<0.0001
Encephalitis	3.26 (1.75-6.06)	0.00017	2.54 (1.32-4.86)	0.0094
Dementia	2.28 (1.80-2.88)	<0.0001	1.60 (1.21-2.13)	0.0012
Mood/Anxiety/Psychotic disorder (any)	1.23 (1.18-1.28)	<0.0001	1.08 (1.03-1.13)	0.0012
Mood/Anxiety/Psychotic disorder (first)	1.55 (1.40-1.71)	<0.0001	1.45 (1.29-1.64)	<0.0001
Mood disorder (any)	1.21 (1.15-1.28)	<0.0001	1.06 (0.99-1.12)	0.077
Mood disorder (first)	1.53 (1.33-1.75)	<0.0001	1.40 (1.19-1.65)	<0.0001
Anxiety disorder (any)	1.16 (1.10-1.22)	<0.0001	1.05 (0.99-1.11)	0.11
Anxiety disorder (first)	1.49 (1.34-1.65)	<0.0001	1.38 (1.22-1.57)	<0.0001
Psychotic disorder (any)	2.22 (1.92-2.57)	<0.0001	2.00 (1.69-2.36)	<0.0001
Psychotic disorder (first)	2.77 (1.99-3.85)	<0.0001	3.26 (2.12-5.03)	<0.0001
Substance misuse (any)	1.53 (1.42-1.64)	<0.0001	1.32 (1.21-1.44)	<0.0001
Substance misuse (first)	1.68 (1.40-2.01)	<0.0001	1.39 (1.11-1.75)	0.0042
Insomnia (any)	1.08 (0.99-1.18)	0.088	1.02 (0.92-1.12)	0.76
Insomnia (first)	1.49 (1.28-1.74)	<0.0001	1.53 (1.29-1.83)	<0.0001

Table S15 – Hazard ratios and log-rank p-values estimated for the comparison between the COVID-19 cohorts with vs. without ITU admission in the primary analysis and when restricting the follow-up window to the 15-180 days (thus excluding the first 14 days).

	**ITU admission vs. not**			
	**1-180 days**		**15-180 days**	
	HR (95% CI)	P-value	HR (95% CI)	P-value
Intracranial haemorrhage (any)	5.06 (3.43-7.47)	<0.0001	4.43 (2.83-6.95)	<0.0001
Intracranial haemorrhage (first)	5.12 (2.68-9.77)	<0.0001	3.41 (1.67-6.96)	0.0015
Ischaemic stroke (any)	1.93 (1.62-2.31)	<0.0001	1.89 (1.54-2.32)	<0.0001
Ischaemic stroke (first)	3.51 (2.39-5.15)	<0.0001	3.92 (2.34-6.58)	<0.0001
Parkinsonism	3.90 (1.29-11.79)	0.024	2.66 (0.70-10.11)	0.18
Guillain-Barre syndrome	11.01 (2.55-47.61)	0.00073	4.61 (1.52-13.96)	0.0032
Nerve/nerve root/plexus disorders	1.16 (0.92-1.45)	0.21	1.29 (1.01-1.65)	0.04
Myoneural junction/muscle disease	11.53 (6.38-20.83)	<0.0001	17.60 (8.19-37.80)	<0.0001
Encephalitis	1.78 (0.75-4.20)	0.22	2.08 (0.79-5.49)	0.15
Dementia	1.66 (1.12-2.46)	0.018	1.69 (1.02-2.80)	0.092
Mood/Anxiety/Psychotic disorder (any)	1.34 (1.24-1.46)	<0.0001	1.30 (1.19-1.42)	<0.0001
Mood/Anxiety/Psychotic disorder (first)	2.27 (1.87-2.74)	<0.0001	2.26 (1.79-2.87)	<0.0001
Mood disorder (any)	1.15 (1.03-1.27)	0.01	1.12 (0.99-1.26)	0.07
Mood disorder (first)	2.06 (1.57-2.71)	<0.0001	2.27 (1.63-3.16)	<0.0001
Anxiety disorder (any)	1.39 (1.26-1.53)	<0.0001	1.35 (1.20-1.51)	<0.0001
Anxiety disorder (first)	2.22 (1.82-2.71)	<0.0001	2.23 (1.74-2.86)	<0.0001
Psychotic disorder (any)	1.48 (1.14-1.92)	0.0028	1.40 (1.04-1.88)	0.027
Psychotic disorder (first)	1.77 (0.98-3.20)	0.072	1.49 (0.71-3.14)	0.48
Substance misuse (any)	1.62 (1.41-1.85)	<0.0001	1.43 (1.22-1.68)	<0.0001
Substance misuse (first)	2.53 (1.83-3.50)	<0.0001	2.22 (1.46-3.36)	0.00027
Insomnia (any)	1.40 (1.19-1.66)	<0.0001	1.48 (1.23-1.80)	<0.0001
Insomnia (first)	1.93 (1.46-2.55)	<0.0001	2.54 (1.80-3.57)	<0.0001

Table S16 – Hazard ratios and log-rank p-values estimated for the comparison between the COVID-19 cohorts with vs. without encephalopathy in the primary analysis and when restricting the follow-up window to 15-180 days (thus excluding the first 14 days).

	Encephalopathy vs. not			
	1-180 days		15-180 days	
	HR (95% CI)	P-value	HR (95% CI)	P-value
Intracranial haemorrhage (any)	4.73 (3.15-7.11)	<0.0001	3.15 (2.06-4.81)	<0.0001
Intracranial haemorrhage (first)	5.00 (2.33-10.70)	<0.0001	2.45 (1.12-5.35)	0.037
Ischaemic stroke (any)	1.65 (1.38-1.97)	<0.0001	1.42 (1.15-1.75)	0.00092
Ischaemic stroke (first)	3.39 (2.17-5.29)	<0.0001	1.99 (1.19-3.34)	0.017
Parkinsonism	1.64 (0.75-3.58)	0.24	0.98 (0.32-2.95)	0.6
Guillain-Barre syndrome	2.27 (0.76-6.73)	0.24	3.29 (1.25-8.67)	0.16
Nerve/nerve root/plexus disorders	1.41 (1.07-1.87)	0.018	1.44 (1.05-1.98)	0.027
Myoneural junction/muscle disease	5.40 (3.21-9.07)	<0.0001	8.46 (4.04-17.70)	<0.0001
Encephalitis	9.98 (2.98-33.43)	<0.0001	4.78 (1.36-16.86)	0.018
Dementia	4.25 (2.79-6.47)	<0.0001	4.09 (2.40-6.98)	<0.0001
Mood/Anxiety/Psychotic disorder (any)	1.73 (1.58-1.90)	<0.0001	1.60 (1.44-1.78)	<0.0001
Mood/Anxiety/Psychotic disorder (first)	2.28 (1.80-2.89)	<0.0001	2.31 (1.69-3.15)	<0.0001
Mood disorder (any)	1.51 (1.35-1.70)	<0.0001	1.34 (1.17-1.53)	<0.0001
Mood disorder (first)	2.09 (1.55-2.80)	<0.0001	2.15 (1.49-3.09)	<0.0001
Anxiety disorder (any)	1.64 (1.45-1.84)	<0.0001	1.63 (1.42-1.87)	<0.0001
Anxiety disorder (first)	1.91 (1.48-2.45)	<0.0001	2.25 (1.61-3.14)	<0.0001
Psychotic disorder (any)	3.84 (2.90-5.10)	<0.0001	2.89 (2.12-3.94)	<0.0001
Psychotic disorder (first)	5.62 (2.93-10.77)	<0.0001	3.44 (1.68-7.02)	0.0013
Substance misuse (any)	1.45 (1.24-1.70)	<0.0001	1.49 (1.24-1.79)	<0.0001
Substance misuse (first)	2.03 (1.32-3.11)	0.0015	1.79 (0.99-3.22)	0.058
Insomnia (any)	1.73 (1.42-2.11)	<0.0001	2.11 (1.67-2.67)	<0.0001
Insomnia (first)	3.44 (2.35-5.04)	<0.0001	3.07 (2.03-4.63)	<0.0001

Table S17 – Mean (standard deviation) of the number of visits that each cohort of patients received during the follow-up period. Visits are counted for the matched cohorts; since the matched COVID-19 cohort varies from one comparison to the other, so does the number of visits.

	**COVID-19 cohort mean (SD) number of visits**	**Control cohort mean (SD) number of visits**
Influenza	4.05 (7.36)	4.23 (8.09)
Other RTI	3.94 (7.45)	4.21 (8.00)
Skin infection	4.13 (7.67)	5.05 (9.24)
Urolithiasis	4.49 (8.07)	5.27 (8.51)
Fracture or a large bone	3.96 (7.51)	5.89 (9.19)
Pulmonary embolism	6.14 (9.70)	10.55 (15.25)

Table S18: Hazard ratios for the major outcomes after COVID-19 compared to influenza and other respiratory infections (RTI) when patients who had died by the time of the analysis were included.

	COVID-19 vs. Influenza		COVID-19 vs. Other RTI	
	**HR (95% CI)**	**P-value**	**HR (95% CI)**	**P-value**
**Intracranial haemorrhage (any)**	2.16 (1.72-2.71)	<0.0001	1.18 (1.06-1.31)	0.0015
**Intracranial haemorrhage (first)**	2.29 (1.63-3.23)	<0.0001	1.54 (1.30-1.82)	<0.0001
**Ischaemic stroke (any)**	1.53 (1.37-1.72)	<0.0001	1.26 (1.19-1.34)	<0.0001
**Ischaemic stroke (first)**	1.67 (1.35-2.08)	<0.0001	1.40 (1.27-1.56)	<0.0001
**Parkinsonism**	2.21 (1.28-3.80)	0.0032	1.27 (0.97-1.66)	0.064
**Guillain-Barre syndrome**	1.76 (1.10-2.81)	0.015	1.99 (1.45-2.72)	<0.0001
**Nerve/nerve root/plexus disorders**	1.52 (1.38-1.66)	<0.0001	1.18 (1.11-1.24)	<0.0001
**Myoneural junction/muscle disease**	4.37 (3.14-6.08)	<0.0001	3.38 (2.82-4.04)	<0.0001
**Encephalitis**	1.20 (0.75-1.93)	0.38	1.69 (1.28-2.22)	0.00015
**Dementia**	2.26 (1.75-2.92)	<0.0001	1.56 (1.40-1.75)	<0.0001
**Mood/Anxiety/Psychotic disorder (any)**	1.44 (1.40-1.48)	<0.0001	1.16 (1.14-1.18)	<0.0001
**Mood/Anxiety/Psychotic disorder (first)**	1.76 (1.64-1.87)	<0.0001	1.38 (1.32-1.43)	<0.0001
**Mood disorder (any)**	1.44 (1.39-1.49)	<0.0001	1.18 (1.16-1.21)	<0.0001
**Mood disorder (first)**	1.67 (1.54-1.82)	<0.0001	1.30 (1.24-1.37)	<0.0001
**Anxiety disorder (any)**	1.44 (1.40-1.48)	<0.0001	1.13 (1.11-1.15)	<0.0001
**Anxiety disorder (first)**	1.75 (1.64-1.87)	<0.0001	1.40 (1.35-1.46)	<0.0001
**Psychotic disorder (any)**	1.93 (1.70-2.19)	<0.0001	1.52 (1.41-1.64)	<0.0001
**Psychotic disorder (first)**	1.99 (1.50-2.64)	<0.0001	1.61 (1.38-1.87)	<0.0001
**Substance misuse (any)**	1.20 (1.15-1.26)	<0.0001	1.06 (1.03-1.09)	0.00034
**Substance misuse (first)**	1.24 (1.11-1.38)	0.00012	0.92 (0.86-0.98)	0.0091
**Insomnia (any)**	1.42 (1.33-1.50)	<0.0001	1.08 (1.04-1.12)	<0.0001
**Insomnia (first)**	1.80 (1.62-2.00)	<0.0001	1.37 (1.28-1.46)	<0.0001

Table S19: Hazard ratios for the major outcomes after a diagnosis of COVID-19 confirmed using RNA/Antigen test compared to influenza and other respiratory infections (RTI).

	**COVID-19 vs. Influenza**		**COVID-19 vs. Other RTI**	
	**HR (95% CI)**	**P-value**	**HR (95% CI)**	**P-value**
**Intracranial haemorrhage (any)**	3.22 (2.49-4.17)	<0.0001	1.70 (1.44-2.00)	<0.0001
**Intracranial haemorrhage (first)**	3.97 (2.62-6.02)	<0.0001	2.56 (1.95-3.37)	<0.0001
**Ischaemic stroke (any)**	1.77 (1.57-2.00)	<0.0001	1.56 (1.43-1.71)	<0.0001
**Ischaemic stroke (first)**	2.53 (2.04-3.15)	<0.0001	2.09 (1.79-2.45)	<0.0001
**Parkinsonism**	2.39 (1.38-4.14)	0.0015	2.12 (1.42-3.17)	0.00017
**Guillain-Barre syndrome**	1.68 (1.03-2.76)	0.031	2.78 (1.74-4.45)	<0.0001
**Nerve/nerve root/plexus disorders**	1.49 (1.35-1.65)	<0.0001	1.23 (1.13-1.34)	<0.0001
**Myoneural junction/muscle disease**	7.12 (4.82-10.50)	<0.0001	5.44 (4.11-7.20)	<0.0001
**Encephalitis**	1.94 (1.17-3.20)	0.0084	1.76 (1.17-2.64)	0.0056
**Dementia**	3.42 (2.64-4.44)	<0.0001	3.05 (2.59-3.59)	<0.0001
**Mood/Anxiety/Psychotic disorder (any)**	1.41 (1.37-1.45)	<0.0001	1.20 (1.17-1.23)	<0.0001
**Mood/Anxiety/Psychotic disorder (first)**	1.92 (1.79-2.05)	<0.0001	1.59 (1.50-1.68)	<0.0001
**Mood disorder (any)**	1.40 (1.35-1.46)	<0.0001	1.24 (1.20-1.28)	<0.0001
**Mood disorder (first)**	1.82 (1.66-2.00)	<0.0001	1.57 (1.46-1.70)	<0.0001
**Anxiety disorder (any)**	1.39 (1.34-1.44)	<0.0001	1.15 (1.11-1.18)	<0.0001
**Anxiety disorder (first)**	1.87 (1.74-2.01)	<0.0001	1.51 (1.43-1.60)	<0.0001
**Psychotic disorder (any)**	2.27 (1.98-2.61)	<0.0001	1.77 (1.59-1.98)	<0.0001
**Psychotic disorder (first)**	2.70 (2.00-3.64)	<0.0001	1.85 (1.50-2.29)	<0.0001
**Substance misuse (any)**	1.23 (1.17-1.30)	<0.0001	1.10 (1.05-1.16)	<0.0001
**Substance misuse (first)**	1.28 (1.14-1.44)	<0.0001	1.02 (0.93-1.12)	0.69
**Insomnia (any)**	1.41 (1.32-1.51)	<0.0001	1.19 (1.13-1.26)	<0.0001
**Insomnia (first)**	1.98 (1.77-2.23)	<0.0001	1.57 (1.43-1.72)	<0.0001

Table S20: Hazard ratios for the major outcomes after a diagnosis of COVID-19 (in 2020) compared to influenza (in 2019 or 2018).

	**COVID-19 (2020) vs. Influenza (2019)**		**COVID-19 (2020) vs. Influenza (2018)**	
	**HR (95% CI)**	**P-value**	**HR (95% CI)**	**P-value**
**Intracranial haemorrhage (any)**	2.59 (2.06-3.27)	<0.0001	2.00 (1.62-2.48)	<0.0001
**Intracranial haemorrhage (first)**	3.02 (2.09-4.36)	<0.0001	2.13 (1.54-2.94)	<0.0001
**Ischaemic stroke (any)**	1.86 (1.67-2.07)	<0.0001	1.76 (1.58-1.95)	<0.0001
**Ischaemic stroke (first)**	1.91 (1.57-2.32)	<0.0001	2.09 (1.74-2.51)	<0.0001
**Parkinsonism**	1.36 (0.81-2.27)	0.17	1.46 (0.92-2.31)	0.065
**Guillain-Barre syndrome**	2.99 (1.78-5.02)	<0.0001	1.42 (0.88-2.28)	0.13
**Nerve/nerve root/plexus disorders**	1.66 (1.53-1.81)	<0.0001	1.51 (1.40-1.63)	<0.0001
**Myoneural junction/muscle disease**	3.41 (2.57-4.52)	<0.0001	3.51 (2.68-4.59)	<0.0001
**Encephalitis**	2.01 (1.32-3.07)	0.00085	2.78 (1.88-4.10)	<0.0001
**Dementia**	1.40 (1.13-1.73)	0.0015	1.88 (1.56-2.26)	<0.0001
**Mood/Anxiety/Psychotic disorder (any)**	1.82 (1.78-1.87)	<0.0001	1.92 (1.88-1.96)	<0.0001
**Mood/Anxiety/Psychotic disorder (first)**	2.21 (2.08-2.35)	<0.0001	2.19 (2.08-2.31)	<0.0001
**Mood disorder (any)**	1.76 (1.70-1.82)	<0.0001	1.83 (1.78-1.89)	<0.0001
**Mood disorder (first)**	1.90 (1.76-2.05)	<0.0001	1.92 (1.80-2.06)	<0.0001
**Anxiety disorder (any)**	1.90 (1.84-1.96)	<0.0001	2.05 (2.00-2.11)	<0.0001
**Anxiety disorder (first)**	2.33 (2.19-2.48)	<0.0001	2.27 (2.15-2.39)	<0.0001
**Psychotic disorder (any)**	2.20 (1.94-2.48)	<0.0001	2.23 (1.98-2.50)	<0.0001
**Psychotic disorder (first)**	1.95 (1.51-2.51)	<0.0001	2.55 (2.01-3.23)	<0.0001
**Substance misuse (any)**	1.32 (1.27-1.38)	<0.0001	1.34 (1.28-1.39)	<0.0001
**Substance misuse (first)**	1.51 (1.36-1.67)	<0.0001	1.41 (1.29-1.53)	<0.0001
**Insomnia (any)**	1.68 (1.59-1.78)	<0.0001	1.71 (1.63-1.80)	<0.0001
**Insomnia (first)**	2.15 (1.95-2.38)	<0.0001	2.06 (1.89-2.24)	<0.0001

References

1 Taquet M, Luciano S, Geddes JR, Harrison PJ. Bidirectional associations between COVID-19 and psychiatric disorder: retrospective cohort studies of 62 354 COVID-19 cases in the USA. Lancet Psychiatry 2021; 8:133-40.

2 Lusignan S de, de Lusignan S, Dorward J, et al. Risk factors for SARS-CoV-2 among patients in the Oxford Royal College of General Practitioners Research and Surveillance Centre primary care network: a cross-sectional study. The Lancet Infectious Diseases. 2020. DOI:10.1016/s1473-3099(20)30371-6.

3 Zhang J-J, Dong X, Cao Y-Y, et al. Clinical characteristics of 140 patients infected with SARS-CoV-2 in Wuhan, China. Allergy 2020. https://onlinelibrary.wiley.com/doi/abs/10.1111/all.14238.

4 Chen N, Zhou M, Dong X, et al. Epidemiological and clinical characteristics of 99 cases of 2019 novel coronavirus pneumonia in Wuhan, China: a descriptive study. Lancet 2020; 395: 507–13.

5 Wang QQ, Kaelber DC, Xu R, Volkow ND. Correction: COVID-19 risk and outcomes in patients with substance use disorders: analyses from electronic health records in the United States. Mol Psychiatry 2020; published online Sept 30. DOI:10.1038/s41380-020-00895-0.

6 Williamson EJ, Walker AJ, Bhaskaran K, et al. Factors associated with COVID-19-related death using OpenSAFELY. Nature 2020; 584: 430–6.

7 Slooter AJC, Otte WM, Devlin JW, et al. Updated nomenclature of delirium and acute encephalopathy: statement of ten Societies. Intensive Care Med 2020; 46: 1020–2.

8 Royston P, Parmar MKB. Flexible parametric proportional-hazards and proportional-odds models for censored survival data, with application to prognostic modelling and estimation of treatment effects. Statistics in Medicine. 2002; 21: 2175–97.

9 Liu X-R, Pawitan Y, Clements M. Parametric and penalized generalized survival models. Stat Methods Med Res 2018; 27: 1531–46.

The RECORD statement – checklist of items, extended from the STROBE statement, that should be reported in observational studies using routinely collected health data.

	Item No.	STROBE items	Location in manuscript where items are reported	RECORD items	Location in manuscript where items are reported
**Title and abstract**					
	1	(a) Indicate the study's design with a commonly used term in the title or the abstract (b) Provide in the abstract an informative and balanced summary of what was done and what was found	Title and Abstract	RECORD 1.1: The type of data used should be specified in the title or abstract. When possible, the name of the databases used should be included.<br><br>RECORD 1.2: If applicable, the geographic region and timeframe within which the study took place should be reported in the title or abstract.<br><br>RECORD 1.3: If linkage between databases was conducted for the study, this should be clearly stated in the title or abstract.	Page 1 and Abstract<br><br>Timeframe implicit in title; details on pp 2-3; geographic information in Appendix p1<br><br>n/a
**Introduction**					
Background rationale	2	Explain the scientific background and rationale for the investigation being reported	Abstract, Introduction, Research in Context		Abstract, Introduction, Research in Context
Objectives	3	State specific objectives, including any prespecified hypotheses	Abstract, Introduction		Abstract, Introduction
**Methods**					
Study Design	4	Present key elements of study design early in the paper	Abstract, Methods, Appendix		Abstract, Methods and Appendix
Setting	5	Describe the setting, locations, and relevant dates, including periods of recruitment, exposure, follow-up, and data collection	Abstract, Methods, Appendix		Abstract, Methods and Appendix

Participants	6	*(a) Cohort study* - Give the eligibility criteria, and the sources and methods of selection of participants. Describe methods of follow-up<br>*Case-control study* - Give the eligibility criteria, and the sources and methods of case ascertainment and control selection. Give the rationale for the choice of cases and controls<br>*Cross-sectional study* - Give the eligibility criteria, and the sources and methods of selection of participants<br><br>*(b) Cohort study* - For matched studies, give matching criteria and number of exposed and unexposed<br>*Case-control study* - For matched studies, give matching criteria and the number of controls per case	Methods and Appendix.<br><br>Methods, Appendix	RECORD 6.1: The methods of study population selection (such as codes or algorithms used to identify subjects) should be listed in detail. If this is not possible, an explanation should be provided.<br><br>RECORD 6.2: Any validation studies of the codes or algorithms used to select the population should be referenced. If validation was conducted for this study and not published elsewhere, detailed methods and results should be provided.<br><br>RECORD 6.3: If the study involved linkage of databases, consider use of a flow diagram or other graphical display to demonstrate the data linkage process, including the number of individuals with linked data at each stage.	Methods and Appendix. Patients selected based on ICD-10 codes.<br><br>Appendix<br><br>n/a
Variables	7	Clearly define all outcomes, exposures, predictors, potential confounders, and effect modifiers. Give diagnostic criteria, if applicable.	Methods, Appendix	RECORD 7.1: A complete list of codes and algorithms used to classify exposures, outcomes, confounders, and effect modifiers should be provided. If these cannot be reported, an explanation should be provided.	Methods, and Appendix
Data sources/ measurement	8	For each variable of interest, give sources of data and details of methods of assessment (measurement).<br>Describe comparability of assessment methods if there is more than one group	Methods, Appendix		

Bias	9	Describe any efforts to address potential sources of bias	Methods, Appendix		
Study size	10	Explain how the study size was arrived at	Methods, Appendix		
Quantitative variables	11	Explain how quantitative variables were handled in the analyses. If applicable, describe which groupings were chosen, and why	Methods, Appendix		
Statistical methods	12	(a) Describe all statistical methods, including those used to control for confounding<br>(b) Describe any methods used to examine subgroups and interactions<br>(c) Explain how missing data were addressed<br>(d) *Cohort study* - If applicable, explain how loss to follow-up was addressed<br>*Case-control study* - If applicable, explain how matching of cases and controls was addressed<br>*Cross-sectional study* - If applicable, describe analytical methods taking account of sampling strategy<br>(e) Describe any sensitivity analyses	Methods, Appendix		
Data access and cleaning methods		..		RECORD 12.1: Authors should describe the extent to which the investigators had access to the database population used to create the study population.	Author contribution section; Appendix

				RECORD 12.2: Authors should provide information on the data cleaning methods used in the study.	Appendix
Linkage		..		RECORD 12.3: State whether the study included person-level, institutional-level, or other data linkage across two or more databases. The methods of linkage and methods of linkage quality evaluation should be provided.	n/a
**Results**					
Participants	13	(a) Report the numbers of individuals at each stage of the study (*e.g.*, numbers potentially eligible, examined for eligibility, confirmed eligible, included in the study, completing follow-up, and analysed)<br>(b) Give reasons for non-participation at each stage.<br>(c) Consider use of a flow diagram	Results, Table 1, Supplementary Tables S1, S3, S4, S7, S9-S13	RECORD 13.1: Describe in detail the selection of the persons included in the study (*i.e.,* study population selection) including filtering based on data quality, data availability and linkage. The selection of included persons can be described in the text and/or by means of the study flow diagram.	Methods, Results, Table 1, Supplementary Tables S1, S3, S4, S7, S9-S13
Descriptive data	14	(a) Give characteristics of study participants (*e.g.*, demographic, clinical, social) and information on exposures and potential confounders<br>(b) Indicate the number of participants with missing data for each variable of interest<br>(c) *Cohort study* - summarise follow-up time (*e.g.*, average and total amount)	Table 1<br><br>Table 1<br><br>Figs 1 and 2		
Outcome data	15	*Cohort study* - Report numbers of outcome events or summary measures over time<br>*Case-control study* - Report numbers in each exposure	Results, Tables 1-4, Supplementary Tables and Figures		

		category, or summary measures of exposure<br>*Cross-sectional study* - Report numbers of outcome events or summary measures			
Main results	16	(a) Give unadjusted estimates and, if applicable, confounder-adjusted estimates and their precision (e.g., 95% confidence interval). Make clear which confounders were adjusted for and why they were included<br>(b) Report category boundaries when continuous variables were categorized<br>(c) If relevant, consider translating estimates of relative risk into absolute risk for a meaningful time period	Results, Tables 1-4 and Figures 1-2, Supplementary Figures and Tables<br>n/a<br>n/a		
Other analyses	17	Report other analyses done—e.g., analyses of subgroups and interactions, and sensitivity analyses	Methods, Tables 2-4, Supplementary Figures and Tables		
**Discussion**					
Key results	18	Summarise key results with reference to study objectives	Discussion, first paragraph		
Limitations	19	Discuss limitations of the study, taking into account sources of potential bias or imprecision. Discuss both direction and magnitude of any potential bias	Discussion, final paragraph	RECORD 19.1: Discuss the implications of using data that were not created or collected to answer the specific research question(s). Include discussion of misclassification bias, unmeasured confounding, missing data, and changing eligibility over time, as they pertain to the study being reported.	Discussion
Interpretation	20	Give a cautious overall interpretation of results considering objectives,	Abstract, Discussion		

		limitations, multiplicity of analyses, results from similar studies, and other relevant evidence			
Generalisability	21	Discuss the generalisability (external validity) of the study results	Discussion		
**Other Information**					
Funding	22	Give the source of funding and the role of the funders for the present study and, if applicable, for the original study on which the present article is based	Acknowledgment, Declarations of interest		
Accessibility of protocol, raw data, and programming code		..		RECORD 22.1: Authors should provide information on how to access any supplemental information such as the study protocol, raw data, or programming code.	Data sharing section

*Reference: Benchimol EI, Smeeth L, Guttmann A, Harron K, Moher D, Petersen I, Sørensen HT, von Elm E, Langan SM, the RECORD Working Committee. The REporting of studies Conducted using Observational Routinely-collected health Data (RECORD) Statement. *PLoS Medicine* 2015; in press.

*Checklist is protected under Creative Commons Attribution (CC BY) license.
